# Pharmacological Properties of Trichostatin A, Focusing on the Anticancer Potential: A Comprehensive Review

**DOI:** 10.3390/ph15101235

**Published:** 2022-10-08

**Authors:** Abdelhakim Bouyahya, Nasreddine El Omari, Mohamed Bakha, Tarik Aanniz, Naoual El Menyiy, Naoufal El Hachlafi, Aicha El Baaboua, Mohamed El-Shazly, Mohammed Merae Alshahrani, Ahmed Abdullah Al Awadh, Learn-Han Lee, Taoufiq Benali, Mohammad S. Mubarak

**Affiliations:** 1 Laboratory of Human Pathologies Biology, Department of Biology, Faculty of Sciences, Mohammed V University in Rabat, Rabat 10106, Morocco; 2 Laboratory of Histology, Embryology, and Cytogenetic, Faculty of Medicine and Pharmacy, Mohammed V University in Rabat, Rabat 10100, Morocco; 3 Unit of Plant Biotechnology and Sustainable Development of Natural Resources “B2DRN”, Polydisciplinary Faculty of Beni Mellal, Sultan Moulay Slimane University, Mghila, P.O. Box 592, Beni Mellal 23000, Morocco; 4 Medical Biotechnology Laboratory, Rabat Medical & Pharmacy School, Mohammed V University in Rabat, Rabat B.P. 6203, Morocco; 5 Laboratory of Pharmacology, National Agency of Medicinal and Aromatic Plants, Taounate 34025, Morocco; 6 Microbial Biotechnology and Bioactive Molecules Laboratory, Sciences and Technologies Faculty, Sidi Mohmed Ben Abdellah University, Imouzzer Road Fez, Fez 30050, Morocco; 7 Biotechnology and Applied Microbiology Team, Department of Biology, Faculty of Sciences, Abdelmalek Essaadi University, Tetouan 93000, Morocco; 8 Department of Pharmacognosy, Faculty of Pharmacy, Ain-Shams University, Cairo 11566, Egypt; 9 Department of Clinical Laboratory Sciences, Faculty of Applied Medical Sciences, Najran University, Najran 61441, Saudi Arabia; 10 Novel Bacteria and Drug Discovery Research Group (NBDD), Microbiome and Bioresource Research Strength (MBRS), Jeffrey Cheah School of Medicine and Health Sciences, Monash University Malaysia, Bandar Sunway 47500, Malaysia; 11 Environment and Health Team, Polydisciplinary Faculty of Safi, Cadi Ayyad University, Sidi Bouzid B.P. 4162, Morocco; 12 Department of Chemistry, The University of Jordan, Amma 11942, Jordan

**Keywords:** Trichostatin A, pharmacological activity, anticancer action, molecular mechanisms, epidrug

## Abstract

Trichostatin A (TSA), a natural derivative of dienohydroxamic acid derived from a fungal metabolite, exhibits various biological activities. It exerts antidiabetic activity and reverses high glucose levels caused by the downregulation of brain-derived neurotrophic factor (BDNF) expression in Schwann cells, anti-inflammatory activity by suppressing the expression of various cytokines, and significant antioxidant activity by suppressing oxidative stress through multiple mechanisms. Most importantly, TSA exhibits potent inhibitory activity against different types of cancer through different pathways. The anticancer activity of TSA appeared in many *in vitro* and *in vivo* investigations that involved various cell lines and animal models. Indeed, TSA exhibits anticancer properties alone or in combination with other drugs used in chemotherapy. It induces sensitivity of some human cancers toward chemotherapeutical drugs. TSA also exhibits its action on epigenetic modulators involved in cell transformation, and therefore it is considered an epidrug candidate for cancer therapy. Accordingly, this work presents a comprehensive review of the most recent developments in utilizing this natural compound for the prevention, management, and treatment of various diseases, including cancer, along with the multiple mechanisms of action. In addition, this review summarizes the most recent and relevant literature that deals with the use of TSA as a therapeutic agent against various diseases, emphasizing its anticancer potential and the anticancer molecular mechanisms. Moreover, TSA has not been involved in toxicological effects on normal cells. Furthermore, this work highlights the potential utilization of TSA as a complementary or alternative medicine for preventing and treating cancer, alone or in combination with other anticancer drugs.

## 1. Introduction

The search for natural news with a pharmaceutical interest is supported today by several investigations. Indeed, natural molecules from different sources have been tested for their biological effects and pharmacological properties. The screening of natural substances with pharmacological properties involves *in vitro* screening, *in vivo* investigations, and clinical trials, including toxicological tests to validate the harmlessness of bioactive molecules [1,2,3,4,5]. Indeed, indeed, molecules of natural origin are favored over synthetic ones. This advantage is essentially due to the high toxicity of synthetic molecules. However, the toxicity assessment must also be done for natural molecules because some show specific toxic effects [6,7,8].

**Figure 1:**
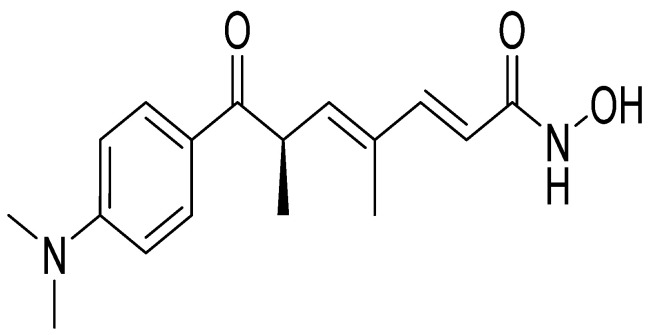
Chemical structure of trichostatin A.

Trichostatin A (TSA) (Figure 1) is a hydroxamic acid initially isolated from the secondary metabolites of *Streptomyces hygroscopicus* strains [9]. TSA is a known inhibitor of the canonical class I and class II histone deacetylases (HDACs) and one of the most promising agents with validated targets that prevent the progression of tumors [10]. The chemical structure of this compound plays an important role in its multiple biochemical and biological effects. TSA displays several pharmacological and physical properties, including antioxidant [11,12,13,14], antidiabetic [15,16,17], anti-inflammatory [18,19,20,21,22], and anticancer activities [23,24,25,26,27,28,29].

The antidiabetic effects of TSA are mainly related to its capacity to inhibit enzymes involved in the metabolism of glucides and lipids, as well as the signaling pathways involved in metabolism regulation [15,16,17]. On the other hand, the anti-inflammatory effects of this bioactive compound involve the inhibition of cytokines and other mediators of inflammatory processes [18,19,20,21,22].

Numerous reports focused on TSA’s *in vitro* and *in vivo* anti-tumor activity. These reports suggested four anticancer mechanisms of this substance. It can act directly on cell proliferation to activate the caspase pathway (inducing apoptosis) [30,31,32] and/or autophagy [33,34,35].

In addition, several reports indicated that TSA acts as a potent chemosensitizer on human tumors to improve chemosensitivity toward many drugs [36,37,38,39,40,41], and numerous studies highlighted the synergistic effect of TSA with chemotherapy [42,43,44,45,45]. Other reports emphasized the potent TSA effect in cancer epigenetic modifications [46,47,48,49]. Even though many studies usually reported the anticancer effect of TSA, to the best of our knowledge, no review was published to outline these reports critically and suggest future potential applications of this molecule as a promising agent in cancer therapy.

Although some investigations have already focused on the biological effects and pharmacological properties of TSA, to the best of our knowledge, critical reviews on the development of TSA as anticancer drugs with other properties such as anti-inflammatory and antioxidant effects have not yet been reported. Accordingly, and owing to the wide range of preventive and therapeutic options of TSA against different types of cancer, this review highlights the chemopreventive and therapeutic ability of this natural compound and the mechanisms of its action with a list of related references. Moreover, the antioxidant, antidiabetic, and anti-inflammatory activities of TSA were also highlighted and discussed.

## 2. Research Methodology

In this review, bibliometric research was done globally, without exclusion and/or inclusion criteria, from several databases, including Science Direct, PubMed, Google Scholar, Scopus, Wiley Online, and Web of Sciences.

Different keywords, including trichostatin A, the antioxidant activity of trichostatin A, anticancer activity of trichostatin A, antidiabetic activity of Trichostatin A”, and anti-inflammatory activity of trichostatin A were used to obtain relevant literature. The data collected were classified according to different sections (sources and different biological and pharmacological properties). Then, the publications of each section were organized in tables and explored. These data were finally discussed and highlighted. The molecular structure of cirsimaritin was designed using the Chem-Draw program.

## 3. Antioxidant Properties of TSA

The antioxidant activity of TSA was reported in several studies using several *in vitro* models [11,12,13,14] (Table 1). Yang et al. [14] evaluated the antioxidant activity of TSA against TGF-*β*-induced ROS accumulation in telomerase-immortalized human corneal fibroblasts. These authors found that TSA exhibits antioxidant activity manifested by the reduction in MDA levels, the elevation of intracellular glutathione (GSH) level and cellular total antioxidant capacity, and the decrease in cellular ROS and H_2_O_2_ accumulation. This compound also induced Nrf_2_ nuclear translocation and the upregulation of the expression of Nrf_2_-ARE downstream antioxidant genes.

**Figure 2:**
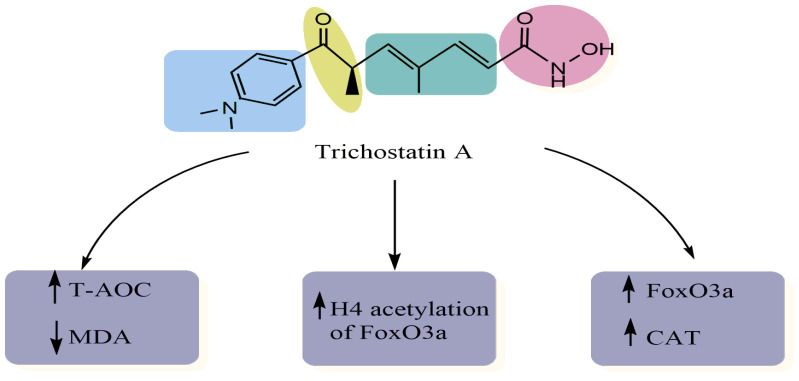
Antioxidant actions of TSA.

Using immunoblot analysis and the (3-(4,5-dimethylthiazol-2-yl)-2,5-diphenyltetrazolium bromide (MTT) assay, Jeong and colleagues examined the antioxidant potential of TSA in human bone marrow-mesenchymal stem cells (hBM-MSCs) after exposure to hydrogen peroxide (H_2_O_2_) [12]. These researchers showed that TSA suppresses oxidative stress by various mechanisms such as the reduction of intracellular ROS and the increase in the expression of phosphorylated-FOXO_1_ and phosphorylated-superoxide dismutase-2 (SOD_2_) [12] (Figure 2). In another study, TSA decreased the levels of MDA and increased the expression of FoxO_3_a, SOD_2_ and catalase (CAT), which may be related to the promotion of the level of H_4_ acetylation of the FoxO_3_a promoter region in H_9_c_2_ rat myocardial cell line injury mediated by oxidative stress [11]. Similarly, Qiu and coworkers [13] investigated the antioxidant effect of TSA against human lens epithelial cells (HLECs) following exposure to ultraviolet-B (UVB) by evaluating the levels of superoxide dismutase (SOD), ROS, MDA, and total antioxidant capacity (T-AOC). Results showed that this compound has significantly elevated the SOD and CAT levels, and reduced MDA and ROS levels, thus protecting HLECs from oxidation. Summarized in Table 1 are the antioxidant activities of trichostatin A.

The antioxidant effects of TSA are mainly related to its capacity to inhibit enzymes involved in ROS generation. Moreover, the modulation of gene expression of proteins controlling ROS production has also been revealed (Table 1). Other suggestions about antioxidant mechanisms of TSA related to the effect of this natural compound on a molecular system involved in oxidative stress generation such as NADPH oxidase, which is mainly implicated in some human cancers.

## 4. Antidiabetic Activity of TSA

**Figure 3:**
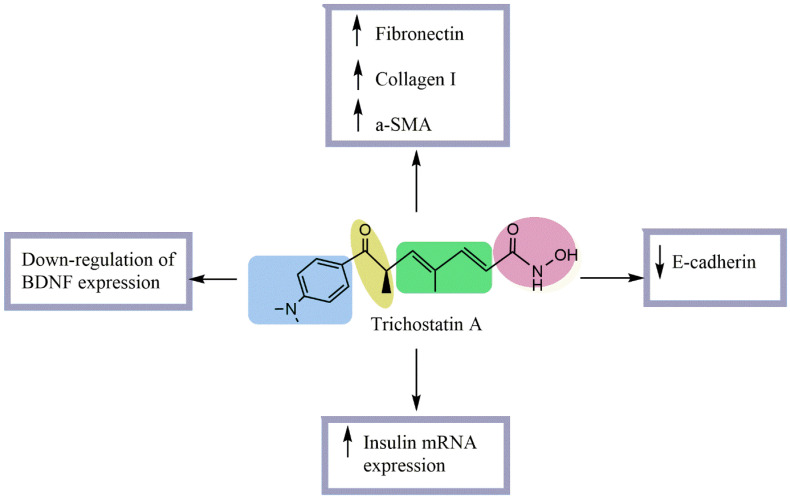
Antidiabetic mechanisms of TSA.

Diabetes is a complex multifactorial disease expressed by a disorder of glucose concentration in the blood. The anti-diabetic effect is expressed by the capacity of anti-diabetic molecules to reduce blood glucose levels, increase insulin secretion, and protect the β-pancreatic cells as well as prevent the complications of diabetes. Within this context, the anti-diabetic effect of TSA has been investigated by several [15,16,17]. Noh et al. [16] investigated the effect of TSA in streptozotocin (STZ)-induced diabetic kidneys. These authors showed that this molecule does not affect blood glucose or kidney/body weight ratio but significantly decreases the urinary excretion of proteins and creatinine. To elucidate the mechanism of an antifibrotic effect of TSA in diabetes, they carried out an *in vitro* study using normal rat kidney tubular epithelial (NRK52-E) cells stimulated with TGF-β1. Results revealed that the TSA prevents the increase in fibronectin, collagen I, and α-SMA and the decrease in E-cadherin expression at both mRNA and protein levels. Another study was conducted to assess the effect of this molecule on insulin secretion from β-cells, GLP-1 secretion from L-cells, and recombinant insulin secretion from engineered L-cells by exposing them to TSA for 24 h (Figure 3). Results indicated that TSA treatment increased the secretion per viable cell in a dose-dependent manner for all cell types in a way that could significantly improve the regulation of blood glucose in diabetes. TSA’s effect on mRNA levels was variable, but it enhanced the intracellular polypeptide secretion [17].

Recently, An and collaborators [15] studied the capacity of TSA to ameliorate the peripheral nerve degeneration of diabetic peripheral neuropathy using cultured rat Schwann cells (RSC96) and streptozotocin (STZ) induced diabetes in rats. They found that treatment with TSA reverses the high glucose condition resulting in the downregulation of BDNF (brain-derived neurotrophic factor) expression in Schwann cells. It also promoted GRP78 expression and the binding ability of GRP78 with BDNF, which mediated TSA-improved BDNF expression in high glucose-cultured Schwann cells. TSA was also involved in UPR (unfolded protein response)-associated transcription factor XBP-1 s and ATF6. Listed in Table 2 are the antidiabetic effects of trichostatin A.

According to different obtained results, TSA can be considered a potent antidiabetic drug with its other molecular actions at subcellular, cellular, and molecular levels. Indeed, with its actions on molecular targets such as the control of mRNA, TSA is among the natural antidiabetic compounds by which novel targeted therapy can be developed for diabetes mellitus.

## 5. Anti-Inflammatory Activity of TSA

**Figure 4:**
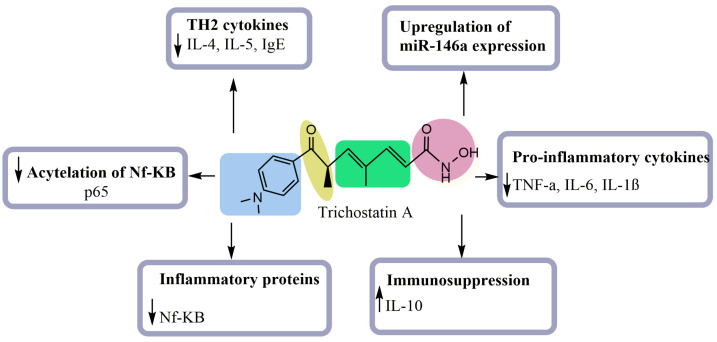
Anti-inflammatory mechanisms of TSA.

The inhibition of inflammation is one of the most important approaches to tackling numerous complicated pathologies such as cancer and atherosclerosis. Several bioactive compounds are well-known for their anti-inflammatory effect. In this regard, numerous studies have dealt with the anti-inflammatory activity of TSA [18,19,20,21,22]. Choi et al. [25] evaluated the anti-inflammatory effect of TSA in a mouse asthma model. Results showed that TSA attenuates the development of allergic airway inflammation by decreasing the expression of CD4+ T cell infiltration cytokines such as IL-4 and IL-5, and IgE (Figure 4). On the other hand, Han and Lee [19] investigated the effect of TSA on lipopolysaccharide-(LPS)-stimulated macrophages using enzyme immunoassay, Western blot analysis and RT-PCR. These researchers found that the pretreatment with TSA significantly reduces levels of mRNA and proteins and decreases the proinflammatory cytokines (TNF-α, IL-6, and IL-1β). This compound also increased the level of the immunosuppressive cytokine IL-10 and reduced the cell surface markers of macrophage maturity (Figure 4).

In another experimental model, TSA enhanced palmitic acid-induced IL-6 production and the expression of inflammatory genes induced by LPS in preadipocytes. It additionally enhanced the expression of inflammatory proteins and NF-κB-dependent transcriptional activity, which might be caused by the increase in the acetylation of NF-κB p65 at lysine 310 and the duration of the nuclear translocation of NF- κB [21]. Similarly, Zhang and colleagues [22] evaluated the LOX inhibitory effect of TSA in phorbol myristate acetate-induced macrophages. Results revealed that TSA exhibits potent anti-inflammatory activity by decreasing the levels of TNF-*α*, IFN-*γ*, IL-10 and IL-18 and increasing the acetylation level of NF-κB and p65. TSA inhibited inflammation by enhancing histone acetylation to selectively suppress the expression of proinflammatory genes (Figure 4). Recently, TSA was evaluated in alveolar macrophages using a lipopolysaccharide (LPS)-induced inflammatory response [20]. The results revealed a potent inflammatory effect, which might be triggered by inhibition of the TNF-α molecule and the upregulation of miR-146a expression. Shown in Table 3 are the anti-inflammatory effects of TSA.

According to the results of various works, it seems that TSA exerts very interesting anti-inflammatory effects with multiple mechanisms. However, the number of works remains limited using less varied experimental approaches. Furthermore, other investigations using other *in vivo* models could give more significant results.

## 6. Anticancer Activity of TSA

### 6.1. Direct Anticancer Mechanisms of TSA

TSA inhibited the growth of different cancer cells via cycle arrest and apoptosis. This compound suppressed the invasion and migration and reduced the radio resistance in many cancer cell lines in a time- and dose-dependent manner. According to the literature, numerous studies investigated the cytotoxicity impact of TSA and the mechanisms by which TSA affects cancer cells. Although the antitumor activity of TSA seems to be strongly linked to its HDAC inhibitory effect, this compound’s antitumor molecular mechanisms are multiple and target different pathways. Table 4 lists the anticancer activity of trichostatin A along with pertinent references.

The mechanisms involved in anticancer effects of TSA are different and related to each type of cancer. These mechanisms are depending to molecular interaction between TSA and main targets of cancer cells. In the following sections, we describe the anticancer effects of TSA against each type of cancer and the mechanisms involved.

#### 6.1.1. Anticancer Action of TSA against Brain Cancer Cells

Research findings showed that TSA inhibits differentiation and proliferation and tumor sphere formation of glioblastoma (GBM) [52,141,142]. In addition, TSA upregulated the expression of numerous tumor suppressor genes through epigenetic modification in GBM [143]. The TSA-induced cell cycle arrest in GBM was associated with the upregulation of p21^WAF1^ and p53, and the downregulation of cell cycle regulators such as cdk4 and 6, and cyclin D1 with the reduction in phosphorylated Rb and Akt [141,142,144,145]. Sassi et al. [52] reported that TSA inhibited the proliferation and colony formation of U87 glioblastoma cells without affecting their viability and migration. Similarly, Hoering et al. [27] showed that TSA induces apoptosis of tumor cells, enhances the sensibility of GBM cells to innate immune responses *in vitro* and delays tumor growth of GBM xenografts *in vivo*. These findings prompted more investigations to involve TSA in improving brain cancer therapy. In this respect, a study on the human malignant glioma cell lines LNT- 229 and LN-308 NMRI nude mice revealed that TSA causes upregulation of natural killer group-2 member-D (NKG2D) ligands and immunogenicity in GBM cells and the retardation of tumor growth of GBM xenografts (*in vivo*) [27].

#### 6.1.2. Anticancer Action of TSA on Neuroblastoma (NB)

In neuroblastoma, TSA inhibited cell viability in neuroblastic-type NB cells by promoting the acetylation of Ku70, a Bax-binding protein, which resulted in Bax release and its activation, and consequently in cell death. TSA-induced cell death in neuroblastoma cells was linked with CREB-binding protein (CBP) expression, where the upregulation of CBP expression interrupted Bax–Ku70 binding in neuroblastic cell types and sensitized them to TSA. At the same time, the down-regulation of CBP enhanced their resistance to TSA [114].

#### 6.1.3. Anticancer Activity of TSA on Human Tongue Squamous Cell Carcinoma

**Figure 5:**
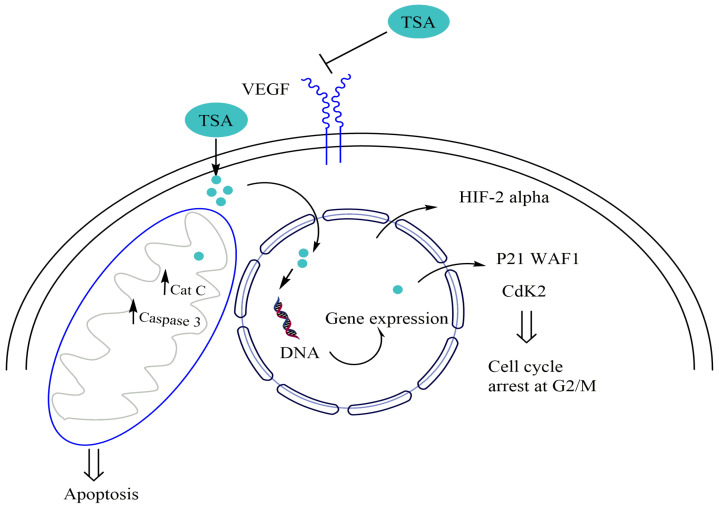
Mechanisms of TSA against brain cancer cells. Abbreviations: CDK: Cyclin Dependent Kinase; HIF Hypoxia Inducible Factor; VEGF: Vascular endothelial growth factor; WAF1: Wild-type P53-activated Fragment 1.

TSA exhibited potent antitumor activity against human tongue squamous cell carcinoma (TSCC) *in vitro* by inducing cell cycle arrest and apoptosis with inhibition of cell proliferation and invasion. This effect was mediated by the downregulation of hypoxia-inducible factor-1α (HIF-1α) protein and vascular endothelial growth factor (VEGF) at the protein and mRNA levels under both normoxic and hypoxic conditions. HIF-1α is a transcription factor able to enhance tumor angiogenesis at a high level via the upregulation of VEGF [122]. TSA suppressed the expression of HIF-2α protein in fibrosarcoma cell line HT1080 via a proteasome-dependent manner [14] (Figure 5).

Ahn et al. [23] revealed the potential value of TSA in inhibiting oral tumor growth by investigating the mechanisms underlying the antitumor activity of TSA on human oral squamous carcinoma cells. TSA inhibited cell proliferation in YD-10B cells as revealed by the MTT assay. TSA also arrested cell cycle progression at the G2/M phase through the upregulation of p21^waf^ expression, downregulation of Cyclin B1, and reduction of the inhibitory phosphorylation of Cdc2 (Figure 5). Additionally, TSA induced mitochondrial membrane destruction after 48 h of treatment. TSA also caused cytochrome *c* release which increased the proteolytic activation of caspase-3 and caspase-7 in YD-10B cells [23].

#### 6.1.4. Anticancer Activity of TSA on Nasopharyngeal Carcinoma

TSA significantly inhibited the proliferation of human nasopharyngeal carcinoma (NPC) cells in poorly differentiated NPC cell line CNE2 and the undifferentiated C666–1 cell. Short-term treatment with TSA inhibited PNC cells by inducing cell cycle arrest with the concomitant upregulation of CDK6 expression and the downregulation of cyclinD1 and E1, CDK2 and 4, p16 and p21 expression in both CNE2 and C666–1 cell line. The short-term TSA treatment induced EMT-like morphological changes without increasing cell invasion ability in NPC cells [110]. To the best of our knowledge, there is no investigation on the long-term effect of TSA on NPC cell invasion and migration. Thus, the study of the mechanism by which TSA can promote cancer cell invasion in nasopharyngeal carcinoma and the combination of TSA with known EMT inhibitors can help to improve the use of TSA in nasopharyngeal carcinoma therapy.

#### 6.1.5. Anticancer Activity of TSA on Lung Cancer Cells

In human lung adenocarcinoma, TSA inhibited cell proliferation and migration with or without TGF-β. TSA acted via the alteration of tight junction proteins that play a key role in the function of the cells. TSA induced the downregulation of claudin-2 (CLDN-2) and the upregulation of angulin-1/LSR along with the induction of cellular metabolism in A549 cells [111]. CLDN-2, upregulated in lung cancer tissues, results in the proliferation of lung cancer cells, while the downregulation of angulin-1/LSR causes malignancy. The anticancer activity of TSA against human lung cancer could be mediated by the upregulation of Krüppel-like factor 4 (klf4) which acts as a tumor-suppressor in the A549 cell line upon TSA treatment [140]. TSA also inhibited radiation-induced EMT and reduced cancer cell migration in the A549 cell line. TSA could reverse radiation-induced lung EMT via the upregulation of E-cadherin expression, which was associated with the inactivation of the signaling molecule, TGFb1, and the decline of Snail and slug transcription factors recognized as enhancers of EMT [95,146].

Choi et al. [25] provided important new insights into the possible molecular mechanisms of the anti-cancer activity of TSA. In a study conducted on a human lung carcinoma cell line A549, treatment with TSA caused inhibition of the viability and induction of apoptosis in a concentration-dependent manner. Apoptosis of the A549 cells was mediated by the alteration in Bcl-2 expression and the activation of caspase-3 and caspase-9 proteases. The anticancer effect of TSA was also associated with the specific inhibition of COX-2 expression and PGE production. In a study conducted on human lung carcinoma cell line A549, TSA treatment caused growth inhibition and morphological changes in a concentration-dependent manner. This effect was accompanied by a marked inhibition of cyclins, positive regulators of cell cycle progression, which was attributed to the control of the overexpression of tumor suppressor p53 and Cdk inhibitors [28].

In the ZAP-Grg1 transgenic mouse line, TSA suppressed lung adenocarcinoma development. This was attributed to the inhibition of ErbB1 and ErbB2 expression and the repression of vascular signaling through VEGF [83]. Kim et al. [78] examined the effects of TSA on osteoclast differentiation induced by the differentiation factor RANKL. These researchers showed that inhibition of HDAC by TSA suppressed osteoclastogenesis *in* *vitro* and *in vivo*. These results implicated c-Fos as an interesting potential target of TSA function and suggested that the *in vivo* TSA can diminish inflammatory bone destruction. In another study, stopping of cell growth and inhibition of colony formation in lung cancer cells were attributed to the increased expression of miR-15a/16-1 caused by TSA treatment, which reduced the expression of an important survival protein named Bcl-2 [57].

Furthermore, TSA showed potent cytotoxic activity against both human small- and non–small-cell lung cancer cells (SCLC and NSCLC). In SCLC, TSA caused morphological differentiation and apoptosis, which was associated with an upregulation of acetylated histone 4, p21 and p27, cleavage of PARP protein and a decline of antiapoptotic protein BCL-2 levels in the DMS53 cell line [103]. While, in NSCLC, TSA induced growth inhibition via apoptosis, with the concomitant enhancement of histone H4 acetylation and p21 expression in four cell lines including Calu-1, NCI-H520, NCI-H23, and NCI-H441 [93]. Thus, TSA showed a potent activity against lung adenocarcinoma *in vitro*, which requires further studies to reveal this compound’s efficiency and safety in vivo.

#### 6.1.6. Gastric Cancer, Colorectal Cancer, and Esophageal Cancer

TSA possesses a significant inhibitory effect against colorectal, gastric, and esophageal cancer cells by inducing apoptosis and cell cycle arrest. Published research indicated that TSA induces cell growth inhibition and apoptosis in colon cancer via epigenetic modification. This effect was linked to the upregulation of p21, p27, and p57 expression with the concomitant suppression of DNMT1 and HDAC1 in colon cancer SW480 cell line [48]. In addition, TSA inhibited colon cancer cells by altering claudin-1, a protein involved in the growth of colon cancer at high levels. Furthermore, TSA decreased the stability and suppressed the expression of claudin-1 mRNA by reducing the binding of HuR and increasing the binding of TTP to the 3′-UTR of claudin-1 in human colon cancer cell lines SW480 and SW620 [109]. It also inhibited the cell invasion and migration of colorectal cancer cells by decreasing vimentin and promoting E-cadherin expression, resulting in reversing EMT in the SW480 cell line. The regulation of E-cadherin and vimentin expression by TSA was suggested to be mediated by the decline of transcription factor Slug [124].

In gastric cancer cells, TSA induced apoptosis and cell cycle arrest, which was associated with a significant decrease in glycoprotein non-metastatic melanoma protein B (GPNMB) expression. GPNMB is highly expressed in gastric cancer tissue compared with normal tissues suggesting that the TSA effect against gastric cancer could be mediated by the downregulation of GPNMB [104]. According to Wang et al. [123], the apoptosis induced by TSA was linked to an increase in the p21, p53, and Bax expression and a decrease in Bcl-2, CDK2, and CyclinD1 expression in the AGS gastric cancer cell line. TSA induced DNA damage in human esophageal cancer cell lines EC109 and KYSE150, with the upregulation of Rad9 gene expression at mRNA and protein levels only in EC109 cells. The knockdown of Rad9 by siRNA increased the DNA damaging effect of TSA [99]. It also significantly inhibited the invasion and metastasis of esophageal squamous cell carcinoma. The anti-invasive effect induced by TSA was mediated by the downregulation of HDAC2, which led to a decline in MMP-2 and MMP-9 expression [123]. These preliminary findings showed that TSA could be involved in treating colorectal, gastric, and esophageal cancers.

Recently, two separable ERK1/2-dependent signaling pathways including an ERK1/2–Slug branch and an ERK1/2-PAI- 1 branches were involved in TSA-induced ESCC cell migration. Both TSA-induced ESCC cell migration branches favored the EMT process, while BRD4 was responsible for two separable ERK1/2-dependent signaling pathways [147]. Liu et al. [82] evaluated the altered expression of genes associated with the cell cycle after TSA treatment. Their results revealed a significant reduction of mini-chromosome maintenance protein-2 (MCM-2) in colon cancer cells that was attributed to the increase in phosphorylated JNK, by TSA treatment resulting in cell growth inhibition and apoptosis [82]. Similarly, Dai et al. (2019) provided further evidence on the cytotoxic mechanism of action of TSA. TSA and the IRE1α/XBP1 pathway in WT HCT116 cells induced ER stress and the ER stress was attenuated by the mutation or silencing of TP53. In addition, these researchers showed that the cell viability was increased and the apoptosis rate was reduced in HCT116 TP53(-/-) cells compared with WT HCT116 cells after TSA treatment. Taken all together, these results revealed that TSA-induced ER stress may occur via a p53-dependent mechanism in colon cancer cells, and induction of apoptosis via p53 signaling pathway activation was supported by other studies [148].

In a recent study, An et al. [15] investigated the antitumor activity and the mechanism of modulating gastric cancer cell growth by TSA. They showed an antiproliferative effect of TSA against MKN-45 and SGC-7901 cells, which significantly suppressed the number and size of colonies. Moreover, flow cytometry methods revealed that TSA induces G1 phase cell cycle arrest and apoptosis and affected the expression of related factors in mitochondrial apoptotic signaling and cell cycle-related regulatory pathways. In addition, TSA increased the acetylation of histone H3K27 and downregulated the expression of PI3K and p-AKT. In a similar fashion, Han and coworkers [149] reported that TSA treatment induces mesenchymal-like morphological modifications BGC-823 human gastric cancer and increases the expression rate of the mesenchymal markers vimentin and twist. It also suppressed cancer cell colony formation in both cell lines and led to the deregulation of the critical signaling molecule involved in EMT named *β*-catenin.

#### 6.1.7. Anticancer Effect of TSA on Hepatocellular Carcinoma

TSA can inhibit hepatocellular carcinoma through mitochondrial and cytoplasmic apoptotic pathways in HCCLM3, MHCC97H, and MHCC97L cell lines, with no effect on primary hepatocytes. It altered the expression of many genes involved in cell growth, differentiation, and apoptosis such as Bax, Bak, Bim, p53, and p73 that were up-regulated by TSA. At the same time, Bcl-2, Bcl-xL, Mcl-1 were downregulated with TSA treatment [47,100]. In addition, TSA inhibited the cell growth and induced apoptosis in hepatocellular carcinoma via the re-activation of ERα gene expression in the Hepa1-6 cell line [47]. In human hepatoma cell lines including HuH7, Hep3B, HepG2, and PLC/PRF/5 cells, TSA inhibited cell growth in a concentration-dependent and time-dependent manner against all studied cell lines. Moreover, the results revealed that TSA suppresses cell growth, induces apoptosis, and inhibits specific genes expression in hepatoma cell lines [60].

#### 6.1.8. Anticancer Effect of TSA on Pancreatic Adenocarcinoma

Research findings showed that TSA can act against human pancreatic cancer cell lines by enhancing the expression of pro-apoptotic genes such as the BIM gene, and suppressing the expression of anti-apoptotic genes like Bcl-XL and Bcl-W [91]. In this respect, Zhang et al. [134] indicated that the apoptotic effect induced by TSA was linked to the alteration of microRNAs expression in BxPC-3 human pancreatic cancer cells inhibiting their proliferation [134]. To explore the potential effects of TSA against pancreatic cancer (PC) cells, Emonds et al. [30] demonstrated that the cytotoxic effect of TSA in PC cells is associated with the increase of acetyl-H3, p21Waf1, phospho-p38 and Bax levels, and the decrease of phospho- ERK 1/2 and phospho-AKT. Similarly, Chen et al. [58]. showed that TSA can induce apoptosis of pancreatic cancer cells. TSA treatment significantly increased the expression levels of Bax and caspase-3 resulting in reduced survivin and anti-apoptotic Bcl-2 [58].

#### 6.1.9. Anticancer Effect of TSA on Leukemia

TSA exhibited considerable anti-leukemic effects by regulating the cell cycle and inducing apoptosis in numerous human leukemic cell lines, with low toxicity against normal peripheral blood mononuclear cells (NPBMNC) [53,107,115]. It caused the growth inhibition and morphological changes in a human leukemic cell by inhibiting cyclins and the proliferating cell nuclear antigen (PCNA), positive regulators of cell cycle progression, and by the upregulation of Cdk inhibitors such as p16, p21 and p27 [126]. It additionally showed potent activity against chronic lymphocytic leukemia (CLL) cells through caspase-dependent inhibition of Wnt [102]. Wnt is highly expressed in CLL and is involved in developing several types of cancer. TSA treatment led to the upregulation of caspases-3 and 7 activity and DKK1 mRNA expression known as a negative regulator for the Wnt. TSA inhibited CLL cells via the modulation of histone acetylation by inhibiting H4 histone deacetylation [102]. In TK6 human B lymphoblastoid, TSA induced G1 cell cycle arrest and apoptosis in a caspase 3–independent apoptotic pathway. Furthermore, the cytotoxicity effect of TSA in TK6 cells might be linked to the ability of this compound to cause DNA and chromosome damage through chromosome breakage and by increasing the levels of aneuploidy [98].

In addition to its potent antitumor effects, numerous studies showed that TSA exhibits an immune-regulatory effect. TSA suppressed the expression of the interleukin-2 gene in CD4+T-cells [92]. It also reduced the production of the pro-inflammatory cytokines (TNF-α and IL-6) and type I interferon (IFN-I) known for their role in autoimmune diseases [105]. TSA played an important role in the control of allergic airway inflammation mediated by Group 2 innate lymphoid cells (ILC2) activation, and downregulated the number of ILC2 expressing IL-5 and IL-13 cytokines and consequently attenuated lung eosinophilia and mucus hypersecretion in a mouse model [119].

TSA also affected cell cycle progression in Epstein-Barr virus (EBV)-transformed B lymphoblastoid cell lines LCLs (SNU-20 and SNU-1103). EBV is a ubiquitous human herpes virus recognized as being instrumental in causing human malignancies of lymphoid [150]. In this regard, the induction of EBV lytic antigens is one of the strategies used to treat EBV-associated malignancies. At this level, TSA induced EBV lytic replication by upregulating EBV lytic genes expression (BZLF1, BMRF1, gp110, BRLF1, BALF5, BBLF2/3 and gp350) in lymphoblastoid cell lines [107]. These important results show that TSA exhibits a cytotoxic effect and immunomodulatory potential suggesting this compound’s use in the therapy of leukemia and the treatment of autoimmune and inflammatory diseases. The anti-proliferative effects of TSA were also reported in murine pro-B lymphoma cell line FL5.12. TSA exerted a potential inhibition of the proliferation of FL5.12 cells in a time and dose-dependent manner. TSA treatment led to DNA fragmentation and the activation of caspase-3 and PARP, resulting in the release of apoptotic protein Bim [80]. Anti-Molt-4 cell activity of TSA was also reported in a time- and dose-dependent manner by Hong et al. [31]. These authors concluded that the TSA growth-inhibition effect could be attributed to its apoptosis-inducing effect on Molt-4 cells [31].

#### 6.1.10. Anticancer Effect of TSA on Osteosarcoma/Excessive Bone Resorption

Findings showed that TSA inhibits tumor growth in MG-63 osteosarcoma cells *in vitro* and *in vivo*. This antitumor effect upon TSA treatment is partially mediated by the suppression of HIF-2α protein expression. Thus, TSA could be used to treat excessive bone resorption by inducing osteoclast apoptosis via the upregulation of p21^WAF1^. Osteoclasts can cause many pathologic conditions such as osteoporosis and tumor-induced bone destruction [131]. Deng et al. [26] suggested that TSA significantly inhibits cell growth and promotes apoptosis in a dose-dependent manner through p53 signaling pathway activation in MG63 cells [26]. Moreover, TSA was tested in wild-type p53 (HT116 cells) and mutant p53 (HT29 cells) colorectal cancer cell lines. Results showed that it induces apoptosis, enhances the expression of Bax, lowers the levels of Bcl-xl and Bcl-2, and induces cell cycle arrest in the G2/M phase [89]. In addition, TSA induced apoptosis and cell cycle arrest in the G2/M phase in colorectal cancer cells via p53-dependent and -independent pathways.

TSA also inhibits proliferation, induces apoptosis, inhibits the invasiveness of osteosarcoma cells in vitro, and arrests the cell cycle in the G1/G2 phase in Osteosarcoma cells [59]. It also had a role in inducing autophagy in human osteosarcoma (U2OS) cells through the rapamycin (mTOR) signaling pathway and enhances forkhead box O1 (FOXO1) transcriptional activity [34]. Using immunofluorescence, immunoprecipitation, Western blots, and qPCR, Geng et al. [50] studied the cell proliferation, cell cycle, survival mechanisms, the localization and post-transcriptional modification of GLI1 protein, the target gene P21 of RPMI8226, and MM.1S cells treated with TSA. TSA exerted a time and dose-dependent MM cell growth repression, and induced cell apoptosis via the abrogation of hedgehog signaling. TSA treatment exhibited versatile effects on the hedgehog transcriptional factor GLI1. The proteasome-dependent degradation of GLI1 was promoted by TSA-mediated acetylation, and TSA-induced p21 upregulation damages the transcription of GLI1 [50].

#### 6.1.11. Anticancer Action of TSA on Musculoskeletal Sarcomas (Rhabdomyosarcoma)

In rhabdomyosarcoma (RMS), a common type of musculoskeletal sarcomas, TSA inhibited cell proliferation and reversed RMS malignancies behavior by reactivating its differentiation to the original skeletal muscle type. The activity of TSA in RMS cells was suggested to be mediated by the modification of small miRNAs expression, notably by the upregulating of miR-27b, which is involved in the process of myogenesis [117].

#### 6.1.12. Anticancer Action of TSA on Mast Cell Tumor (MCT) and Breast Cancer Cells

TSA induced apoptosis in mastocytoma cells in histone acetylation- and mitochondria-dependent pathways. It increased the acetylated histones, H2A, H2B, H3 and H4, activated caspase-3, induced cleavage of poly (ADP-ribose) polymerase (PARP), and decreased Bcl-2, Bcl-xL levels in mastocytoma cell line p815 resulting in a reduction of their mitochondrial membrane potential. TSA treatment showed a reduction in number of viable cells and induced cell death by apoptosis in the MCT of canine [94].

Several studies confirmed the potent antitumor activity of TSA against breast cancer *in vitro* and *in vivo* in a dose-dependent manner through different pathways. TSA-induced apoptosis and cell cycle arrest at the G2/M in both estrogen receptor (ER)-positive and ER-negative breast cancer cells *in vitro* and *in vivo*, with no toxic effect on the normal cells such as MCF-10A [116,118,120,121]. ERα played an essential role in the proliferation of ER-positive breast cancer. Interestingly, TSA was revealed as a potent ERα inhibitor. Therefore, breast cancer cells expressing ER were found to be more sensitive to TSA than the ER-negative cell lines [121]. In ER-positive cell lines such as MCF-7 and T47-D cells, TSA induced a long-term degradation of cyclin A, a decrease of Bcl-2 and myc-c, and a proteasome-dependent loss of ERα and cyclin D1, which led to the re-expression of p21^WAF1/CIP1^ (cell cycle inhibitor) and RhoB GTPase (tumor suppressor) [120,139]. These findings indicate that TSA could be used in the endocrine therapy of breast cancer. In the ER-negative cells like MDA-MB-231 and SKBr-3 cell lines, TSA enhanced the expression of ERα mRNA and p21^WAF1/CIP1^ at the protein level, and declined cyclin A, with the ability to cause cleavage of polyADP-ribose polymerase (PARP) [120]. Noh et al. [97] showed that TSA lowered the ERα protein expression under both normoxia and hypoxia via a proteasome-mediated pathway and enhanced the downregulation of *ESR1* transcription under hypoxia conditions [97].

In the H-ras-transformed human breast epithelial (MCF10A-ras) cells, TSA induced morphological changes and cell cycle arrest by increasing the expression of p53 and p21^WAF1/Cip1^ and activating ERK1/2. The cytotoxic effect of TSA against MCF10A-ras cells was partly linked in par to its ability to decrease phosphorylated Rb expression [151]. The inhibition of breast cancer cells with TSA treatment depends on the mitochondrial-mediated ROS by suppressing the activities of mitochondrial complexes I and III. Unfortunately, this pathway could be blocked by antioxidants such as GSH, NAC, and vitamin C [116]. On the other hand, TSA could also initiate cell cycle arrest and apoptosis via the 15-Lox-1/13(S)-HODE pathway by the induction of 15-Lox-1 activity associated with the elevation of 13(S)-HODE (15-Lox-1 metabolite) [118]. Furthermore, aberrations of microRNAs expression were confirmed to have a noticeable effect on cancer development [152]. In this respect, TSA modified the expression of microRNAs in the apoptosis-resistant MCF-7TN-R cell line. Additionally, TSA significantly upregulated the expression of many microRNAs considered tumor suppressors or involved in anti-migration, cell cycle arrest, anti-metastatic, and anti-EMT effects. TSA decreased the expression of microRNAs characterized as oncomiRs, which are involved in tumorigenesis [29].

Moreover, TSA was found to suppress the invasion and migration of MCF-7 cells by reversing EMT via knockdown of zinc finger protein SNAI2 (SLUG), which led to the upregulation of E-cadherin and downregulation of vimentin expression [153]. TSA showed great antitumor activity *in vivo* in breast cancer xenografts and carcinogen-induced rat mammary cancer models [120,121]. All these results provided a rationale for the important role of TSA in treating breast cancer due to its ability to induce cell growth inhibition and metastasis in different breast cancer types *in vitro* and *in vivo*. In 2006, Alao et al. [24] investigated the role of GSK3β in mediating the cytotoxic activities in MCF-7 breast cancer cells treated with TSA. These researchers showed that TSA induces Akt dephosphorylation in a PP1-dependent manner resulting in the activation of GSK3β in cells tested. These findings identified GSK3β as an interesting mediator of TSA-induced cytotoxicity in MCF-7 breast cancer cells [24]. In other research work, TSA was tested in T-lymphoblastic leukemia cell line Molt-4 cells. A time- and dose-dependent manner of proliferation inhibition of Molt-4 cells was observed. The application of TSA at different doses (from 50 to 400 μg/L for 24 h) decreased the percentage of G0/G1 cells and arrested cells in the G2/M phase by reducing the expression of HDAC8 in Molt-4 cells [66].

To explain the effects of TSA on epithelial-mesenchymal transition (EMT) in human cancer cells, Han et al. [149] reported that TSA treatment induces mesenchymal-like morphological modifications in MCF-7 breast cancer cells and BGC-823 human gastric cancer, increases the expression rate of the mesenchymal markers vimentin and twist, and suppresses cancer cell colony formation in both cell lines which led to the dysregulation of the critical signaling molecule involved in EMT named of *β*-catenin. In another study, Gao and coworkers [35] investigated the antitumor effect of TSA in H-ras-transformed human breast epithelial cells (MCF10A-ras cells) through a FOXO1-dependent pathway. TSA exhibited antitumoral activity in MCF10A. This antiproliferative effect was attributed to the induction of apoptosis in MCF10A-ras cells and the activation of FOXO1 via P21 upregulation, whereas the knockdown of FOXO1 reduced TSA-induced cell death. TSA induced autophagy in MCF10A-ras cells by blocking the mTOR pathway [35]. Befor this, Alao et al. [51] investigated the mechanisms underlying the antiproliferative effect of TSA. It caused G1-S-phase ER alpha-positive MCF-7 cell cycle arrest because TSA repressed ER alpha and cyclin D1 transcription and induced ubiquitin-dependent proteasomal degradation of cyclin D1. However, in the ER alpha-negative MDA-MB-231 cell line, cyclin D1 degradation was enhanced but its transcription was unaltered by TSA, resulting in cell cycle arrest in the G2-M phase [51]. Skp2/p45, a regulatory component of the Skp1/Cullin/F-box complex is involved in cyclin D1 degradation, which causes the silencing of SKP2 gene expression by RNA interference stabilizing cyclin D1 and abrogated the cyclin D1 downregulation response to TSA [51]. Similarly, TSA demonstrated anticancer potential by inducing apoptosis in MCF10A-ras cells via the activation of FOXO1, and the regulation of autophagy by blocking the mTOR signaling pathway [35].

#### 6.1.13. Anticancer Action of TSA on Endometriosis/Cervical Cancer Cells

TSA showed interesting results in the treatment of endometriosis. It significantly inhibited the proliferation in endometrial stromal cells, associated with the upregulation of PR-B and AR [127]. It additionally exhibited potent activity against cervical cancer cells [128,129,135]. TSA induced a delay in G2/M transition, the formation of defective mitotic spindles and misaligned chromosomes in a transcription-dependent manner by the downregulation of cell cycle regulators cyclin B1, Plk1 and survivin, and upregulation of p53, p21^Waf1^, and p27^Kipl^ in HeLa cell line [96,129]. Similarly, it inhibited the growth of HeLa cells via Bcl-2, oxidative stress- and caspase-dependent apoptosis, which was correlated with an increase in O_2_^•−^ level, GSH depletion, the collapse of mitochondrial membrane potential (MMP), and a decrease of Bcl-2 protein and caspase-3 activation [130,132]. TSA also suppressed the expression of the ubiquitin-specific protease 22 (USP22), a gene overexpressed in most cancer cells and implicated in tumorigenesis [154]. The inhibition of cell proliferation by TSA was attributed to the ability of this compound to affect the physical and chemical nature of cytoskeletons, including microfilaments and microtubules, which play an important role in the maintenance of the cell morphology and material transportation in HeLa cells [137]. The cytotoxic effect of TSA was inhibited under the co-treatment of HeLa cells with the F-actin depolymerizers cytochalasin D (CytoD) and latrunculin B (LatB), indicating that TSA-induced apoptosis might require a dynamic rearrangement of F-actin [130]. It also suppressed rather than enhanced the radiosensitivity of HeLa cells at low doses [155].

On the other hand, Li et al. [79] studied the exact mechanism by which HDAC inhibitors induce p21^WAF1/CIP1^ in HeLa cells. This team observed that TSA, an HDAC inhibitor, induced p21^WAF1/CIP1^ expression in human cervical cancer (HeLa) cells, which is associated with the downregulation of c myc expression [79]. Interestingly, deeper insights were provided by Liu and colleagues [84] about the effects of TSA on cervical cancer development. The results showed that TSA suppressed the proliferation and induced apoptosis and autophagy in cervical cancer cells via the PRMT5/ STC1/TRPV6/JNK axis. TSA also reduced cervical tumor growth in mice xenograft models by the downregulation of osteopontin (OPN) gene expression. OPN is a secreted glycoprotein associated with tumor formation and metastasis. The gene expression of OPN was induced by PMA via the AP-1 transcription factor that forms a heterodimer of c-jun and c-fos at the OPN promoter. TSA suppressed the PMA-induced OPN gene expression by inhibiting c-Jun expression at the protein and RNA levels. Similarly, TSA inhibited the PMA-induced hyperacetylation of histones H3 and H4 associated with OPN promoter [108]. The results of the *in vitro* and *in vivo* studies revealed the high antitumor potential of TSA against cervical cell cancer and consequently the important role that TSA could play in treating this kind of cancer.

#### 6.1.14. Anticancer Action of TSA on Ovarian Cancer Cells

Research findings revealed that TSA inhibits ovarian cancer cells by affecting their morphology and proliferation. This effect seemed to be linked to the ability of TSA to change the expression of p21, Rb, and Id1, which are involved in cell cycle control and differentiation in A2780 ovarian cancer cells [113]. Moreover, TSA induced apoptosis in ovarian carcinoma cells via a mitochondria-dependent pathway by increasing the protein expression of cytochrome *c* and P53 and the activity of caspase-3, -8 and -9 in OVCAR-3 cells [136]. The anticancer activity of TSA against ovarian carcinoma cells could be mediated by the upregulation of Krüppel-like factor 4 (klf4) which plays a tumor-suppressor role in the SKOV3 cell line upon TSA treatment [140], and the apoptotic effect induced by TSA was enhanced by the inhibition of Akt [136].

#### 6.1.15. Anticancer Action of TSA on Urinary Bladder Cancer Cells

TSA exhibited antitumor activity against urinary bladder cancer cells. Bladder cancer is among the most expensive human cancer to treat with a high death rate. A preliminary study showed that the growth inhibition and apoptosis induced by TSA in urinary bladder cancer cells are correlated with a decrease of cyclin D1 and an increase in p21 expression, induction of PARP cleavage, mitochondrial membrane potential loss, inhibition of pAkt, and a decline of Sp1 and surviving [125]. However, the implication of TSA in the prevention and therapy of bladder cancer requires more investigations at different levels. A time and dose-dependent significant antiproliferative effect were reported in the human bladder cancer cell line, BIU-87, via the cell cycle arrest at the G1 phase and induction of apoptotic cell death was correlated with the increase of p21^WAF1^ mRNA expression [79].

#### 6.1.16. Anticancer Action of TSA on Prostate Cancer/Spermatogenesis

TSA increased the frequency of genetic recombination of spermatocyte meiosis and reduced testicular weight and sperm density in male mice [112]. It exhibited a noticeable antitumor activity against human prostate epithelial cells and induced cell growth inhibition and apoptosis in 267B1 cells *in vitro* via caspase-independent pathway and the activation of transcription factor nuclear factor kappa B (NF−κB) [151]. In the DU145 prostate cancer cell line, TSA treatment induced morphological changes and cell cycle arrest, inhibiting survivin expression and enhancing P21 protein expression [133]. However, conflicting results concerning the effect of TSA on cell migration were reported for prostate cancer. Wang et al. [124] revealed that TSA inhibits the cell migration of prostate cancer cells by reducing the expression of the transcription factor, Slug, which suppresses vimentin and enhances E-cadherin reversing EMT in the PC3 cell line [124]. However, Kong and coworkers [156] found that TSA induces EMT in prostate cancer cells and changes their cellular morphology. TSA-induced EMT was accompanied by the enhancement of vimentin, N-cadherin and fibronectin (mesenchymal markers), and the expression of ZEB1, ZEB2, and Slug (transcription factors), which correlated with an increase in tumor virulence. In-depth, experiments are required to determine the safe use of TSA in prostate cancer therapy focusing on the metastasis side.

All related studies showed the potent anti-tumor effect of the TSA. It targeted several kinds of cancer and was involved in treating autoimmune and inflammatory diseases. It inhibited a wide range of cancer cells through different pathways without affecting normal cells. The main mechanism by which TSA protected non-cancer cells from apoptosis was via the activation of ERK1/2 (extracellular signal-regulated kinase) [135]. In addition, no toxicity was observed when high doses of up to 5 mg/kg of TSA were used, suggesting an added value to this compound [121]. However, *in vivo* experiments indicated that TSA is rapidly metabolized, complicating the clinical trials of this important compound and its use in cancer therapy. To overcome this problem, long-term treatment with a high dose of TSA, 500 mg/kg daily for 4 weeks by subcutaneous injection, showed great antitumor activity in the carcinogen-induced rat mammary cancer model [121]. In addition, efforts were made to develop formulations, that protect TSA molecules from inactivation. In this respect, TSA-loaded liposomes (TSA-lipo) formulations significantly protected TSA from inactivation. Subsequently, they resulted in potent growth inhibition of both ER-positive and–negative BC cells *in vivo* at low TSA concentration (1.5 mg/kg/week) by intravenous injection. The anticancer effect of TSA-lipo was characterized by the inhibition of Ki-67 labeling, inhibition of tumor vasculature, and an increase of p21^WAF1/CIP1^ in MCF-7 and MDA cells xenografts [120]. On the other hand, several problems related to the possibility of TSA inducing EMT and enhancing cell cancer migration still need to be carefully addressed and clarified. Therefore, the safe use of TSA in cancer therapy requires further investigations to determine the specific conditions TSA could induce rather than reverse EMT in cancer and consequently increase cell cancer invasion and migration. Also, combinatorial treatment of TSA with a potent EMT-inhibitor could help avoid EMT induction and metastasis upon TSA treatment. Further studies are needed to show the antitumor activity of TSA *in vivo* in numerous cancer types.

### 6.2. Anticancer Activity of TSA through Sensitization

Considering the role of various anticancer drugs in chemotherapy and the emergence of chemoresistance, the effect of TSA on the chemosensitivity of several anticancer drugs in cancer cells was investigated by numerous research groups. Findings emphasized that TSA is a potent chemo-sensitizer in human cancer cells to improve chemosensitivity towards many drugs including cisplatin, valproic acid, etoposide, tamoxifen, gemcitabine, 5-fluorouracil, oxaliplatin, irinotecan and gefitinib, sunitinib, and TRAIL. It also enhances the radiosensitivity of cancer cells. Moreover, many molecules, such as genistein, quercetin, and glycyrrhetinic acid potentiated TSA’s anticancer activity against different cancer cell lines. Along this line, TSA reestablished cisplatin sensitivity in many cisplatin-resistant cancer cells and augmented cisplatin activity by eliciting cisplatin-induced apoptosis via various mechanisms. In the head and neck squamous cell carcinoma cell line (UT-SCC-77), cisplatin-induced apoptosis was enhanced by TSA pretreatment [36]. TSA decreased lysosomal pH, which augmented cathepsin activity resulting in reduced LAMP-2 level, and the potential LMP promotion. Cells lacking LAMP-2 became more sensitive to cisplatin-induced apoptosis. Earlier work indicated that a lower lysosomal pH increases the efficiency of cisplatin-induced apoptosis. It reduces lysosomal pH and elicits lysosomal proteases and sensitized cells to cisplatin [36].

Numerous studies showed that the resistance of ovarian cancer cells to the proapoptotic effects of chemotherapy is partly due to the deficiency in Apaf-1 activity. Tan et al. [157], while evaluating Apaf-1 function showed that TSA restores Apaf-1 function in chemoresistant ovarian cancer cells, sensitizing them to cisplatin-induced apoptosis via the activation/cleavage of procaspase-9. Cisplatin stimulated mitochondrial release of cytochrome *c*, which was then complexed with Apaf-1. The latter bound and activated caspases-3 and -9, leading to apoptosis [157]. Recently, Lambert et al. [39] reported that TSA reduces cell viability in cisplatin-sensitive (A2780WT) and reestablishes cisplatin sensitivity in human ovarian cancer cells cisplatin-resistant (A2780RES) by eliciting cisplatin-induced apoptosis (boosted caspase-9 activity), autophagy (increased LC3-II expression), and cell cycle arrest (increased p21 expression). TSA additionally reduced taurine transporter (TauT) expression/activity in A2780RES cells to values similar to A2780WT cells suggesting a synergistic activation of apoptosis /autophagy and reduced TauT activity rather than facilitating cisplatin uptake or increasing cisplatin-induced DNA damage. TSA synergistically enhanced the antitumor effect of cisplatin and re-sensitized cisplatin-resistant bladder cancer cells leading to 90% death of T24R2 cells [41]. It was suggested that the potent synergistic effect could involve cell cycle arrest by potentiating cisplatin-induced S and G2/M phase cell cycle arrest and the induction of caspase-mediated apoptosis and/or the upregulation of the expression of proapoptotic proteins, Bad and Bax.

In human lung adenocarcinoma cell line A549 and CDDP-resistant derivative (A549/CDDP), Wu and colleagues [158] showed that a low concentration of TSA sensitized cisplatin-resistant apoptosis. TSA upregulated pro-apoptotic proteins (death-associated protein kinase (DAPK)) mediating A549/CDDP cell death induced by cisplatin. TSA pretreatment induced elevation of the active form of DAPK in A549/CDDP, which elicited the chemosensitivity of cells to cisplatin. Similarly, co-treatment of human urothelial carcinoma (UC) cell lines (NTUB1 and T24) with TSA and three chemotherapeutic agents (cisplatin, gemcitabine, and doxorubicin) induced synergistic cytotoxicity and significantly potentiated apoptosis. The combination acted by suppressing Raf/MEK/ERK pathway as it is involved in many aspects of tumorigenesis, including cell growth, proliferation, survival, apoptosis, and chemoresistance in UC [159]. The activated Raf/MEK/ERK pathway was observed in human bladder UC specimens from patients with chemoresistant status. The co-treatment with TSA increased cleaved caspases-3,-7, and PARP compared with those induced by chemotherapeutic agents alone, and also suppressed the chemotherapy-induced activation of phospho-Bcl2, an anti-apoptosis regulator. The same conclusions were confirmed, *in vivo,* in a xenograft nude mouse model.

Several studies reported that hepatitis B virus X protein (HBx) exerts anti-apoptotic effects leading to a potent chemoresistance effect in hepatocellular carcinoma (HCC) cells. In this respect, Zhang et al. [135] showed that the pretreatment with TSA and etoposide could significantly overcome the increased resistance of HBx-expressing HCC cells to chemotherapy. This combination significantly sensitized HBx-expressing liver cancer cells to etoposide treatment via the induction of apoptosis by inhibiting ERK phosphorylation, reactivating caspases and PARP, and inducing translocation of p53 and Bid to the cytoplasm [135]. In another study, the co-treatment of drug-resistant non-small cell lung carcinomas (NSCLC) (H157, H23 and H1299) with etoposide and TSA induced apoptotic through caspase-dependent pathway accompanied by a significant decrease in Bcl-xL expression allowing Bax activation. A subsequent initiation of the apoptosis, inducing factor (AIF)-dependent death pathway in H157 cells was also observed [160]. Moreover, in human leukemia cell lines (HL60 and U937 cells), the use of TSA and valproic acid (VPA) potentiated etoposide-induced cytotoxicity and apoptosis, which was associated with the activation of caspases and the loss of mitochondrial membrane potential [161].

TNF-related apoptosis-inducing ligand (TRAIL) is a potent anti-cancer agent due to its high selectivity in eradicating cancer cells while sparing normal cells. However, different cancer cells showed TRAIL resistance. In this context, numerous studies reported that TSA enhances TRAIL efficacy and re-sensitizes various cancer cells resistant even at high doses of TRAIL. Researchers [162] showed that low doses of TSA sensitized MM1S myeloma cells were resistant to TRAIL-induced apoptosis and enhanced TRAIL cytotoxicity through the caspase-independent pathway. It induced apoptosis involving the downregulation of the antiapoptotic Bcl-2 proteins, Bcl-2 and Bcl-X_L_, without altering FLIPS expression. The expression of Bcl-2 members (Bim and Bid) was also upregulated while the expression of PUMA (a and b), Bax, and Noxa, was down-regulated. TSA also induced the transcription of TRAIL death receptor DR5. In another study, Kong et al. [156] showed that zebularine and TSA with TRAIL (TZT) treatment sensitizes human breast adenocarcinoma cells (MDA-MB-231 and MCF10A) and augments apoptosis as compared with TRAIL alone. Apoptotic features, including morphological changes, apoptotic activity, and the expression of cleaved poly (ADP) ribose polymerase (PARP) protein were more prominent in MDAMB-231 as compared to MCF10A. No changes in cell cycle were recorded in MDA-MB-231 cells under TRAIL and TZT treatments suggesting other mechanisms [156]. Similarly, researchers showed that the co-treatment of human TRAIL-resistant ovarian cancer cells (SKOV3 and Hey8), with TSA and TRAIL inhibits cell proliferation and sensitizes them to TRAIL-induced apoptosis through caspase-dependent mitochondrial pathways [101]. Moreover, treating SKOV3 cells with TSA and TRAIL significantly accelerated caspase-8 and truncated Bid resulting in the cytosolic accumulation of cytochrome *c* and the activation of caspases-3 and -9. On the other hand, the cleavage of PARP, an endogenous substrate of caspase-3, and the upregulation of Bax led to a significant loss of Bcl-2 and Bcl-xL. The sensitization was associated with the downregulation of c-FLIP_L_ via the inhibition of the EGFR pathway, involving caspase-dependent mitochondrial apoptosis as TRAIL alone did not alter the protein level of c-FLIP.

In gastric cancer cell lines (AGS, NCI-N87, SNU-1 and SNU-16), TSA potentiated TRAIL-induced apoptosis in caspase-dependent manner via the inhibition of the ERK/FOXM1 pathway [80]. The combination rendered gastric cancer cells more vulnerable to TRAIL-mediated cytotoxicity and suppressed cell viability in TRAIL-resistant cell AGS and SGC-7901. In the absence of TSA, slight activation of caspases-3, -7, -8, -9, and PARP was observed, whereas the cotreatment greatly potentiated these effects in both SGC-7901 cells. TSA also contributed to the upregulation of DR5 and downregulation of antiapoptotic proteins (XIAP, Mcl-1, Bcl-2 and Survivin) that could be regulated by oncogenic transcription factor Forkhead boxM1 (FOXM1). TSA treatment inhibited FOXM1 expression at both the transcription and protein levels. The expression level of FOXM1 showed a negative correlation with TRAIL sensitivity. FOXM1 downregulation could be ascribed to the inactivation of the ERK pathway, which sensitizes cells to TRAIL.

Research findings [40] showed that TSA acts as a sensitizer in chemotherapy and enhances the response to chemotherapeutic agents (gemcitabine, 5-fluorouracil, oxaliplatin, irinotecan and gefitinib) in inhibiting ten pancreatic adenocarcinoma cell proliferation. Ten human pancreatic cancer cell lines, seven derived from primary cancer (MiaPaca2, PaCa3, PaCa44, Panc1, PT45P1, PSN1, and PC) and three from metastatic cancers (HPAF II, CFPAC1, and T3M4) were investigated. TSA was the best partner for all drugs except for 5-fluorouracil leading to potent inhibition of cell growth. The combination of TSA and irinotecan exhibited potent growth inhibition (80%) in most cell lines. In a similar fashion, Zhang et al. [163] showed that TSA increases the chemosensitivity of anticancer drugs in two human gastric cancer cell lines (OCUM-8 and MKN-74). The combination of TSA with five anticancer drugs, namely 5-fluorouracil (5-FU), paclitaxel (PTX), oxaliplatin (OXA), irinotecan (SN38), and gemcitabine (GEM) caused a synergistic anti-proliferative effect by combining TSA (30 ng/mL) with 5-fluorouracil, paclitaxel, and irinotecan [163]. These three anticancer drugs target cancer through different mechanisms and are used clinically. Furthermore, TSA upregulated the expression of p21, p53, DAPK-1, and the DAPK-2 gene in both OCUM-8 and MKN-74 cells which could be involved in the synergistic effect. The expression level of caspase-3 mRNA increased in OCUM-8 but not in MKN-74, suggesting a key role of caspase-3 in chemosensitivity induced by TSA. It was suggested that the bcl-2 family might not contribute to the enhanced chemosensitivity of TSA as no alteration of bcl-2 was observed.

TSA sensitized estrogen receptor (ER) α-negative in formerly antihormone-unresponsive human breast cancer cells (MDA-MB-231, Hs578T and ZR75-1) to tamoxifen treatment possibly by upregulating ER β activity [164]. TSA enhanced the ER transcriptional activity as visualized by estrogen response element-regulated reporter and progesterone receptor expression. It seems that the high ER transcriptional activity is mediated by ER β rather than α as TSA induced the expression and nuclear translocation of ER β but not α. Sato et al. [165] showed that the combination of TSA-Sunitinib is effective against RCC cells 786-O, ACHN, and Caki-1 RCC cell lines, especially in 786-O, by enhancing apoptosis or growth inhibition through an increase of p21. VEGF protein expression was suppressed by the used combination. Flow cytometry revealed that the apoptotic cell population (sub-G1) was significantly higher in the TSA-Sunitinib combination group compared to the single SU treatment group. In ACHN cells, a cell cycle arrest at the S and G2/M phase was observed in the combined treatment group. Additionally, p21 was significantly increased in both 786-O and ACHN cells.

In renal cell carcinoma (786-O, ACHN, and Caki-1 RCC cells), TSA reduced sunitinib resistance by triggering intracellular metabolome shifts [166]. Combined metabolome and transcriptome analysis suggested that TSA affects the energy productive metabolic pathways, such as those involving the TCA cycle and nucleotide metabolism. The combination of sunitinib and TSA increased cell death with PARP cleavage, an early marker of mitochondrial apoptosis. In contrast, the receptor tyrosine kinase signaling (the target of sunitinib) was not altered. The sunitinib resistant-RCC cell (786-O Res) when exposed to the sunitinib-TSA combination showed significant growth inhibition. Cells experiencing irreversible damage underwent apoptosis, causing an accumulation of cells in the sub-G1 population and the accumulation of cleaved PARP, introduced by caspase-3. In hepatoma cells (HepG2), Donia et al. [167] showed that TSA enhances responsiveness and induces apoptosis to Taxol. The sensitizing effect of acetylation modification on the responsiveness of hepatoma cells to anticancer therapy is ascribed to its modulatory role on epigenetics via the upregulation of HDAC1 and downregulation of Dnmt1 and 3α gene and drugs metabolizing genes.

Many authors reported that TSA potentiated radio and chemosensitivity in various cancer cells through different mechanisms. In this respect, Karagiannis et al. [37] treated human erythroleukemic K562 cells with TSA and then exposed them to anthracycline, doxorubicin, or gamma radiation. TSA pre-treatment increased the radio- and chemo-sensitization, inhibiting cellular proliferation, reducing clonogenic survival, and inducing apoptosis. The pretreatment of K562 cells with TSA augmented the cytotoxic effect of doxorubicin [38]. Similarly, findings [37] confirmed that TSA enhances the sensitivity of K562 cells to radiation which caused the accumulation of γH2A.X. Caspases-3 and 7 are involved in radiation-induced apoptosis. The evaluation of caspases-3 and 7 levels indicated that TSA, at concentrations higher than 0.3 µM, potentiated radiation-induced apoptosis in a concentration-dependent manner. The sensitization effect could involve histone hyperacetylation and changes in phosphorylated H2A.X formation on euchromatin at a lower dose of TSA, whereas, at the higher dose it could involve cytotoxicity and G1 (>0.3 µM TSA) and cell cycle arrest at the G2/M phase (1 µM TSA).

Moreover, Kim et al. [168] showed that TSA radio-sensitizes human head and neck cancer cell lines (HN-3 and HN-9 cells). TSA pretreatment (50 nM) significantly reduced the survival of HN-9 cells even at as low a radiation dose as 2 Gy (SF2) while in the HN-3 cell line, 200 nM TSA was necessary to show the effect [168]. In another study, Kim and coworkers showed that TSA enhances radiosensitivity by abrogating G_2_/M arrest in three cell lines (A549, HeLa, and Caski cells). The SF2 of TSA-treated cells was significantly lower than that of the mock-treated cells. The potent radiosensitivity observed is ascribed, at least in part, to the abrogation of radiation-induced G2/M arrest. Apoptosis was promoted when the cells were exposed to concentrations higher than 600 nM [169]. TSA also enhanced the radio-sensitivity of non-small cell lung cancer (NSCLC) (A549 and H1650 cells) to γ-irradiation [78]. In A549 cells, TSA caused both G1 and G2/M arrest and enhanced IR-induced accumulation of cells in the G2/M phase, with the upregulation of the expression of p21waf1/cip1 leading to cell cycle arrest. TSA co-treatment caused pronounced apoptosis through multiple pathways accompanied by p21waf1/cip1 cleavage. The enhanced apoptotic effect was mediated by the mitochondrial pathway, as indicated by the increased dissipation of mitochondrial transmembrane potential (MMP) and the release of cytochrome *c* from the mitochondria to the cytoplasm. Caspase-3 activation was also significantly increased, with more cleavage of PARP, associated with the repression of X-linked inhibitor of apoptosis protein (XIAP). TSA co-treatment also interfered with DNA damage repair processes and impaired DNA repair capacity after IR by the downregulation of Ku70, Ku80, and DNA-PKcs, as reflected by the enhanced and prolonged expression of γH2AX.

Using cervical cancer HeLa cells, researchers showed that TSA synergistically enhances the DNA targeting capacity and apoptosis-inducing efficacy of silver nanoparticles (AgNPs) due to its effect on chromatin condensation and through the activation of the apoptosis effector caspase. Significant ROS generation was observed upon AgNP and TSA treatment corroborating that oxidative stress contributes to the cellular effects of both compounds. A high number of γH2AX foci was detected, suggesting the enhanced formation of double-strand DNA breaks with the combination treatment [170]. TSA sensitized the hepatocellular carcinoma cells (HCC) (HepG2 cells) to enhance NK cell-mediated killing by regulating immune-related genes. In this regard, Shin et al. [171] observed a significant alternation in the immune-associated genes in TSA-treated HepG2 cells, particularly concerning innate immunity-related genes and antigen recognition-related genes. These findings suggest that TSA induces NK cell-mediated anti-tumor effects in HCC. TSA indirectly increased the killing of HCC cells by increasing NK cell-directed killing and directly by increasing apoptosis. TSA regulated the transcription of numerous innate immunity and tumor antigen recognition-associated genes, such as ULBP1 and RAET1G, in HCC cells. In addition, TSA treatment of HepG2 cells rendered them more susceptible to NK cell-mediated killing while increasing the expression of NKGD2 ligands, including ULBP1/2/3 and MICA/B. TSA also induced the direct killing of HCC cells by stimulating apoptosis. Furthermore, TSA treatment increased nuclear fragmentation and apoptotic bodies in a dose-dependent manner and increased the cleaved (active) caspase-3 in HepG2 and Huh7 cells whereas, PARP, a critical DNA repair protein, was also cleaved by TSA treatment. *In vivo*, TSA also reduced tumor cell growth in an NK cell-dependent manner in an established HCC tumor xenograft model in BALB/c nude mice [171].

Roh et al. [172] showed that TSA treatment reduced cell viability of human osteosarcoma (HOS) cell lines by increasing apoptosis via altering the cell cycle progression. TSA treatment increased the percentage of G2/M-phase cells, while producing a concomitant fall in the rate of G0/G1 phase cells, causing an increase in apoptotic cell portions. The expression levels of Bcl-2 and XIAP have decreased in a time-dependent manner. As loss of MMP is known to be a common event in apoptosis induction, a quick reduction of MMP and the release of cytochrome *c* to cytosol were observed. Apoptosis involves the generation of DNA fragmentation, activation of procaspase-3, cleavage of PARP, and increased DNA hypoploidy. TSA induced apoptosis in HOS cells in histone acetylation- and mitochondria-dependent pathways. It synergistically sensitized HOS (CRL-1543 and MG-63) cells to the action of genistein [172]. Listed in Table 5 are the anticancer activity of TSA through sensitization.

The sensitive effects the sensitizing effect of TSA on cancer cells towards drugs used in chemotherapy can be mediated by suppressing the resistance characteristics of tumor cells. In addition, the determination of the sensitizing molecular action of TSA could make it possible to set up the mechanisms of resistance to anticancer drugs.

### 6.3. Effect of Other Molecules on Enhancing the Anticancer Activity of TSA

Numerous studies showed that some anticancer drugs enhanced the anticancer efficacy of TSA. Wu et al. [179] reported that the addition of genistein enhanced the inhibition of growth of A549 lung cancer cells and increased apoptosis induced by TSA via, at least in part, the up-regulation of the TNF receptor-1 (TNFR-1) death receptor signaling pathway. TSA, in combination with genistein increased TNFR-1 mRNA and protein expression, while TSA alone exhibited no changes. Moreover, the same combination increased the activation of caspases-3 and -10 and p53 protein expression. Genistein enhanced the effect of TSA by increasing the expression of TNFR-1, which activated the caspase cascade and resulted in apoptosis. The silencing of TNFR-1 expression negatively affected the genistein’s effect on TSA’s anticancer efficacy in human lung cancer A549 cells [179]. Similarly, TSA-induced apoptosis was synergistically enhanced by quercetin through the mitochondrial pathway in human lung cancer A549 cells [175]. The expression level of p53 was potentiated by the treatment with a combination of TSA and quercetin. In parallel, p53 silencing did not completely inhibit the augmenting effect of quercetin on TSA-induced apoptosis, suggesting the contribution of an additional p53-independent pathway. Quercetin synergistically enhanced the TSA-induced acetylation of histones H3 and H4 suggesting that quercetin enhances TSA-induced histone acetylation by p53-independent mechanisms; this may contribute to the enhancing effect of quercetin on apoptosis. The cotreatment with TSA-quercetin increased the expression of many mitochondria-associated pro-apoptosis genes, including Apaf-1, Bax, and caspase-9, and resulted in a marked release of cytochrome *c* into the cytosol, which demonstrated, at least in part, a mitochondrial pathway mechanism. Moreover, the cotreatment with TSA-quercetin was tested in a xenograft tumor model in nude mice leading to potent inhibition of tumor growth through the upregulation of p53 protein and a higher level of apoptosis [175]. In another study, Chan et al. [176] showed that quercetin dose-dependently enhanced the antitumor effect of TSA by upregulating the expression of p53. Quercetin prevented TSA-induced muscle wasting, at least in part, through the activation of Forkhead box O1 (FOXO1), the suppression of muscle wasting associated proteins atrophy gene-1 and muscle ring-finger protein-1 expression and increasing the myosin heavy chain level in the gastrocnemius muscles. Moreover, quercetin attenuated TSA-increased oxidative damage and the pro-inflammatory cytokines [176]. TSA-induced apoptosis was potentiated by 18β-glycyrrhetinic acid in human epithelial ovarian carcinoma cell lines (NIH-OVCAR-3 and SK-OV-3 cells) as reported by Lee et al. [177]. It was suggested that 18β-glycyrrhetinic acid might potentiate the apoptotic effect of TSA against ovarian carcinoma cell lines by increasing the activation of the caspase-8 dependent pathway and the activation of the mitochondria-mediated cell death pathway, leading to the activation of caspases. In fact, TSA induced nuclear damage, decreased Bid and Bcl-2 protein levels, increased Bax levels, caused cytochrome *c* release, activated caspases-3, -8, and -9, and increased tumor suppressor p53 levels.

### 6.4. Anticancer Effect of TSA in Combination with Chemotherapy

In addition to the promising anticancer activity of TSA confirmed by the multiple studies mentioned above, a synergistic effect of this molecule with other compounds was also proven. In 2002, Chen and collaborators were among the first researchers who sought the synergistic activity of TSA and other compounds to tackle colon cancer [180]. They conducted an *in vitro* study combining TSA with butyrate, a fatty acid produced by microbial fermentation of dietary fiber in the intestinal tract, to assess their anticancer effect against the SW620 human colon cancer cell line. This combination induced the expression of DNA damage-induced gene 45α (GADD45α) and GADD45β, belonging to a family of classical tumor suppressor genes [43]. These genes also promoted DNA repair and removed methylation markers [181]. The same results were obtained by combining TSA with cycloheximide, an antifungal that inhibits protein synthesis in eukaryotic cells. A year later, Rahman et al. [44] verified this synergistic effect of TSA with other compounds in mouse (ddY mice) and rats (male Sprague Dawley rats) bone marrow cultures and murine macrophage cell line RAW264 to elucidate their role in osteoclastogenesis [44]. TSA and sodium butyrate (NaB) showed several positive results including the inhibition of osteoclast formation, inhibition of osteoclast-specific mRNA expression in RAW264 cells, and reduction of trans-activation of NF-κB-dependent reporter genes. In another study, Min et al. (2004) evaluated the anti-proliferative activity of TSA with HC-toxin in two human breast cancer cell lines, MCF-7 and MDA-MB-468 [182]. These authors observed a strong activity against both cell lines and the induction of apoptosis and cell cycle arrest at the G_2_/M phase.

Kang and al. [183,184] were among the first scientists who combined TSA with antioxidants to enhance the cytotoxic effect of combination therapy [183,184]. ROS are involved in various carcinogenesis stages [185], which explains the prolonged use of antioxidants in association with other anticancer drugs in the treatment of cancer. In the first study, and in addition to the cytotoxic activity observed in human leukemia cells (HL-60), the combined treatment decreased the generation of ROS. In contrast, in the second study it offered a protection against the Ni^2+^ cytotoxicity in human hepatoma Hep3B cells. Scavenging ROS was the primary mechanism of action by which quercetin exerts its anticancer activity. This attracted the attention of two Chinese researchers to combine it with TSA against HL-60 cells [144]. This idea allowed the cytotoxic activity to increase in a dose- and time-dependent manner. This highlighted previous findings on the effect of combination on promoting histone acetylation and scavenging ROS. As mentioned in the previous sections, TSA alone inhibits the proliferation of cancer cells by inducing apoptosis and cell cycle arrest, as well as the transcriptional activation of NF-κB and p21 regulated by PKC. Interestingly, the addition of calphostin C, a PKC inhibitor, to TSA-based therapy against esophageal and lung cancer cells reduced TSA-mediated upregulation of NF-κB and p21. The combination of histone deacetylase inhibitor (trichostatin A) and protein kinase C inhibitor (calphostin C) induced apoptosis of lung and esophageal cancer cells [186].

Similarly, Jeon and his coworkers assessed the antitumor effect of TSA combined with gemcitabine, a chemotherapy drug against human bladder cancer cell lines (HTB5, HTB9, T24, J82 and UMUC14). These researchers showed that TSA synergistically potentiated the antitumor effect of gemcitabine, triggering cell cycle arrest and apoptosis and inducing repression of NF-κB signaling pathway activation [187]. In pancreatic cancer, the combination of TSA with gemcitabine suppressed the proliferation of human pancreatic adenocarcinoma cell lines *in vitro* and induced cell apoptosis by increasing the expression of the pro-apoptotic BIM gene accompanied by the downregulation of the 5’-nucleotidase *UMPH* type II gene [42]. Moreover, *in vivo* studies in xenografts of pancreatic adenocarcinoma cells in nude mice showed that this combination reduced tumor mass to 50% [42]. Furthermore, Hammer et al. [188]. investigated the *in vitro* and *in vivo* anticancer effect of the combinatory treatment of TSA with interferon β (IFN-β), a type of immunomodulating molecule known for its strong antitumor action, against human neuroblastoma cells (NB-1691 and NB-1643) and retroperitoneal human neuroblastoma xenografts. Results demonstrated that TSA acted synergistically with IFN-β, inducing a decrease in cell count compared to the controls in human neuroblastoma NB-1691 and NB-1643 cell lines. This effect was accompanied by the upregulation of p21Waf1 expression levels, especially in NB-1691 cells [188].

On the other hand, *in vivo* experiments showed that this combinatory based-therapy significantly restricted tumor growth in the murine model of neuroblastoma. In this respect, combining TSA with another HDAC inhibitor, valproic acid, inhibited the growth of neuroblastoma cells with IC_50_ values ranging from 69.8 to 129.4 nM [189]. This combination induced the expression of CYP1A1, one of the main cytochromes P450 enzymes involved in the metabolism of carcinogens, which consequently potentiated its anticancer effect against UKF-NB-3 and UKF-NB-4 neuroblastoma cell lines [189]. In another study, using renal cell carcinoma (RCC) cells (SK-RC-39 and SK-RC-45 lines) and tumor xenograft model, Touma et al. [45] demonstrated that TSA and all-*trans* retinoic acid (ATRA) combinatory therapy might represent an effective strategy for the treatment of advanced RCC. These authors showed that TSA with ATRA suppressed the proliferation of RCC cell lines and tumor growth in a xenograft model through the reactivation of tumor suppressor genes such as the retinoic acid receptor β2 gene (*RARβ2*) mRNA expression (8 h after treatment). They also observed that TSA and ATRA combination induced apoptosis and partial G0-G1 arrest in RCC SK-RC-39 cell lines [45]. Interestingly, focusing on developing a novel therapeutic strategy against ovarian cancer, particularly taxane-resistant ovarian cancer, Jin et al. [190] reported the possible mechanism of the synergistic anticancer effect of TSA with a proteasome inhibitor PS-341 in ovarian cancer A2780 cell line and its resistant variant, A2780T cells. The combination of TSA with PS-331 induced cell cycle arrest at the G2/M phase and apoptosis, and inhibited cell proliferation in A2780 and A2780T cells associated with the overexpression of cyclin B1.

The Raf/MEK/ERK pathway has been the subject of intense investigations in the field of chemotherapy due to its multiple effects on cell growth, proliferation, prevention of cell-cycle arrest and apoptosis and the induction of drug resistance in different cell lines [191]. Thus, the Raf/MEK/ERK pathway represents an attractive target-based approach for cancer treatment. Addition of TSA to chemotherapeutic agents such as cisplatin, gemcitabine, or doxorubicin-induced synergistic cytotoxicity and concomitantly inhibited chemotherapeutic drug-induced activation of Raf-MEK-ERK signaling pathway in human urothelial carcinoma (UC) cells [159]. Activated Raf/MEK/ERK pathway is involved in the chemoresistant mechanism of UC [159]. These findings indicate that combining chemotherapeutic agents with TSA is a promising avenue to overcome the chemotherapeutic resistance of urothelial carcinoma cells via the inactivation of the c-Raf/ERK pathway.

In addition, Yan et al. [192] investigated the role of the combinatory effect of TSA with curcumin, a polyphenol pigment obtained from *Curcuma longa* (turmeric), in the treatment of breast cancer. These authors showed that the combination of these two compounds inhibited cell growth and viability of MDA-MB435eB and SkBr3 cell lines. Moreover, TSA with curcumin induced cell apoptosis and G0/G1 cycle arrest in SkBr3 cells and G2M arrest in MDA-MB435eB cells. The molecular mechanisms underlying this synergistic effect were the activation of caspase-3 and poly(ADP-ribose) polymerase-1 cleavage, accompanied by a decrease of ERK and Akt phosphorylation and the up-regulation of p38 and JNK pathways [192]. Similarly, Piao et al. [193] studied the potential synergistic effect of TSA and BEZ235 (dactolisib), a dual pan-class PI3K and mTOR inhibitor, on the development of non-small-cell lung cancer (NSCLC). They reported that these two drugs cooperated to inhibit NSCLC proliferation, migration, and invasion, as well as the NSCLC epithelial-mesenchymal transition (EMT) *in vitro*, and to induce cell apoptosis. In addition, xenograft studies revealed that TSA combined with BEZ235 suppressed tumor growth and metastasis, and induced tumor necrosis *in vivo* [193]. In breast cancer, this combinatory therapy exerted significant synergistic growth inhibition of multiple cell lines by targeting caspase-dependent apoptosis and autophagic cell death pathway [194]. Shown in Table 6 are the synergistic anticancer activity of TSA.

As can be seen, TSA can exhibit its anticancer action via different combinatory effects. Indeed, it potential the effects of used drugs in chemotherapy via its direct and/or indirect effects on cancer cell lines. This property should be explored and further investigations testing the combinatory effects of TSA with anticancer drugs in clinical trials.

### 6.5. TSA Targets Epigenetic Modifications in Cancer

Recent investigations showed that cancer cells are characterized by epigenetic instability and memory disruption. During cell differentiation and development, memory cells are installed and maintained under epigenom programs. Epigenomic programs involve epigenetic modifications, which design changes in gene expression without any change in the physical structure of DNA. Several enzymes are involved in these epigenetic modifications including DNAT (DNA methyltransferase), which is responsible for DNA methylation, HDAC (histone deacetylase) and HAT (histone acetylase) which are responsible for histone modifications. Current molecular investigations indicated that the disruption of epigenetic marks can lead to cell transformation and tumorigenesis. Certain pharmacological investigations revealed the role of some molecules called epidrugs against cancer. These molecules target epigenetic perturbations and exhibit remarkable anticancer properties. The TSA direct effects on cancer cell lines, its chemosensitizing agent towards chemotherapy, and its synergistic effect with other chemotherapeutic drugs, suggest its potential as an important epidrug molecule against different human cancers.

Ou et al. [227] surveyed the effect of TSA epigenetic regulation on histone modifications of human urinary bladder cancer T24 and human breast adenocarcinoma (MDA-MB-231) cells. Based on their investigation, TSA exhibited an increase in histone acetylation associated with a significant decrease in global methylation, induction of histone acetylation, demethylation, and expression of the methylated *E-CADHERIN* and *RARβ2* genes, as well as some gene selectivity toward the studied cell lines [227]. In a similar fashion, scientists showed a high degree of histone deacetylation inhibition of TSA, associated with potent repression of *MUC4* in high-expressing cells. Results highlighted the potential effects in preventing breast cancer with the use of 100 ng/mL of TSA combined with epigenetic modulator, genistein (GE), at a concentration of 25 μM. In this case, TSA inhibited HDAC, enhanced the re-expression of ERα in MDA-MB-231 cells, induced re-sensitization and reactivation of ERα-negative breast cancer cells to E2 and tamoxifen (TAM) antagonist, and promoted histone-remodeling changes in the ERα promoter [49,228,229].

The analysis of DNA methylation for a putative sphingosine-1-phosphate (S1P_1_) promoter using TSA combined with 5-aza-2’-deoxycytidine (Aza-dC) in human melanoma cell line A2058 demonstrated the epigenetic regulation of S1P receptors in examined cells lines [230]. These findings indicated that TSA and Aza-dC mixture induces a switch of S1P from a motility inhibitor to a stimulator, enhancing the expression of S1P_1_ and S1P_3_, associated with S1P-induced chemotaxis, and reduces the expression of S1P_2_ related with motility suppression [230]. Similarly, Vincent et al. [49] established the restoration of MUC4 expression in a cell-specific manner after the treatment of pancreatic (PANC-1, CAPAN-1, and CAPAN-2) and gastric (KATO-III) epithelial cancer cell lines undergoing epigenetic regulation by the same combination of TSA and Aza-Dc [49]. According to these authors, the chromatin immunoprecipitation and RNA interference techniques confirmed that DNMT3A and DNMT3B were directly implicated in MUC4 silencing by binding to its 5′-UTR in a cell-specific manner [49].

Choi et al. [231] were interested in the TSA possible epigenetic modulation mechanism responsible for the inhibition of *hTERT* in the human colon cancer cell line (HCT116). For the first time in their survey, these scientists found that TSA exhibits a significant epigenetic role by inducing the demethylation of site-specific CpGs on the promoter of hTERT, which was due to DNMT1 downregulation [231]. TSA was also found to promote the CTCF binding on the hTERT promoter, resulting in the suppression of hTERT [231]. It was noted that the treatment with GE and TSA inhibited cell growth, downregulated the DNMT1 gene expression after 48 and 72 h of treatment, and DNMT3a gene expression only after 72 h, and promoted apoptosis in all tested groups of Human HCC HepG2 cells [47]. Importantly, TSA may play a role in preventing hepatocellular carcinoma by inhibiting apoptosis and reducing the expression of DNMT1. The relative expression of the DNMT1 gene ranged from 0.5 to 0.19 [232].

Recently, Sanaei et al. [106] showed dose and time-dependent antiproliferative effects (IC_50_~1 μM) of TSA on hepa-6 cells, with a significant apoptotic action and a remarkable increase in the quantity of *ERα* gene expression [106]. To discover the main epigenetic pathways to limit the malignancy of ovarian cancer, Meng et al. [229] worked on the anti-ovarian cancer activity of TSA with decitabine. The treatment of SKOV3 cell line by TSA and decitabine significantly limited the activity of DNMTs, in particular, the expression of DNMT3A/3B. The combination therapy inhibited the invasion and tumorigenicity of ovarian cancer cells and suppressed migration capacity by the induction of E-cadherin and suppressing N-cadherin. The progression of the ovarian tumor was also repressed partially by the inhibition of MMP-9 and MMP-2 with this drug combination [229].

The micro-RNA (mRNA) was reported as another key in cancer epigenetic modification. Januchowski et al. [233] elucidated the role of TSA in Jurkat T leukemia cells clone E6-1 genetics character expression. By employing Western blot and quantitative real-time PCR methods, these researchers found that TSA can suppress the DNMT1 mRNA stability and protein expression in Jurkat T cells [233]. TSA increased the mRNA expression of the *DKK1* gene in colon cancer cells [234]. Human malignant lymphoma CA46 cells were subjected to TSA alone or combined with epigallocatechin-3-gallate (EGCG) [179,235]. Results revealed that TSA alone inhibited CA46 cell proliferation, and when TSA (15 ng/mL) was combined with EGCG (6 μg/mL), the proliferation of CA46 cells from 24 to 96 h was decreased [235]. The co-treatment with TSA and EGCG downregulated *p16^INK4A^* gene methylation, correlated with a rise in p16INK4A mRNA and protein expressions. This combination also reactivated *p16^INK4A^* gene expression partially by lowering promoter methylation and reducing the CA46 cell overgrowth [235]. The above-mentioned studies proved the promising chemopreventive properties of TSA alone or in combination with other compounds and could be employed as a potential target in the treatment of hepatocellular carcinoma, breast, ovarian, and colon cancers.

## 7. Conclusions and Concluding Remarks

In conclusion, we summarized the biological and pharmacological properties of TSA, particularly anticancer properties. In addition, this review highlighted that TSA exhibits antioxidant, anti-inflammatory, antidiabetic, and anticancer effects with different mechanisms. It also exhibits remarkable *in vitro* and *in vivo* actions against different cancer cell lines such as breast, skin, and neuronal cancers. The anticancer properties of TSA involve pharmacodynamic actions including apoptosis, autophagy, anti-angiogenesis, and anti-telomerase. The exact molecular mechanisms with therapeutic correlations should be investigated to clarify the pharmacodynamic action. Among the remarkable anticancer mechanisms of TSA is its action on epigenetic pathways involved in cell memory. Therefore, further studies should be carried out to validate the use of this substance as an epidrug against some types of human cancers. TSA was used also in combination with other approved drugs, and it was shown that TSA improves the efficacy and therapeutic index of some used drugs. Moreover, TSA has also been shown to sensitize the treatment of other drugs used in chemotherapy. All these findings indicate that TSA is a very promising candidate as an anticancer drug; however, further pharmacokinetic investigations should be carried out to validate its absorption, bioavailability, metabolism, and elimination. Moreover, toxicological studies on animals and possibly in humans should also be tested to confirm its safety. This review could also give perspectives on the use of other natural molecules with chemical structure similarities. Furthermore, it has been demonstrated that there is a stochasticity of the biological effects linked to TSA, and this suggests that other natural molecules which may be stereochemically similar, or may have different functional groups, can also exhibit several pharmacological actions.

## Figures and Tables

**Table 1:** Antioxidant effects of TSA.

Experimental Approach	Key Results	Ref
TGF-β-induced myofibroblast differentiation of corneal fibroblasts Immunofluorescence staining Reverse transcription quantitative-polymerase chain reaction Western blot analysis	Elevated intracellular GSH level and cellular total antioxidant capacity Decreased cellular ROS and H_2_O_2_ accumulation. Induced Nrf2 nuclear translocation Upregulated the expression of Nrf2-ARE downstream antioxidant genes	[14]
Human bone marrow-mesenchymal stem cells MTT assay Immunoblot analysis	Increased SOD2 Decreased intracellular ROS Suppressed H_2_O_2_-induced ROS generation Modulates FOXO1	[12]
H9c2 rat myocardial cell line Western blot analysis Chromatin immunoprecipitation assay	Decreased the levels of MDA Decreased the H_2_O_2_-induced levels of ROS Increased the expression of FoxO3a, SOD2 and CAT, and increased H4 acetylation of the FoxO3a promoter region	[11]
Human lens epithelial cells (HLECs) after UVB exposure Cell viability Western blot assay Enzyme-linked immunosorbent assay Real-time PCR	Suppressed BAX and caspase-3 expression Suppressed the expression of FOXO3A and MT2 Increased SOD levels Decreased MDA levels Decreased ROS levels Increased total antioxidant capacity	[13]

**Table 2:** Antidiabetic effects of Trichostatin A.

Experimental Approach	Key Results	Ref.
Streptozotocin (STZ)-induced diabetic rats Normal rat kidney tubular epithelial cells (NRK52-E) stimulated with TGF-β1	No effect on blood glucose or kidney/body weight ratio. Significantly decreased urinary protein/creatinine excretion Significantly increased glomerular and tubular fibronectin and collagen I and tubular α-SMA expression. Significantly decreased tubular E-cadherin expression. Increased E-cadherin expression at both mRNA and protein levels. Prevented ECM upregulation and EMT in NRK52-E cells. Prevented TGF-β1-induced downregulation of E-cadherin and upregulation of collagen I.	[16]
β-cell line βTC-tet, L-cell line GLUTag, or recombinant insulin-secreting L-cell lines Real-time PCR, ELISA, and radioimmunoassay	Significantly promoted insulin mRNA secretion in TSA-treated βTC-tet cells. Significantly promoted GLP-1 mRNA secretion in TSA-treated GLUTag cells. Significantly promoted insulin mRNA secretion in TSA-treated GLUTag-INS and EINS cells. Decreased mRNA levels of insulin and GLP-1 in β- and L-cells Caused a 2.5-fold increase in stored insulin and a 2-fold increase in glucose-stimulated insulin secretion in βTC-tet cells. Increased stored and MH-stimulated GLP-1 in GLUTag cells. Significantly increased EINS proinsulin and insulin secretion	[17]
Streptozotocin (STZ)-induced diabetic rats Rat Schwann cells	Enhanced the action potential amplitude of sciatic nerves. Increased BDNF expression Increased GRP78 expression regulated BDNF protein level Improved XBP-1s/ ATF6/GRP78 axis. Improved the binding of GRP78 and BDNF Improved the differentiation of SH-SY5Y cells	[15]

**Table 3:** Anti-inflammatory effects of Trichostatin A.

Experimental Approach	Key Results	Ref
Allergen-induced airway inflammation in a mouse asthma mode	Decreased inflammatory cells Reduced IL-4, IL-5, and IgE levels Reduced Th2 cytokines expression Decreased infiltration of CD4+	[18]
Lipopolysaccharide-(LPS)-stimulated macrophages	Inhibited the production of nitric oxide (NO) Reduced the mRNA and protein levels of the proinflammatory cytokines (TNF-α, IL-6, and IL-1β) Increased the level of the immunosuppressive cytokine IL-10 Decreased the cell surface markers of the maturity of the macrophages	[19]
Lipopolysaccharide (LPS)-induced production of IL-6 in OP9 Preadipocytes	Enhanced palmitic acid-induced IL-6 production Enhanced the expression of inflammatory genes. Increased the level of NF-kB p65 acetylation	[21]
Phorbol myristate acetate-induced macrophages	Reduced TNF-*α*, IFN-*γ*, IL-10 and IL-18 levels Suppressed the expression of class I HDACs Inhibited apoptosis of macrophages Reduced the viability of PMA-induced macrophages Suppressed the expression of proinflammatory genes Enhanced the acetylation of NF-κB p65 Promoted histone acetylation	[22]
LPS-induced acute lung injury model *in vitro*	Enhanced LPS-stimulated NR8383 cells Decreased the levels of TNF-α Upregulated the micorRNA-146a expression	[20]

**Table 4:** Direct anticancer activity of trichostatin A.

Origin	Used Model	Experimental Approach	Key Results	References
Purchased	RPMI8226 and MM.1S cells	Immunofluorescence Immunoprecipitation Western blot analysis qPCR	Induced cytotoxic effect in multiple myeloma cell lines Induced cell apoptosis Inhibited hedgehog signaling pathway	[50]
Purchased	YD-10B oral squamous carcinoma cells	MTT assay Cell cycle analysis Western blot analysis DAPI staining	Inhibited cell proliferation Arrested cell cycle progression at the G_2_/M phase Induced mitochondrial membrane destruction Induced cyto-c release and proteolytic activation of caspases-3 and -7	[23]
Purchased	MDA-MB-231 and MCF-7 human breast carcinoma and SK-UT-1B uterine cancer cell lines	Flow cytometry analysis RT-PCP	Induced cyclin D1 downregulation through both ERα-dependent and ERα-independent mechanisms	[51]
Purchased	MCF-7 cells	Cell proliferation assay Immunoblotting Flow cytometry analysis	Induced Akt dephosphorylation in a PP1-dependent manner, resulting in the activation of GSK3β in MCF-7 cells TSA-induced cytotoxicity was attenuated by the selective inhibition of GSK3β resulting in increased proliferation	[24]
Not reported	U87 glioblastoma cells	RT-PCP	Reduced proliferation and colony sizes resulting in G_2_/M arrest Inhibited tumorsphere formation	[52]
Not reported	Gastric cancer cells (MKN-45 and SGC-7901 cells)	MTT and BrdU immunofluorescence assays Soft agar assay Flow cytometry analysis Western blot analysis	Suppressed cell proliferation Induced apoptosis by regulating the PI3K/AKT signaling pathway in gastric cancer cells Induced cell cycle arrest at the G_1_ phase and apoptosis	[15]
Not reported	Two leukemic cell lines (CCRF-CEM and HL-60)	Flow cytometry analysis	The IC_50_ value of CCRF-CEM was 2.65 ± 0.3 μM The IC_50_ value of HL-60 was 2.35 ± 0.2 μM CCRF-CEM cells were reduced to 56.5%, 45.3%, and 40.2% on the first, third, and sixth days HL-60 cells were reduced to 55.6%, 45.2%, and 36.3% on the first, second, and fourth days	[53]
Purchased	Human osteosarcoma cells	Confocal microscopy Western blot analysis Flow cytometry analysis	Promoted osteosarcoma cell death Induced autophagy in U2OS cells Inhibited mTOR signaling pathway and enhanced FOXO1 transcriptional activity	[34]
Not reported	Pancreatic and colon carcinoma cell lines	Western blot analysis Real-time RT- PCR	Increased MDR1 mRNA levels Downregulated the upstream promoter responsible for the active P-glycoprotein expression	[54]
Purchased	Human colon adenocarcinoma cell lines DLD-1 and SW480	Viability assays Western blot analysis Gene expression microarrays	Reduced cell viability Reversed the upregulation of gene expression levels induced by gain of chromosome 7	[55]
Purchased	Human pancreatic endocrine tumor cell lines (CM, BON, and QGP-1)	Cell proliferation assay Cell cycle analysis 2-D gel electrophoresis	Inhibited cell growth by arresting the cell cycle in the G_2_/M phase and inducing apoptosis	[56]
Purchased	Lung cancer cells	mRNA extraction and qRT-PCR Colony formation assay Flow cytometry analysis Cell cycle analysis Western blot analysis	Inhibited proliferation, reduced colony formation, and induced cell cycle arrest and apoptosis Reduced the expression of Bcl-2 through the upregulation of miR-15a/16-1	[57]
Not reported	Human pancreatic cancer cell lines (PANC-1, SW1990, and MIATACA-2 cells)	MTT assay Hoechst 33258 staining Flow cytometry analysis RT-PCR and western blot analyses	Decreased cell viability in a dose-dependent manner in PANC-1 cells Increased apoptosis of PANC-1 cells Increased the expression levels of Bax and caspase-3 Downregulated the expression level of Bcl-2	[58]
Purchased	Osteosarcoma MG-63 cells	MTT assay TUNEL assay Annexin V staining Flow cytometry analysis	Inhibited cell proliferation Induced apoptosis of MG-63 cells Arrested the cell cycle in G_1_/G_2_ phase Inhibited the invasiveness of MG-63 cells	[59]
Purchased	Five human hepatoma cell lines	MTT assay TUNEL assay Semi-Quantitative RT-PCR Chromatin Immunoprecipitation (ChIP) assay	Inhibited cell growth Induced apoptosis Inhibited the gene expression profile in hepatoma cell lines	[60]
Not reported	Mouse model with L1 neoplastic tumors	Measurement of tumor size and mice body weight Preparation of four formulations for the *in vivo* study	Reduced neoplastic tumor growth using the semi-solid formulation applied to the skin Impaired the skin barrier function of neoplastic tumors	[61]
Purchased	A549 cells	DNA fragmentation assay Flow cytometry analysis RNA extraction and RT-PCR Western blot analysis	Inhibited the cell viability Induced the apoptosis of A549 cells Induced the proteolytic activation of caspases-3 and -9 Induced a concomitant degradation of poly(ADP-ribose)-polymerase protein Decreased the levels of COX-2 mPvNA	
Purchased	HCT116 human colon cancer cell lines	MTT assay Reporter assay RNA extraction and RT-qPCR Western blot analysis ChIP assay	Induced the endoplasmic reticulum (ER) stress in wild-type (WT) HCT116 cells Induced apoptosis and cell viability depending on p53	
Purchased	*Trypanosoma cruzi*	Flow cytometry analysis Transmission electron microscopy LC-MS/MS	Reduced protozoa proliferation and viability Altered the dynamics of the microtubule cytoskeleton Altered the segregation of kDNA, generating polynuclear cells with atypical morphology	[62]
Purchased	Human osteosarcoma MG63 cell line Human osteoblastic cell line hFOB 1.19	MTT assay Flow cytometry analysis Western blot analysis	Inhibited the growth of MG63 cells Promoted apoptosis through activation of p53 signaling pathway	[26]
Not reported	Keloid fibroblasts	MTT viability assay Hoechst staining Flow cytometry analysis RNA extraction and real time RT-PCR Western blot analysis	Inhibited the collagen synthesis and induced apoptosis in keloid fibroblasts	[63]
Purchased	MCF-7 cells	RQ-PCR analysis Western blot analysis	Reduced the phospholipase C gamma-1 (PLCγ1) transcript and protein levels in MCF-7 cells	[64]
Purchased	Human pancreatic carcinoma cell lines (BxPC-3, AsPC-1, and CAPAN-1)	Real-time PCR Immunoblotting	Inhibited the incorporation of BrdU into BxPC-3 cells. Inhibited the phosphorylation of ERK 1/2 and AKT in BxPC-3 cells. Induced an activation of the MAP kinase p38 in all three cell lines especially in BxPC-3 cells Increased the mRNA levels of bax in BxPC-3 cells only Increased cell cycle inhibitor protein p21*^Waf1^* levels in BxPC-3 and AsPC-1 cells	[30]
Purchased	MCF10A and MCF10A-ras cells	RT-PCR Western blot analysis	Activated apoptosis in MCF10A-ras cells only Activated the FOXO1 via P21 upregulation Induced autophagy in MCF10A and MCF10A-ras cells by blocking the mammalian target of rapamycin signaling pathway	[35]
Purchased	BGC-823 human gastric cancer cell line, MCF-7 cells, and KYSE-510 human esophageal squamous cell carcinoma (ESCC)	Immunocytochemistry assay RNA isolation and qPCR Western blot analysis Colony forming assay	Induced mesenchymal-like morphological changes in human cancer cells Increased the expression levels of mesenchymal markers and E-cadherin Reduced cancer cell mobility Reduced cancer cell colony formation	
Purchased	Human renal cell carcinoma (RCC) caki cell line	Flow cytometry analysis Western blot analysis Measurement of mitochondrial membrane potential Determination of caspase activity	Increased TRAIL-induced apoptotic cell death in Caki cells Elevated TRAIL-induced activation of caspases in Caki cells Enhanced the downregulation of Bcl-2 and truncation of Bid in TRAIL-treated Caki cells	[65]
Purchased	Molt-4 cell line	MTT assay Flow cytometry analysis Immunocytochemistry Western blot analysis	Inhibited the proliferation of Molt-4 cells (IC_50_ = 254.32 μg/L after 24 h of exposure) Decreased the percentage of G_0_/G_1_ cells and arrested cells in G_2_/M phase	[66]
Purchased	Human endothelial cell line (ECV304 cells)	MTT assay Northern blot analysis Western blot analysis Wounded cell migration assay	Increased thrombospondin-1 expression, which reduced ECV 304 cell migration Inhibited tube formation regardless of the presence of exogenous vascular endothelial growth factor	[67]
Purchased	Human leukemia cell line Molt-4	MTT assay Annexin-V-FITC staining RT-PCR Western blot analysis	Induced Molt-4 apoptosis Upregulated 310 genes and downregulated 313 genes	[31]
Purchased	Human malignant glioma LNT-229 and LN-308 cell lines NMRI nude mice	Viability and cell growth assays PCR analysis Caspase activity assay Athymic CD1-deficient NMRI nude mice	Induced the upregulation of natural killer group-2 member-D (NKG2D) ligands and immunogenicity in glioblastoma (GBM) cells Suppressed tumor growth of GBM xenografts (*in vivo*)	[27]
Purchased	Human dermal lymphatic endothelial cells	BrdU assay Flow cytometry analysis Western blot analysis Semi-quantitative RT-PCR	Decreased lymphangiogenesis by inducing apoptosis and cell cycle arrest via p21-dependent pathways	[68]
Not reported	C6 glioma cell line	Immunoblot analysis MTT assay Flow cytometry analysis ChIP assay	Decreased cell viability Induced C6 cell apoptosis Induced the p38MAPK and AMPK activation in C6 cells	[69]
Not reported	Human cervical carcinoma cell (Hela cells)	MTT assay RT-PCR	Inhibited cell viability Induced cell apoptosis Promoted the expression of apoptosis-related genes	[70]
Not reported	Two human ESCC cell lines, KYSE-150 and KYSE-450	Western blot analysis Transwell migration assay	Promoted cell migration by RelA K310ac-slug-EMT pathway	[71]
Not reported	Hepatocellular carcinoma (HCC) cell line Huh7	qRT-PCR Western blot and immunoprecipitation	Alleviated the specific subset of HCC, the hepatitis B virus X protein (HBx)-induced HCC in metabolic stress, through promoting sirtuin 3 (SIRT3) transcription	[72]
Not reported	A549 cells	Flow cytometry analysis	Induced the growth inhibition and morphological changes Inhibited cyclins and cyclin-dependent kinases (CDKs) expression Induced tumor suppressor p53 and Cdk inhibitors such as p21 and p27	[28]
Purchased	Oral squamous cell carcinoma (OSCC) lines HSC-3 and Ca9.22	Trypan blue staining MTT assay Western blot analysis	Decreased OSCC cell viability and proliferation Enhanced the expression levels of Bim protein Damaged mitochondrial membrane potential and increased cytosolic apoptosis-inducing factor (AIF) in Ca9.22 cells	
Purchased	Jurkat leukemia T cell clone E6-1 cells	RQ-PCR Western blot analysis	Induced ZAP-70, LAT, and SLP-76 transcript and protein downregulation in Jurkat leukemia T cells Reduced the half-life of ZAP-70, LAT, and SLP-76 mRNAs	[46]
Purchased	Keloid fibroblasts	MTT assay RNA extraction and RT-qPCR Flow cytometry analysis Western blot analysis	Inhibited cell proliferation in a time- and dose-dependent manner Induced alterations in the expression of numerous miRNA sequences Downregulated the expression of miR-30a-5p	
Not reported	HeLa and bovine aortic endothelial (BAE) cells	Western blot analysis Northern blot analysis MTT assay	Increased thrombospondin-1 (TSP-1) expression at both the mRNA and protein levels through transcriptional activation	[73]
Purchased	Four retinoblastoma cell lines	RT-PCR Western blot analysis ChIP assay Luciferase activity assay	Induced the expression of TβR-II mRNA Activated the TβR-II promoter Inhibited cell growth	[74]
Purchased	Human oral SCC cell line SAS, Ca9-22, and HSC	MTT assay Flow cytometry analysis Western blot analysis RT-PCR Confocal laser microscopic analysis	Enhanced the replication of the HSV-1 mutant through the activation of NF-κB Inhibited cell growth by inducing cell cycle arrest at G_1_	[75]
Not reported	HeLa cells	RT-PCR Western blot analysis	Upregulated the expression of p21*^WAF1^* and p16*^INK4A^* in various cell lines Downregulated the expression of cyclin A Upregulated the expression of gelsolin and fibronectin	[76]
Not reported	MDA-MB-231 human breast cancer cell	MTT assay	Decreased cell viability (IC_50_ = 100 ng/mL) Induced apoptosis Induced poly (ADP-ribose) polymerase-1 (PARP-1) cleavage and caspase-3 activation Upregulated the expression of CDK inhibitor p21^(WAF1/CIP1)^ protein Downregulated the expression of Bcl-2	[77]
Purchased	Bone marrow cells and calvarial osteoblasts collected from the tibias and femurs of ICR mice	TRAP staining RT-PCR Western blot analysis *In vivo* experiment	Inhibited osteoclastogenesis and bone resorption by suppressing the induction of c-Fos by RANKL	[78]
Not reported	HeLa cells	RT-PCR Western blot analysis ChIP assay	Activated p21^WAF1/CIP1^ expression through the downregulation of c-myc and the release of the repression of c-myc from the promoter	[79]
Purchased	Human bladder cancer cell line, BIU-87	MTT assay Flow cytometry analysis RT-PCR DNA fragmentation analysis	Inhibited bladder cancer cell proliferation Induced cell cycle arrest at the G_1_ phase Increased apoptotic cell death Increased p21*^WAF1^* mRNA expression	[32]
Purchased	Murine pro-B lymphoma FL5.12 cells	MTT assay DNA fragmentation assay Flow cytometry analysis Western blot analysis RT-PCR	Inhibited cellular proliferation Induced apoptosis Induced DNA fragmentation Increased the protein levels of cleaved caspase-3 and PARP Induced apoptotic protein Bim Inhibited PU.1	[80]
Not reported	RAW264.7 cells	RT-PCR Western blot analysis ChIP assay	Inhibited LPS-induced C/EBPδ, resulting in a positive effect on LPS-induced cox-2 expression in RAW264.7 cells	[81]
Not reported	Human colon cancer cell lines HCT116, HT29, SW480	Annexin-V staining qRT-PCR Western blot analysis	Altered the expression of cell cycle-associated genes in HCT116 cells Downregulated the gene expression of minichromosome maintenance protein-2 (MCM-2) Increased phosphorylated JNK, which was involved in the downregulation of MCM-2	[82]
Not reported	ZAP-Grg1 transgenic mouse line (*in vivo*) A549 cells Human umbilical vein endothelial cells (HUVECs)	Western blot analysis qRT-PCR MTT assay Electric cell-substrate impedance sensing (ECIS) analysis	Inhibited lung tumorigenesis in Grg1 transgenic mice Reduced the expression of ErbB1 and ErbB2 Reduced the expression of VEGF and VEGFR2	[83]
Purchased	Human ESCC cell lines KYSE-150 and EC9706	Transwell migration assay qRT-PCR Western blot analysis	Promoted esophageal squamous cell carcinoma cell migration and EMT through BRD4/ERK1/2-dependent pathway	
Purchased	HeLa and Caski cervical cancer cell lines	MTT assay Flow cytometry analysis qRT-PCR Western blot analysis	Suppressed cervical cancer cell proliferation and induced apoptosis and autophagy through the regulation of the PRMT5/STC1/TRPV6/JNK axis	[84]
Purchased	MCF-7 cells	Trypan blue staining qRT-PCR Western blot analysis	Reduced CYP19 transcript and protein contents in MCF-7 cells Lowered CYP19 transcript stability and significantly decreased the transcript’s half-life	[85]
Purchased	EC9706 cells	Annexin V-FITC/PI staining Western blot analysis MTT assay Flow cytometry analysis	Suppressed ESCC cell growth by inhibiting the activation of the PI3K/Akt and ERK1/2 pathways	[86]
Purchased	SK-MEL-3 melanoma cells	Fluorescence microscopy Flow cytometry analysis	Downregulated critical components of the MAPK/MEK/BRAF oncogenic pathway, initiating a mitotic arrest	[87]
Purchased	Human ovarian cancer cell lines, COC1 and its DDP-resistant subline, COC1/DDP	RT-PCR Western blot analysis MSP assay ChIP assay	No effect on the reactivation of hMLH1 expression in COC1/DDP cells	[88]
Purchased	HCT116 and HT29 cells	Annexin V-FITC PI staining Flow cytometry analysis Bax siRNA transfection Western blot analysis	Induced cell cycle arrest and apoptosis in colorectal cancer cells via p53-dependent and -independent pathways	[89]
Not reported	16 NSCLC cell lines	MTT assay RNA extraction and RT-PCR	Displayed strong antitumor activities in 50% of NSCLC cell lines	[90]
Purchased	Human pancreatic cancer cell lines	Oligonucleotide array hybridization Western blot analysis qRT-PCR	Altered the expression of pro- and anti-apoptotic genes in pancreatic adenocarcinoma cells	[91]
Not reported	CD4^+^ T cells isolated from erythrocyte-depleted spleen cell preparations from C57BL/6 mice	RNA extraction and qRT-PCR Flow cytometry analysis Western blot analysis Determination of ROS generation Annexin V-FITC staining	Induced a rapid decline in cytokine expression and accumulation of cells in the G_1_ phase of the cell cycle Induced apoptotic cell death Altered the expression of a subset of genes involved in T cell responses	[92]
Purchased	Human NSCLC lines (Calu-1, NCI-H520, NCI-H23, and NCI-H441)	Flow cytometry analysis Annexin-V staining Immunoprecipitation Western blot analysis	Inhibited cellular growth Induced apoptosis Reduced the percentage of cells in the S phase (10% to 23%) and increased G_1_ populations (10% to 40%) Increased the expression of p21 without significant effect on p16, p27, CDK2, and cyclin D1	[93]
Purchased	Canine mast cell tumor (MCT)	Trypan blue staining Acridine orange/ethidium bromide staining MTT assay Cell cycle analysis	Reduced the viable cell numbers Increased cell death by apoptosis Increased hypodiploid cells Reduced the G_0_/G_1_ and G_2_/M–phases	[94]
Purchased	A549 cells	MTT assay Cell morphology analysis Wound healing assay Western blot analysis RNA extraction and RT-q-PCR assay Docking methodology	Effectively inhibited radiation-induced EMT by: Altered epithelial and mesenchymal markers Modulated signaling molecules of TGFb1 pathway Inhibited cancer cell migratory potential in A549 cells Effectively bound to Snail, an enhancer of EMT	[95]
Purchased	HeLa cells	Flow cytometry analysis Immunofluorescence staining RT-PCR	Induced a delay at the G_2_/M transition, chromosome missegregation, and multi-nucleation Induced cell death Induced a transcriptional modulation of key regulator genes of the cell cycle (Cyclin B1, Plk1, Survivin, and p21*^WAF1/Cip1^*)	[96]
Purchased	MCF-7 cells	Western blot analysis qRT-PCR Transfection and luciferase reporter assays	Augmented ESR1 gene repression at the transcriptional level Downregulated ERα protein expression under hypoxic conditions through a proteasome-mediated pathway Inhibited cell proliferation under both normoxia and hypoxia conditions Enhanced hypoxia-induced repression of ESR1 and degradation of ERα	[97]
Purchased	Human TK6 lymphoblastoid cell line	Cell cycle analysis Annexin V staining Cytogenetic assays Immunoblot analysis	Induced apoptosis and G_1_ cell cycle arrest Induced chromosomal breakage Induced DNA breaks Induced aneuploidy	[98]
Purchased	Human ESCC cells, EC109 and KYSE150	qRT-PCR Immonochemistry Western blot analysis ChIP-qPCR Annexin-V/FITC staining	Significantly induced DNA damage in ESCC cells Induced Rad9 gene expression both at transcriptional and translational levels in EC109 cells alone Enhanced DNA damage and cell death	[99]
Not reported	Primary hepatocytes Hepatoma cells	Western blot analysis Northern blot analysis LDH release assay Caspase-3 activation assay	Inhibited hepatocyte proliferation No induction of apoptosis in primary hepatocytes Induced apoptosis in hepatoma cells Upregulated the expression of the anti-apoptotic protein Bcl(xL)	[100]
Not reported	267B1 human prostate epithelial cells	Fluorescence microscopy Agarose gel electrophoresis Flow cytometry analysis	Inhibited cell growth Induced apoptosis Inhibited the levels of IAP family members Activated caspases and NF-κB	[101]
Purchased	MCF10A and MCF10A-*ras* cell lines	Ras activation assay MTT assay DAPI staining of nuclei Flow cytometry analysis Western blot analysis	Induced morphological changes, apoptotic cell death and modulation of the cell cycle regulatory proteins in the MCF10A-*ras* cells Downregulated the expression of cyclin D1 and CDK4 Upregulated the expression of p21*^WAF1^* and p53 Induced cell cycle arrest at the G_1_ phase in MCF10A-*ras* cells Decreased hyperphosphorylation levels of the Rb protein	[101]
Not reported	Chronic lymphocytic leukemia (CLL) cells	Flow cytometry analysis ATP assay Immunoblotting qPCR	Acted via a dual anti-HDAC/Wnt mechanism with a high selectivity and efficacy in CLL	[102]
Purchased	Human SCLC DMS53 cells	Light microscopy Western blot analysis MTT assay	Induced morphological differentiation and inhibition of cell growth via cell cycle arrest and subsequent apoptosis	[103]
Not reported	Apoptotic-resistant MCF-7TN-R cells derived from MCF-7 cells	Clonogenicity assay microRNA microarray analysis	Altered the microRNA expression profiles in apoptosis-resistant breast cancer cells	[29]
Purchased	Human gastric epithelial cell line BGC-823	MTT assay Hoechst 33342 staining Western blot analysis RT-qPCR Immunohistochemistry	Inhibited cell proliferation Induced cell apoptosis Inhibited non-metastatic melanoma protein B (GPNMB) expression	[104]
Purchased	Plasmacytoid dendritic cells (PDC)	Cytokine ELISA RT-PCR Confocal microscopy	Inhibited the production of IFN-I, TRAIL and of the pro-inflammatory cytokines TNF-α and IL-6 by CpG-activated PDC Inhibited the production of IFNα by PDC cultured *in vitro* in the presence of serum obtained from systemic lupus erythematosus patients	[105]
Purchased	SW480 cells	AnnexinV-FITC PI staining qRT-PCR MTT assay Flow cytometry analysis	Inhibited cell growth Induced apoptosis IC_50_ = 1.5 μM Upregulated p21, p27, and p57 genes expression	
Not reported	Human hepatocellular carcinoma Hepa 1-6 cells	MTT assay qRT-PCR AnnexinV- FITC and PI staining	Inhibited cellular proliferation Induced apoptosis Increased ERα gene expression quantity	[106]
Not reported	Hepatocellular carcinoma HCCLM3, MHCC97H, and MHCC97L cell lines	MTT assay Cell apoptosis assay qRT-PCR	Induced apoptosis and inhibited cell growth through both mitochondrial/intrinsic and cytoplasmic/extrinsic apoptotic pathways	[47]
Purchased	U87 glioblastoma cells and tumorsphere-derived cells	Tumorsphere formation assay Colony formation assay RT-PCR Western blot analysis Cell migration assay Cell cycle analysis	Inhibited proliferation and altered cell cycle in U87 human GBM cells Induced senescence-like alterations in nuclear morphology in U87 cells Increased mRNA levels of C-Myc and reduced Oct4 mRNA in cells Reduced tumorsphere formation and sizes in U87 cell cultures	[52]
Purchased	B lymphoblastoid cell lines (LCLs), SNU-20 and SNU-1103 Epstein-Barr virus-negative Burkitt’s lymphoma cell line, BJAB	Flow cytometry analysis Trypan blue staining RNase protection assay RT-PCR Western blot analysis Immunofluorescence assay	Enhanced anti-tumor effect for EBV-associated tumors by inducing a cell cycle arrest, apoptosis, and by triggering an EBV lytic cycle	[107]
Purchased	HeLa and SiHa cells	Western blot analysis RNA extraction and RT-PCR ChIP assay Transfection and luciferase reporter assay Tumorigenicity in mice xenograft model	Suppressed the PMA-induced OPN gene expression Suppressed the PMA-induced c-Jun recruitment to the OPN promoter by inhibiting c-Jun expression Suppressed cervical tumor growth in response to PMA in NOD/SCID mice xenograft model	[108]
Purchased	SW480 and SW620 cells	Western blot analysis Immunofluorescence analysis Reporter assays ChIP assay	Modulated claudin-1 mRNA stability through the modulation of Hu antigen R and tristetraprolin in colon cancer cells	[109]
Purchased	Human nasopharyngeal carcinoma (NPC) cell line CNE2 and undifferentiated C666–1	CCK-8 assay RNA extraction and RT-PCR Western blot analysis Flow cytometric analysis Transwell migration assay Scratch wound healing assay	Inhibited cell proliferation and arrested the cell cycle at G_1_ phases Reduced PCNA, cyclin D1, cyclin E1, CDK2, p16, and p21 expressions and stimulated CDK6 levels Promoted Vimentin and Snail1 expression Induced the EMT in CNE2 and C666–1 cells	[110]
Purchased	Human lung adenocarcinoma A549 cells and normal lung epithelial cells	RNA extraction and RT-PCR Immunocytochemical staining Western blot analysis Migration assay Cell cycle assay Fluorescein isothiocyanate (FITC) permeability assay	Increased anguin-1/LSR, decreased CLDN-2, promoted G_1_ arrest, and prevented the migration of A549 cells Increased the expression of LSR and CLDN-2 and decreased that of CLDN-1 with or without TGF-β in normal human lung epithelial cells	[111]
Not reported	Male Kunming mice	Testis weighing and sperm collection Histological processing Immunofluorescence Fluorescence microscopy	Increased genetic recombination frequency of spermatocyte meiosis	[112]
Purchased	A2780 cells	Histopathology analysis Immunohistochemistry Flow cytometry analysis	Induced morphological cell transformation, with increased cytoplasm Inhibited cell proliferation Reduced mitotic activity Induced epithelial-like differentiation with increased cytokeratin expression	[113]
Not reported	Human neuroblastoma (NB) cell lines	MTT assay siRNA-mediated silencing Western blot analysis	Induced cell death in neuroblastic-type NB cells by increasing the acetylation of Ku70, a Bax-binding protein CBP, Bax, and Ku70 contribute to therapeutic response to TSA against NB	[114]
Not reported	Raji cells and normal peripheral blood mononuclear cells	Flow cytometry analysis TUNEL assay Annexin V/PI staining	Inhibited cell proliferation Induced apoptosis Induced accumulation of cells in G_0_/G_1_ or G_2_/M Decreased cell population in the S phase	[115]
Not reported	MCF-7, MDA-MB-231 and MCF-10A cell lines	MTT assay Colony-forming assay Western blot analysis Annexin V- FITC and PI staining Cytochrome C release assay	Inhibited cell viability and proliferation without affecting MCF-10A cell Induced cell apoptosis which was initiated by G_2_-M arrest and depending on mitochondrial ROS produced after reduced mitochondrial respiratory chain activity	[116]
Purchased	Human rhabdomyosarcoma cell lines RH30 and RD	Annexin V-FITC and PI staining Flow cytometry analysis Immunohistochemical staining RQ-PCR miRNA transfection	Inhibited rhabdomyosarcoma proliferation and induced differentiation through myomir reactivation	[117]
Purchased	MCF-7 and MB-MDA-231 cells	MTT assay Annexin V- FITC and PI staining Flow cytometry analysis	Induced cell growth inhibition via 15-Lox-1 associated with the elevation of 15-Lox-1 metabolite (13 (S)-HODE) Induced cell cycle arrest Induced apoptosis	[118]
Not reported	Female wild-type BALB/c mice	Flow cytometry analysis ELISA test Cell differential counting Histopathology analysis	Suppressed murine innate allergic inflammation by blocking group 2 innate lymphoid cell (ILC2) activation	[119]
Purchased	MCF-7, T47-D, SKBr-3, and MDA-MB-231 cell lines Tumor xenograft model	Flow cytometry analysis Immunoblotting RT-PCR *In vivo* liposome uptake Immunohistochemistry of tumor sections	Induced a long-term degradation of cyclin A and a proteasome-dependent loss of ERα and cyclin D1, allowed derepression of p21*^WAF1/CIP1^* and RhoB GTPase Induced G_2_/M cell cycle arrest Induced apoptosis Increased ERα mRNA and p21*^WAF1/CIP1^* protein expression Decreased cyclin A with a G_2_/M blockade and cleavage of PARP	[120]
Purchased	MCF-7, T-47D, ZR-75-1, BT-474, MDA-MB-231, MDA-MB-453, CAL 51, and SK-BR-3 cells	Cell proliferation assay Immunoprecipitation and western blot analysis Histopathology analysis	Inhibited cell proliferation Exerted antitumor activity *in vivo* when administered daily (500 μg/kg) by s.c. injection for 4 weeks	[121]
Purchased	Human tongue squamous cell carcinoma SCC-6 cell lines	MTT assay Cell cycle analysis Cell invasion assay Western blot analysis Annexin V-FITC PI staining	Inhibited cellular proliferation Induced apoptosis Blocked the cell cycle at S and G_2_/M phase Inhibited cellular invasion Inhibited hypoxia-induced accumulation of HIF-1α protein and VEGF expression under hypoxic conditions	[122]
Purchased	Fresh tissues of ESCC were obtained from six patients	Western blot analysis Immunohistochemistry Cell Invasion Assay	Inhibited ESCC cell invasion by approximately 75% Decreased MMP-2 and MMP-9 protein levels in ESCC cells	[123]
Purchased	AGS gastric cancer cells	CCK-8 experiment Flow cytometry analysis RT-PCR Western blot analysis	Inhibited cell proliferation and promoted cell apoptosis, leading to AGS cell cycle arrest in G_0_/G_1_ and G_2_/M phases, especially G_0_/ G_1_ phase Increased p21, p53, and Bax gene expression levels Decreased Bcl-2, CDK2, and CyclinD1 gene expression levels	[123]
Purchased	SW480 and PC3 cells	Transwell invasion and migration assay Western blotting analysis qRT-PCR ChIP assay	Induced the reversal process of EMT in SW480 and PC3 cells, resulting in attenuated cell invasion and migration abilities Decreased the expression of transcription factor Slug	[124]
Not reported	5,637 Urinary bladder cancer cells	MTT assay Cell cycle analysis Annexin V-FITC and PI staining Measurement of mitochondrial membrane potential Western blot analysis	Altered cell morphology and reduced cell viability Induced cell cycle arrest Induced cell death via apoptosis Induced apoptosis via the mitochondrial pathway by promoting MMP dissipation and caspase-9 Suppressed the PI3K-Akt signaling pathway Induced Sp1 downregulation and suppressed survivin expression	[125]
Purchased	MCF-7 cells	Transwell invasion and migration assay Wound healing assay RT-qPCR Western blot analysis Overexpression of SLUG	Reversed EMT and attenuated the invasive and migratory abilities of MCF-7 breast cancer cells	
Not reported	U937 human leukemic cells	Flow cytometry analysis Cell cycle analysis MTT assay	Induced the growth inhibition and morphological changes in a concentration-dependent manner Increased G_1_ cell population of the cell cycle of U937 cells Induced the population of apoptotic sub-G_1_ cells Inhibited cyclins, PCNA, and Cdks expression Induced Cdk inhibitors such as p16, p21, and p27	[126]
Purchased	Human endometrial stromal cell line	MTT assay Real-time RT-PCR Western blot analysis	Inhibited cell proliferation Increased PR-α, PR-β, AR, and FasL expression	[127]
Purchased	HL-60 cells	MTT assay Annexin V-FITC PI staining Flow cytometry analysis lmmunocytochemical assay	Inhibited cell proliferation IC_50_ = 100 ng/mL, at the 36th Induced apoptosis	[128]
Not reported	HeLa cells	RNA isolation and RT-qPCR	Negatively regulated the expression of ubiquitin-specific protease 22 (USP22) Interfered with the binding of RNA polymerase II to the USP22 promoter, directly suppressing its transcription TSA-induced apoptosis was attenuated by the overexpression of USP22 in HeLa cells	
Not reported	HeLa cells	MTT assay Hoechst 33258 staining Flow cytometry analysis qRT-PCR	Inhibited cell growth Induced apoptosis Decreased the proportion of cells in S phase and increased the proportion of cells in G_0_/G_1_ and/or G_2_/M phases Induced the overexpression of genes related to malignant phenotype, including an increase in p53, p21*^Waf1^* and p27^Kipl^	[129]
Purchased	MG-63, 786-0, HT1080 and HeLa cells	Western blot analysis Immunoprecipitation RNA isolation and qPCR Tumor xenograft (BALB/c nude mice)	Inhibited the HIF-2α protein expression Inhibited tumor growth and HIF-2α expression *in vivo* Destabilized HIF-2α in a proteasome dependent manner, which is unrelated to VHL	[14]
Purchased	HeLa cells	MTT Assay Flow Cytometric Analyses Measurement of the MMP Immunostaining Annexin V-FITC and PI staining	Reduced cell survival Induced an MMP collapse Apoptotic cell death and the MMP collapse induced by TSA were decreased by the co-treatment of cells with CytoD and LatB	[130]
Purchased	p815 murine mastocytoma cell line	Trypan blue staining Hoechst 33342 staining Western blot analysis Flow cytometry analysis Immunofluorescent staining	Induced apoptosis Reduced cell viability, and many apoptotic manifestations such as generation of DNA fragmentation, activation of caspase-3, cleavage of PARP, and increased of DNA hypoploidy Increased the expression level of Bad Decreased the level of Bcl-2, Bcl-xL, and X-linked inhibitor of apoptosis protein	
Purchased	Mature osteoclasts	Flow cytometry analysis RNA extraction and semi-quantitative RT-PCR Western blot analysis *In vivo* mouse calvarial resorption analyses	Induced osteoclast apoptosis Induced upregulation of p21*^WAF1^* in osteoclasts Inhibited RANKL-directed bone destruction *in vivo*	[131]
Purchased	HeLa cells	MTT assay Western blot analysis Annexin V staining Measurement of MMP Detection of intracellular O_2_^•−^ levels	Inhibited cell growth Induced apoptosis, caspase-3 activation, and the loss of mitochondrial membrane potential Increased O_2_^•−^ level and induced GSH depletion in HeLa cells The administration of Bcl-2 siRNA intensified TSA-induced HeLa cell death	[132]
Not reported	Prostate cancer cell line DU145	MTT assay Flow cytometry analysis Immunofluorescence staining Western blot analysis	Induced mitotic catastrophe of DU145 cells, including morphological changes, cell cycle arrest at G_0_/G_1_ phase, and abnormalities of mitosis Increased the multinuclear cells Inhibited survivin protein expression Increased the expression of P21 protein	[133]
Purchased	Human pancreatic cancer cell line BxPC-3	MTT assay Cell cycle analysis Annexin V staining miRNA microarray analysis Northern blot analysis	Inhibited pancreatic cancer cell viability Arrested cells in G_0_/G_1_ phase Induced apoptosis, accompanied by differential expression of microRNAs	[134]
Purchased	AML-12, 3T3-L1, MDCK, Hep-3B, A549, HeLa, and MCF-7 cells	Flow cytometry analysis Immunoblotting	Suppressed TGF-β1-induced apoptosis in normal hepatocytes but not in hepatoma cells Suppressed serum starvation-induced apoptosis in non-cancer cells but not in cancer cells Induced apoptosis in cancer cells but not in non-cancer cells Activated ERK1/2 in non-cancer cells but not cancer cells	[135]
Not reported	OVCAR-3 cells	MTT assay Western blot analysis Caspase assay kits	Inhibited cell viability Increased the expression of cytochrome c and P53 and the expression of caspases-3, -8, and -9 Enhanced the mitochondria-mediated apoptotic pathways	[136]
Purchased	HeLa cells	MTT assay Fourier transform infrared spectroscopy (FT-IR) Immunofluorescence Analysis FT-IR spectroscopic measurements and analysis	Inhibited cell proliferation Induced an elevated level of cellular acetylation and conformational/structural changes of proteins in the cells Induced a higher percent of α-helix structure accompanied by an increment of acetylation level in both histones and cytoskeleton proteins	[137]
Not reported	HeLa and HepG2 cells	Clonogenic assay	Improved radiation resistance by activating Akt/Nrf2-dependent antioxidation pathway in cancer cells	[138]
Not reported	MCF-7 cells	MTT assay Annexin-V/PI staining Cell cycle analysis RT-PCR	Inhibited cell proliferation Induced apoptosis Downregulated the expression of ERα, myc-c, cyclin-D, and Bcl-2	[139]
Purchased	SKOV-3 and A549 cells	MTT assay RNA extraction and qRT-PCR Vybrant apoptosis assay kit Flow cytometry analysis	Exerted dose and time dependent cytotoxicity effect on both cells Upregulated klf4 expression Induced apoptosis	[140]

**Table 5:** Anticancer activity of TSA through sensitization.

Origin	Used Model	Experimental Approach	Key Results	References
Purchased	Hepatoma cells (HepG2)	MTT assay Annexin V assay DNA extraction and qRT-PCR	Sensitized hepatoma cells to Taxol (an anticancer drug) more than 5-Aza-dC and dexamethasone	[167]
Not reported	Head and neck squamous cell carcinoma cell line UT-SCC-77	MTT assay Immunofluorescence Immunoblot analysis Measurement of lysosomal pH	Enhanced cisplatin-induced apoptosis by decreasing lysosomal pH	[36]
Purchased	MM1S and ARP-1 cells	XTT cell proliferation assay Propidium iodide staining Flow cytometry analysis RT-PCR analysis	Sensitized TNF-related apoptosis inducing ligand (TRAIL)-resistant myeloma cells by downregulating the expression of Bcl-2 and Bcl-XL Modulated the expression of Bcl-2 proapoptotic members	[162]
Not reported	HeLa cells	MTT assay Wound healing assay Immunocytochemistry Western blot analysis	Enhanced the DNA targeting capacity and apoptosis inducing efficacy of silver nanoparticles (AgNPs) most probably due to its effect on chromatin condensation	[170]
Purchased	Human breast cancer cell lines MDA-MB-231, Hs578T and ZR75-1	RNA isolation and RT-PCR Immunoblotting MTT assay ChIP assay	Sensitized estrogen receptor (ER) α-negative, antihormone-unresponsive breast cancer cells to tamoxifen treatment	[164]
Purchased	Human leukemia cell lines (HL60 and U937 cells)	AlamarBlue assay Hoechst 33342 staining Apo-one homogeneous caspase-3/7 assay RNA isolation and RT-PCR	Potentiated the etoposide-induced cytotoxicity and apoptosis Activated caspases and induced the loss of the mitochondrial membrane potential	[161]
Purchased	Human erythroleukemic K562 cells	MTT assay Clonogenic survival assay	Increased the radio- and chemo-sensitization of K562 cells Inhibited cell proliferation Reduced clonogenic survival Induced apoptosis	[38]
Purchased	K562 cells	Flow cytometry analysis Western blot assay Caspase-3 and caspase-7 activity assays Clonogenic survival assay	Enhanced radiation sensitivity and accumulation of γH2A.X Inhibited cell proliferation Reduced clonogenic survival Induced apoptosis	[37]
Not reported	HN-3 and HN-9, human head, and neck cancer cell lines	Clonogenic assay	Radiosensitized HN-3 and HN-9 cell lines	[168]
Purchased	A549, HeLa, and Caski cell lines	Clonogenic assay Western blot analysis Flow cytometry analysis	Enhanced radiosensitivity by abrogating G_2_/M arrest in all three cell lines	[169]
Purchased	MDA-MB-231, MCF7, and MCF10A	MTT assay Flow cytometry analysis Western blot analysis	Sensitized the human breast adenocarcinoma MDA-MB-231 cells towards TRAIL-induced apoptosis	[156]
Not reported	Wild-type (A2780WT) and cisplatin-resistant (A2780RES) human ovarian cancer cells	MTT assay Immunohistochemistry qRT-PCR	Instigated apoptosis, autophagy, inhibition of cell cycle progression, and consequently loss of cell viability in A2780 cells Improved cisplatin-induced apoptosis, cell cycle arrest, and autophagy in A2780 cells Boosted the cisplatin-induced, p53-dependent apoptosis	[39]
Purchased	Gastric cancer cell lines AGS, NCI-N87, SNU-1, and SNU-16 cells	Annexin V/PI staining Cell Counting Kit-8 qRT-PCR Western blot analysis	Potentiated TRAIL-induced antitumor effects via the inhibition of ERK/FOXM1 pathway	[80]
Purchased	Human urothelial carcinoma cell lines, NTUB1and T24	Western blot analysis MTT assay Annexin V staining *In vivo* xenograft mouse model	Reduced cell viability and enhanced cytotoxicity of three chemotherapeutic agents (cisplatin, gemcitabine, and doxorubicin) Potentiated the apoptotic effects of the three chemotherapeutic agents Suppressed the activation of Raf/MEK/ERK pathway associated with chemotherapeutic agent treatment TSA + chemotherapeutic agents synergistically inhibited cell viability Enhanced chemotherapy-induced antitumor effects (*in vivo*)	[173]
Purchased	Human ovarian cancer SKOV3 cells Hey8 cells	Methylene blue analysis Flow cytometric analysis Annexin V staining RNA extraction and qRT-PCR Western blot analysis	Sensitized ovarian cancer cells to TRAIL-induced apoptosis by the downregulation of c-FLIP_L_ via the inhibition of EGFR pathway TSA + TRAIL induced apoptosis and inhibited cell viability in SKOV3 and Hey8 cells	[101]
Purchased	Human pancreatic cancer cell lines	Crystal Violet method Determination of IC_50_ values	Enhanced the response of chemotherapeutic agents in inhibiting pancreatic cancer cell proliferation	[40]
Purchased	Osteosarcoma cell line HOS	Western blot analysis Immunoprecipitation Flow cytometry analysis MMP assay	Reduced HOS cell viability Induced HOS cell apoptosis Reduced MMP and cytochrome c release to the cytosol Sensitized HOS cells to the action of genistein (an antitumor agent)	[172]
Purchased	786-O, ACHN, and Caki-1 RCC cell lines	MTT assay under normoxic or hypoxic conditions Flow cytometry analysis Western blot analysis	Enhanced cytotoxic effects of sunitinib on RCC cells	[165]
Purchased	786-O, ACHN, and Caki-1 RCC cell lines	Western blot analysis Capillary electrophoresis time-of-flight mass spectrometry (CE-TOF-MS) MTT assay Flow cytometry analysis	Reduced sunitinib resistance by triggering intracellular metabolome shifts regarding energy metabolism	[166]
Purchased	Athymic BALB/c nude mice HepG2 cells	Microarray and data analysis NK cell infiltration RNA extraction and RT-PCR NK cell cytotoxic assay AnnexinV-FITC PI staining	Sensitized the hepatocellular carcinoma cells to enhanced NK cell-mediated killing by regulating immune-related genes	[171]
Purchased	Normal ovarian surface epithelium (OSE) cells	Flow cytometry analysis Immunoblotting *In vitro* caspases-3 and -9 assay	Restored Apaf-1 function independent of alterations in Apaf-1 expression Restored Apaf-1 function and sensitized cells to cisplatin-induced apoptosis	[157]
Not reported	Lung cancer cell line A549 and the CDDP- resistant derivative A549/CDDP	Hoechst 33258 staining Flow cytometry analysis Western blot analysis	Induced apoptosis in both A549 cells and A549/CDDP cells Enhanced the sensitivity of A549/CDDP cells to cisplatin, along with concomitant DAPK upregulation	[158]
Purchased	Bladder cancer cells (HTB9, J82, SW1710, T24, HTB5, UMUC14, and 253J)	Clonogenic assay Flow cytometry analysis Hoechst 33342 staining Colorimetric caspase activity assay	Synergistically enhanced the antitumor effect of cisplatin and resensitized cisplatin resistant bladder cancer cells	[41]
Purchased	Human gastric cancer cell lines OCUM-8 and MKN-74	MTT assay RT-PCR	Increased the efficiency of anticancer drugs Increased the expression of p21, p53, DAPK-1, and the DAPK-2 gene in both cancer cells Increased the expression of caspase-3 in OCUM-8, but not in MKN-74	[163]
Purchased	NSCLC cell lines A549 and H1650	Cell cycle analysis Clonogenic assay Annexin V-FITC and PI staining TUNEL assay Flow cytometry analysis Western blot analysis	Induced cell cycle arrest and apoptosis in A549 cells Enhanced radio-sensitivity of NSCLC cells Enhanced IR-induced G_2_/M arrest and apoptosis Increased IR-induced apoptosis via mitochondrial pathway Promoted IR-induced caspase-3 activation in association with repression of XIAP expression Radio-sensitized A549 cells through the downregulation of DNA repair proteins	[174]
Purchased	HCC cells	MTT assay Immunoblotting TUNEL assay Hoechst 33342 staining	Sensitized HBx-expressing liver cancer cells to etoposide treatment	[135]
Purchased	Human lung cancer cells (A549 and H1299)	Western blot assay Caspases-3 and -9 activities Tumor cell xenograft mouse model	TSA-induced apoptosis was enhanced by quercetin through the mitochondrial pathway in A549 cells The anticancer effect of TSA was enhanced by quercetin in the xenograft tumor model	[175]
Purchased	Male nude mice injected with A549 cells	TNF-α and IL-1β determination Immunoblot analysis Immunohistochemical staining	The anticancer effect of TSA was enhanced by quercetin	[176]
Not reported	Human epithelial carcinoma cell lines OVCAR-3 and SK-OV-3	MTT assay Quantitative analysis of DNA fragmentation Western blot analysis Flow cytometry analysis ELISA test	TSA-induced apoptosis was reduced by inhibition of casein kinase 2	[177]
Not reported	NIH-OVCAR-3 and SK-OV-3 cell lines	MTT assay Western blot analysis Flow cytometry analysis	TSA-induced apoptosis was potentiated by 18β-glycyrrhetinic acid	[178]
Purchased	A549 cells	Flow cytometry analysis RT-PCR Western blot analysis Caspase activity	The effect of TSA on inhibiting A549 cell growth was enhanced using genistein by increasing the expression of TNF receptor-1	[179]

**Table 6:** Synergistic anticancer activity of TSA.

Origin	Used Model	Experimental Approach	Key Results	References
Purchased	SW620 human colon cancer cell line	RNA isolation and Northern analysis Apoptosis analysis Anti-ac-histone H4 Western blot	TSA + butyrate induced the growth arrest and DNA damage gene 45α (GADD45α) and GADD45β TSA + cycloheximide super-induced the expression of GADD45α and β	[180]
Purchased	Male Sprague Dawley rats and ddY mice RAW264 cells	Flow cytometry analysis RT-PCR Luciferase assays Western blot analysis	Inhibited osteoclast differentiation in bone marrow cultures TSA + sodium butyrate inhibited osteoclast formation and osteoclast-specific mRNA expression in RAW264 cells stimulated with receptor activator of NF-κB ligand (RANKL) TSA + sodium butyrate reduced the sRANKL-stimulated or TNF-α–stimulated trans-activation of NF-κB–dependent reporter genes	[44]
Not reported	MCF-7 and MDA-MB-468 cells	DAPI staining Flow cytometry analysis	TSA + HC-toxin induced antiproliferative activity in both cell lines Induced cell cycle arrest at G_2_/M phases Induced apoptosis	[182]
Purchased	Human leukemia cells (HL-60)	Trypan blue staining Measurement of intracellular ROS generation	Increased cytotoxic activity in a time- and dose-dependent manner TSA + antioxidants decreased ROS generation	[184]
Purchased	Human hepatoma cells (Hep3B)	Trypan blue staining Flow cytometry analysis Measurement of the intracellular ROS generation	TSA + antioxidants synergistically protected against *in vitro* cytotoxicity of Ni^2+^ in Hep3B cells	[183]
Purchased	Human leukemia cells (HL-60)	Cell proliferation and viability assays MDA assay	TSA + quercetin increased cytotoxicity in a time- and dose-dependent manner	[144]
Purchased	Lung and esophageal cancer cells	MTT assay Transfection and luciferase assay Western Blot analysis	Increased the transcriptional activity of NF-κB and p21 TSA + calphostin C decreased TSA-mediated upregulation of NF-κB and p21 activation	[186]
Purchased	SK-RC-39 and SK-RC-45 RCC cell lines Tumor xenograft model (forty Swiss *nu/nu* mice)	Western blot analysis RNA extraction and RT-PCR Annexin V- FITC and PI staining	TSA + all-*trans* retinoic acid (ATRA) inhibited the proliferation of RCC cell lines and the tumor growth in a xenograft model TSA alone or/+ ATRA reactivated RARh2 mRNA expression in RCC cells TSA + ATRA induced the apoptosis and partial G_0_-G_1_ arrest in SK-RC-39 cells	[45]
Purchased	Eight diverse human pancreatic cancer cell lines	Cell viability assay RT-PCR	TSA + proteasome inhibitor PS-341 synergistically induced apoptosis in pancreatic cancer cells	[195]
Not reported	Human astrocytoma A172 cells		TSA + hyperthermia (heat shock) effectively induced apoptotic cell death	[196]
Not reported	Human glioblastoma A172 cells	MTT assay Flow cytometry analysis Western blot analysis RT-PCR	TSA + hyperthermia increased the thermos-sensitivity of A172 cells, resulting in cellular apoptosis	[197]
Purchased	Human leukemia cells (HL-60)	Cell proliferation and viability assays	TSA + curcumin increased cytotoxicity in a time- and dose-dependent manner	[198]
Purchased	Ten human pancreatic cancer cell lines Female nude mice	Cell cycle analysis Cell proliferation assay Immunoblot analysis RNA extraction and RT-PCR *In vivo* study: T3M4 human pancreatic cancer cells were s.c. injected into animals	TSA + gemcitabine synergistically inhibited the proliferation of human pancreatic adenocarcinoma cell lines (*in vitro*) Enhanced the apoptosis TSA + gemcitabine synergistically inhibited growth of human pancreatic adenocarcinoma cells (*in vivo*)	[42]
Purchased	Hut-78 T- and Raji B-lymphoma cell lines	RQ-PCR Western blot analysis	TSA + sodium butyrate + 5-aza-2′-deoxycytidine altered the expression of glucocorticoid receptor α and *β* isoforms	[199]
Purchased	Non-small cell lung carcinomas (NSCLC)	Immunoblot analysis Caspase activity assay	TSA + etoposide-induced apoptotic cell death in drug-resistant NSCLC cells TSA + etoposide induced apoptosis in a caspase-dependent manner accompanied by a crucial decrease in Bcl-xL expression	[160]
Purchased	Human neuroblastoma cell lines NB-1691 and NB-1643 Retroperitoneal human neuroblastoma xenografts	Western blot analysis Tumor volume measurement (*in vivo*)	TSA + interferon β induced a reduction in cell count compared to controls in NB-1691 and NB-1643 cell lines Increased the expression of p21*^Waf1^* in NB-1691 cells TSA alone or/+ interferon β significantly restricted tumor growth	[188]
Not reported	Human endometrial carcinoma cells of the line Ark2, KLE, and AN3	Trypan blue staining Annexin V and Hoechst staining Flow cytometry analysis Western blot analysis	TSA alone or/+ paclitaxel inhibited cell growth TSA alone or/+ paclitaxel increased apoptotic rates	[200]
Purchased	Human cancer cell lines U87 and T98 (both glioblastoma), SW480, MCF-7, HeLa	MTT assay Western blot analysis ELISA test Tumor-bearing mice	TSA + G47Δ synergistically induced cell proliferation TSA + G47Δ enhanced cyclin D1 and VEGF inhibition TSA + G47Δ enhanced anti-angiogenesis and enhanced antitumoral efficacy in animal models	[201]
Purchased	LNCaP prostate cancer cell line	RNA extraction and RT-PCR	TSA + somatostatin + 5-aza decitabine upregulated the somatostatin receptor expression	[202]
Purchased	Human leukemia HL 60 cells	MTT assay Flow cytometry analysis Trypan blue staining Western blot analysis NF-κB transcription factor assay	TSA (100 nM) + EEAC (100 μg/mL) caused synergistic inhibition of cell growth and an increase of apoptotic induction EEAC could effectively increase the cytotoxic sensitivity of TSA through the upregulation of DR5 and NF-κB activation	[203]
Not reported	Human neuroblastoma lines	MTT assay 32P-postlabeling assay	TSA + valproic acid increased the cytotoxicity of ellipticine (an anticancer drug)	[204]
Purchased	UKF-NB-3 and UKF-NB-4 neuroblastoma cell lines	MTT assay Western blot analysis	TSA + valproic acid inhibited the growth of neuroblastoma cells (IC_50_ values ranging from 69.8 to 129.4 nM) TSA + valproic acid induced CYP1A1 expression and depressed CYP1B1 levels, in UKF-NB-4	[189]
Purchased	Human pancreatic endocrine tumor cell lines (CM, BON, and QGP-1)	Cell proliferation assay Flow cytometry analysis Annexin V- FITC and PI staining	Induced cell cycle arrest TSA + 5-aza-2′-deoxycytidine synergistically inhibited cell proliferation TSA + 5-aza-2′-deoxycytidine synergistically induced apoptotic cell death Regulated 19 proteins in both ductal and endocrine pancreatic cancer cells	[205]
Purchased	OVCAR-3 and SK-OV-3 cells	Colorimetric assay Clonogenic assays	TSA + Apicidin enhanced the radiosensitivity of ovarian carcinoma cells	[206]
Not reported	A549 cells	MTT assay Flow cytometry analysis Caspase-3 activity Comet assay	Genistein + β-carotene enhanced the cell- growth-arrest effect of TSA IN A549 cells	[207]
Purchased	UKF-NB-3 and UKF-NB-4 neuroblastoma cell lines	MTT assay Flow cytometry analysis Western blot analysis	TSA + valproic acid increased cytotoxicity of ellipticine and DNA adduct formation by ellipticine in human neuroblastoma cells	[208]
Not reported	Human bladder cancer cell lines HTB5, HTB9, T24, J82, UMUC14 and SW1710	Clonogenic assay Flow cytometry analysis Western blot analysis	Synergistically potentiated the antitumor effect of gemcitabine TSA + gemcitabine repressed NF-κB signaling pathway activation	[187]
Purchased	Human laryngeal carcinoma cell line Hep-2 Male BALB/c mice	Cell Counting Kit-8 (CCK-8) assay Hep-2 transplanted tumor growth in nude mice	TSA + 5-aza-2′-deoxycytidine suppressed cell proliferation on Hep-2 *in vivo* and *in vitro*	[209]
Not reported	MCF-7 cells	MTT Flow cytometry analysis	Inhibited E2-induced proliferation of MCF-7 cells TSA + raloxifene enhanced the antiproliferative activity of each other by promoting cell death via apoptosis and cell cycle arrest TSA alone or/+ raloxifene increased the expression level of estrogen receptor b (ER*β*)	[210]
Purchased	Human breast cancer cell lines, MDA- MB435eB, and SkBr3	MTT assay Annexin V-FITC Staining Cell cycle analysis Western blot analysis	TSA + curcumin decreased the viability of SkBr3 and 435eB cells TSA + curcumin enhanced the growth inhibitory effects of either compound alone TSA + curcumin decreased phosphorylation of ERK and Akt TSA + curcumin induces a G_0_/G_1_ arrest in SkBr3 cells and a G_2_M arrest in 435eB cells TSA + curcumin induced apoptosis TSA + curcumin induce phosphorylation of p38 and JNK in SkBr3 cells	[192]
Purchased	A549 cells	MTT assay Hoechst 33258 staining Flow cytometry analysis Immunofluorescence analysis Western blot analysis	TSA + docetaxel or erlotinib produced synergistic inhibition on A549 cells TSA + docetaxel or erlotinib induced apoptosis of A549 cells TSA + docetaxel or erlotinib induced a delay at G_2_/M transition TSA + docetaxel or erlotinib increased the expression of cleaved-caspase-3 TSA + docetaxel increased acetylation of α-tubulin	[136]
Purchased	A549 cells	Trypan blue staining Hoechst 33258 staining Flow cytometry analysis Western blot analysis	TSA alone or/+ paclitaxel reduced cell proliferation TSA + paclitaxel induced apoptosis and more cells arrested in G_2_/M phase TSA + paclitaxel synergistically increased acetylated tubulin, caspase-3, and PARP, TSA + paclitaxel reduced surviving expression	[211]
Purchased	Patients with AML Human leukemia HL60, KG1, Kasumi, K562, and THP1 cells	Western analysis Flow cytometry analysis ChIP assay	TSA + chaetocin dramatically induced apoptosis and enhanced tumor suppressor gene re-expression TSA + chaetocin enhanced antileukemic activity in leukemia cells derived from patients with AML	[212]
Purchased	Human osteosarcoma cell lines (MG-63, HOS, SaOS-2, and U20S) and murine osteosarcoma cell line LM8	MTT assay Cell cycle analysis Annexin V staining Quantitative PCR Western blot analysis *In vivo* xenograft study	TSA (0.3 μM) + metformin (10 mM) decreased the viability of osteosarcoma cell lines TSA + metformin arrested the cell cycle of MG-63 and LM8 in G_1_/G_2_ phase Suppressed *in vivo* tumor proliferation	[213]
Purchased	Human ovarian cancer cell lines HEY, SKOV3	MTS assay Cell migration assay Western blot analysis Mouse xenografts	TSA + 5-aza-20-deoxycytidine + cisplatin (low-dose) significantly suppressed cell viability, migration, and spheroid formation and growth TSA (0.3 mg/kg) significantly suppressed tumorigenicity of HEY xenografts through inhibition of EMT and decreased pluripotency of ovarian cancer cells	[89]
Purchased	LNCaP and PC3 cells	WST-1 assay Western blot analysis RT-PCR	TSA + bortezomib synergistically induced apoptosis in both cancer cells TSA + bortezomib effectively inactivated NF-κB signaling TSA + bortezomib upregulated the predominant endogenous apoptotic factor caspase-3, and disrupted the NF-κB pathway in the androgen-independent PC3 cell line	[214]
Purchased	Human lung adenocarcinoma A549 cells	MTT assay and Hochst33258 staining Western blot analysis	TSA + cisplatin induced synergistic anti-tumor effects (induced apoptosis, inhibited cell proliferation, increased the inhibition rate, decreased pro-caspase-8, and increased caspase-8)	[137]
Purchased	Two CCA cell lines (poorly differentiated KKU-100 and well-differentiated KKU-M214 adenocarcinoma cells)	MTT assay Flow cytometry analysis Western blot analysis	Induced G_0_/G_1_ phase arrest in KKU-100 cells Hydroxamic acid + TSA dose-dependently reduced the viability of both cells Hydroxamic acid + TSA exerted higher cytotoxicity than drugs alone Hydroxamic acid + TSA induced more apoptotic cell death of both cells than the single drug	[215]
Not reported	HEp2 human laryngeal cancer cell line	Annexin V/propidium iodide staining Western blot analysis TUNEL assay MTT assay	TSA + genistein inhibited cell growth and cell migration, and promoted apoptosis in the HEp-2 cells TSA + genistein reversed endothelial growth factor-induced epithelial-mesenchymal transition (EMT) in the HEp-2 cells	[216]
Purchased	MCF-7 and HeLa cells	WST-8 assay Measurement of oxidative stress markers Measurement of MMP TUNEL assay Caspase-3 assay	TSA alone or/+ palladium nanoparticles (PdNPs) inhibited cell viability TSA + PdNPs had a more pronounced effect on cytotoxicity, oxidative stress, MMP, caspases-3/9 activity, and expression of pro- and anti-apoptotic genes	[217]
Not reported	A549 and H460 human lung cancer cell lines	Wound healing assay Flow cytometry Hoechst 33342 staining Western blot analysis A549 xenografts (female BALB-C/nude mice) and metastases tissues collection	TSA + BEZ235 synergistically inhibited NSCLC cell proliferation and induced apoptosis Synergistically suppressed NSCLC migration and invasion Decreased xenograft growth and metastasis rates and ki-67 protein expression *in vivo*	[193]
Purchased	Six human breast cancer cells	MTT assay Flow cytometry analysis Colony formation assay Immunofluorescence staining Western blot analysis Female nude mice aged 4~6 week	TSA + BEZ235 induced significant synergistic growth inhibition of multiple breast cancer cell lines TSA + BEZ235 induced apoptosis in a caspase-dependent manner TSA + BEZ235 enhanced autophagic cell death TSA + BEZ235 blocked tumour growth without noticeable side effects	[194]
Purchased	HeLa cells	Fluorometric activity assay Enzymatic-linked immuno-captured ELISA Affymetrix miRNA 4.1-panel arrays	TSA + *Vitis vinifera* extract induced the overexpression of similar miRNAs predicted to destroy certain influential oncogenes	[218]
Purchased	Ovarian cancer A2780 cell line	Flow cytometry analysis Western blot analysis Immunofluorescence assay Annexin V assay	TSA + PS-341 increased apoptosis and G_2_/M arrest TSA + PS-341 enhanced the expression of cyclin B1, resulting in the proliferation inhibition and apoptosis in A2780 and A2780T cells	[190]
Not reported	Panc1 and PaCa44 pancreatic cancer-derived cells	Trypan blue staining MTT assay BrdU assay Western blot analysis	TSA + valproic acid induced apoptosis in both cancer cells Increased the pro-apoptotic Bim level, reduced the anti-apoptotic Mcl-1 level and increased ROS production and autophagy in PaCA44 cells	[219]
Purchased	Human Huh7 hepatocellular carcinoma cell line	MTT assay Western blot analysis	TSA + sorafenib inhibited cell viability TSA + sorafenib increased cytotoxicity of human hepatocellular carcinoma cells	[220]
Not reported	Human Burkitt’s lymphoma (BL) cell lines Ramos and Namalwa cells	MTT assay Trypan blue staining Cell cycle analysis Annexin V staining Western blot analysis BALB/c nude mice	Reduced cell viability, induced apoptosis, and cell arrest at G_0_/G_1_ Attenuated EPS8 and downstream Phospho-Erk1/2 pathway Knockdown of EPS8 + TSA had a synergistic suppression effect on BALB/c nude mice	[221]
Not reported	Human urothelial carcinoma (UC) cell lines (T24 and NTUB1) Xenograft nude mouse model	MTT assay Flow cytometry analysis Western blot analysis	TSA + one of the chemotherapeutic agents induced synergistic cytotoxicity and concomitantly suppressed chemotherapeutic drug-induced activation of Raf-MEK- ERK pathway TSA + chemotherapy elicited a synergistic cytotoxic response via targeting the Raf/MEK/ ERK pathway	[159]
Purchased	ESCC tissue from esophageal cancer patients Human ESCC cells Eca-109 and TE-1	Immunohistochemistry MTT assay Western blot analysis	Decreased the expression of both Beclin-1 and LC3 proteins in ESCCs TSA + BEZ235 inhibited synergistically ESCC cell viability and induced autophagy with increasing expressions of Beclin-1, LC3-II, and the ratio of LC3-II/LC3-I	[222]
Purchased	Human lung cancer H1299 cells	MTT assay Annexin V-FITC and PI assay Western blot analysis Quantitative RT-PCR	TSA-induced apoptosis was increased by 88% by quercetin in H1299 cells TSA-induced death receptor 5 (DR5) mRNA was increased by quercetin in H1299 cells TSA + quercetin significantly increased p300 expression	[223]
Purchased	Two human urothelial carcinoma (UC) cell lines (BFTC-905 and BFTC-909)	Western blot analysis MTT assay Western blot analysis *In vivo* xenograft Tumor size measurement	Enhanced the cytotoxicity of paclitaxel and reduced viability in human UC cells Potentiated the apoptotic effect of paclitaxel on UC cells TSA + paclitaxel synergistically inhibited viability in human UC cells	[224]
Not reported	SiHa and HeLa cells	Flow cytometry analysis MTT assay CCK-8 assays Colony formation assays Xenograft experiment Western blot analysis	TSA + cisplatin inhibited cell viability and colony formation ability TSA + cisplatin downregulated the protein expression of HPV16/18E7 and upregulated that of RB1	[225]
Not reported	MDA-MB- 231 and MCF-7 cells	RT-PCR Flow cytometry analysis MTT assay Cell cycle analysis Cell migration assay	TSA + Zebularine sensitized breast cancer towards TRAIL treatment in 231-EGFP cells, validating the potentiality of E-cadherin as a biomarker of TRAIL treatment efficacy in the invasive breast cancer	[226]

## Data Availability

Data sharing not applicable.

## Abbreviations

BDNF	Brain Derived Neurotrophic Factor
CDK	Cyclin Dependent Kinase
CLL	Lymphocytic Leukemia
CytoD	Cytochalasin D
EBV	Epstein-Barr Virus
GPNMB	Glycoprotein non-metastatic melanoma protein B
hBM-MSCs	Human bone marrow-mesenchymal stem cells
HIF	Hypoxia-Inducible Factor
LatB	Latrunculin B
LPS	Lipopolysaccharide
MCT	Mast Cell Tumor
MDA	Malondialdehyde
NPBMNC	Normal Peripheral Blood Mononuclear Cells
NPC	Nasopharyngeal Carcinoma
OPN	Osteopontin
PARP	PolyADP-Ribose Polymerase
PCNA	Proliferating Cell Nuclear Antigen
RMS	Rhabdomyosarcoma
ROS	Reactive Oxygen Species
RSC96	Cultured Rat Schwann Cells
SOD	Superoxide Dismutase
STZ	Streptozotocin
T-AOC	Total Antioxidant Capacity
TRAIL	TNF-Related Apoptosis-inducing Ligand
TSA	Trichostatin A
UPR	Unfolded Protein response
UVB	Following Ultraviolet-B
VEGF	Vascular endothelial growth factor
WAF1	Wild-type P53-activated Fragment 1

## References

[1] Sharifi-Rad J., Dey A., Koirala N., Shaheen S., El Omari N., Salehi B., Goloshvili T., Cirone Silva N.C., Bouyahya A., Vitalini S. (2021). Cinnamomum Species: Bridging Phytochemistry Knowledge, Pharmacological Properties and Toxicological Safety for Health Benefits. Front. Pharmacol..

[2] Bouyahya A., Chamkhi I., Benali T., Guaouguaou F.-E., Balahbib A., El Omari N., Taha D., Belmehdi O., Ghokhan Z., El Menyiy N. (2021). Traditional Use, Phytochemistry, Toxicology, and Pharmacology of *Origanum Majorana* L.. J. Ethnopharmacol..

[3] El Omari N., Bakrim S., Bakha M., Lorenzo J.M., Rebezov M., Shariati M.A., Aboulaghras S., Balahbib A., Khayrullin M., Bouyahya A. (2021). Natural Bioactive Compounds Targeting Epigenetic Pathways in Cancer: A Review on Alkaloids, Terpenoids, Quinones, and Isothiocyanates. Nutrients.

[4] Bouyahya A., Guaouguaou F.-E., El Omari N., El Menyiy N., Balahbib A., El-Shazly M., Bakri Y. (2021). Anti-Inflammatory and Analgesic Properties of Moroccan Medicinal Plants: Phytochemistry, *in Vitro* and *in Vivo* Investigations, Mechanism Insights, Clinical Evidences and Perspectives. J. Pharm. Anal..

[5] El Omari N., Bakha M., Imtara H., Guaouguaoua F.E., Balahbib A., Zengin G., Bouyahya A. (2021). Anticancer mechanisms of phytochemical compounds: Focusing on epigenetic targets. Environ. Sci. Pollut. Res..

[6] Alqahtani A.S., Ullah R., Shahat A.A. (2022). Bioactive Constituents and Toxicological Evaluation of Selected Antidiabetic Medicinal Plants of Saudi Arabia. Evid. Based Complement. Altern. Med..

[7] Morgan E.W., Perdew G.H., Patterson A.D. (2022). Multi-Omics Strategies for Investigating the Microbiome in Toxicology Research. Toxicol. Sci..

[8] El Menyiy N., Mrabti H.N., El Omari N., Bakili A.E., Bakrim S., Mekkaoui M., Balahbib A., Amiri-Ardekani E., Ullah R., Alqahtani A.S. (2022). Medicinal Uses, Phytochemistry, Pharmacology, and Toxicology of Mentha Spicata. Evid. Based Complement. Altern. Med..

[9] Tsuji N., Kobayashi M., Nagashima K., Wakisaka Y., Koizumi K. (1976). A New Antifungal Antibiotic, Trichostatin. J. Antibiot..

[10] Singh S.B., Genilloud O., Peláez F., Liu H.-W., Mander L. (2010). 2.05-Terrestrial Microorganisms–Filamentous Bacteria. Comprehensive Natural Products II: Chemistry and Biology.

[11] Guo Y., Li Z., Shi C., Li J., Yao M., Chen X. (2017). Trichostatin A Attenuates Oxidative Stress-Mediated Myocardial Injury through the FoxO3a Signaling Pathway. Int. J. Mol. Med..

[12] Jeong S.-G., Cho G.-W. (2015). Trichostatin A Modulates Intracellular Reactive Oxygen Species through SOD2 and FOXO1 in Human Bone Marrow-Mesenchymal Stem Cells. Cell Biochem. Funct..

[13] Qiu X., Rong X., Yang J., Lu Y. (2019). Evaluation of the Antioxidant Effects of Different Histone Deacetylase Inhibitors (HDACis) on Human Lens Epithelial Cells (HLECs) after UVB Exposure. BMC Ophthalmol..

[14] Yang L., Qu M., Wang Y., Duan H., Chen P., Wang Y., Shi W., Danielson P., Zhou Q. (2013). Trichostatin A Inhibits Transforming Growth Factor-β-Induced Reactive Oxygen Species Accumulation and Myofibroblast Differentiation via Enhanced NF-E2-Related Factor 2-Antioxidant Response Element Signaling. Mol. Pharmacol..

[15] An J., Zhang X., Jia K., Zhang C., Zhu L., Cheng M., Li F., Zhao S., Hao J. (2021). Trichostatin A Increases BDNF Protein Expression by Improving XBP-1s/ATF6/GRP78 Axis in Schwann Cells of Diabetic Peripheral Neuropathy. Biomed. Pharm..

[16] Noh H., Oh E.Y., Seo J.Y., Yu M.R., Kim Y.O., Ha H., Lee H.B. (2009). Histone Deacetylase-2 Is a Key Regulator of Diabetes- and Transforming Growth Factor-Beta1-Induced Renal Injury. Am. J. Physiol. Renal Physiol..

[17] Tiernan A.R., Champion J.A., Sambanis A. (2015). Trichostatin A Affects the Secretion Pathways of Beta and Intestinal Endocrine Cells. Exp. Cell Res..

[18] Choi J.-H., Oh S.-W., Kang M.-S., Kwon H.J., Oh G.-T., Kim D.-Y. (2005). Trichostatin A Attenuates Airway Inflammation in Mouse Asthma Model. Clin. Exp. Allergy.

[19] Han S.-B., Lee J.K. (2009). Anti-Inflammatory Effect of Trichostatin-A on Murine Bone Marrow-Derived Macrophages. Arch. Pharm. Res..

[20] Ling T., Xie J. (2020). Trichostatin A Exerts Anti-Inflammation Functions in LPS-Induced Acute Lung Injury Model through Inhibiting TNF-α and Upregulating MicorRNA-146a Expression. Eur. Rev. Med. Pharmacol. Sci..

[21] Sato T., Kotake D., Hiratsuka M., Hirasawa N. (2013). Enhancement of Inflammatory Protein Expression and Nuclear Factor Κb (NF-Κb) Activity by Trichostatin A (TSA) in OP9 Preadipocytes. PLoS ONE.

[22] Zhang Q., Yang F., Li X., Wang L., Chu X., Zhang H., Gong Z. (2016). Trichostatin A Inhibits Inflammation in Phorbol Myristate Acetate-Induced Macrophages by Regulating the Acetylation of Histone and/or Non-Histone Proteins. Mol. Med. Rep..

[23] Ahn S.-G. (2011). The Histone Deacetylase Inhibitor, Trichostatin A, Induces G2/M Phase Arrest and Apoptosis in YD-10B Oral Squamous Carcinoma Cells. Oncol. Rep..

[24] Alao J.P., Stavropoulou A.V., Lam E.W., Coombes R.C. (2006). Role of Glycogen Synthase Kinase 3 Beta (GSK3β) in Mediating the Cytotoxic Effects of the Histone Deacetylase Inhibitor Trichostatin A (TSA) in MCF-7 Breast Cancer Cells. Mol. Cancer.

[25] Choi Y. (2005). Induction of Apoptosis by Trichostatin A, a Histone Deacetylase Inhibitor, Is Associated with Inhibition of Cyclooxygenase-2 Activity in Human Non-Small Cell Lung Cancer Cells. Int. J. Oncol..

[26] Deng Z., Liu X., Jin J., Xu H., Gao Q., Wang Y., Zhao J. (2016). Histone Deacetylase Inhibitor Trichostatin a Promotes the Apoptosis of Osteosarcoma Cells through P53 Signaling Pathway Activation. Int. J. Biol. Sci..

[27] Höring E., Podlech O., Silkenstedt B., Rota I.A., Adamopoulou E., Naumann U. (2013). The Histone Deacetylase Inhibitor Trichostatin A Promotes Apoptosis and Antitumor Immunity in Glioblastoma Cells. Anticancer Res..

[28] Hwang J.W., Kim Y.M., Hong S.H., Choi B.T., Lee W.H., Choi Y.H. (2005). Modulacon of Cell Cycle Control by Histone Deacetylase Inhibitor Trichostatin A in A549 Human Non-Small Cell Lung Cancer Cells. J. Life Sci..

[29] Rhodes L.V., Nitschke A.M., Segar H.C., Martin E.C., Driver J.L., Elliott S., Nam S.Y., Li M., Nephew K.P., Burow M.E. (2012). The Histone Deacetylase Inhibitor Trichostatin A Alters MicroRNA Expression Profiles in Apoptosis-Resistant Breast Cancer Cells. Oncol. Rep..

[30] Emonds E. (2010). Molecular Determinants of the Antitumor Effects of Trichostatin A in Pancreatic Cancer Cells. World J. Gastroenterol..

[31] Hong Z., Han Z., Xiao M., Yang Y., Xia X., Zhou J. (2009). Microarray Study of Mechanism of Trichostatin a Inducing Apoptosis of Molt-4 Cells. J. Huazhong Univ. Sci. Technol. Med. Sci..

[32] Li G.-C., Zhang X., Pan T.-J., Chen Z., Ye Z.-Q. (2006). Histone Deacetylase Inhibitor Trichostatin A Inhibits the Growth of Bladder Cancer Cells through Induction of P21WAF1 and G1 Cell Cycle Arrest. Int. J. Urol..

[33] Bai J., Wu Y., Wang X., Liu X., Zhong K., Huang Y., Chen Y., Gao H. (2018). *In Vitro* and *in Vivo* Characterization of the Antibacterial Activity and Membrane Damage Mechanism of Quinic Acid against Staphylococcus Aureus. J. Food Saf..

[34] Bai Y., Chen Y., Chen X., Jiang J., Wang X., Wang L., Wang J., Zhang J., Gao L. (2019). Trichostatin A Activates FOXO1 and Induces Autophagy in Osteosarcoma. Arch. Med. Sci..

[35] Gao L., Sun X., Zhang Q., Chen X., Zhao T., Lu L., Zhang J., Hong Y. (2018). Histone Deacetylase Inhibitor Trichostatin A and Autophagy Inhibitor Chloroquine Synergistically Exert Anti-Tumor Activity in H-Ras Transformed Breast Epithelial Cells. Mol. Med. Rep..

[36] Eriksson I., Joosten M., Roberg K., Öllinger K. (2013). The Histone Deacetylase Inhibitor Trichostatin A Reduces Lysosomal PH and Enhances Cisplatin-Induced Apoptosis. Exp. Cell Res..

[37] Karagiannis T.C., Harikrishnan K., El-Osta A. (2005). The Histone Deacetylase Inhibitor, Trichostatin A, Enhances Radiation Sensitivity and Accumulation of GammaH2A.X. Cancer Biol. Ther..

[38] Karagiannis T.C., Smith A.J., El’Osta A. (2004). Radio-and Chemo-Sensitization of Human Erythroleukemic K562 Cells by the Histone Deacetylase Inhibitor Trichostatin A. Hell. J. Nucl. Med..

[39] Lambert I.H., Nielsen D., Stürup S. (2020). Impact of the Histone Deacetylase Inhibitor Trichostatin A on Active Uptake, Volume-Sensitive Release of Taurine, and Cell Fate in Human Ovarian Cancer Cells. Am. J. Physiol. Cell Physiol..

[40] Piacentini P., Donadelli M., Costanzo C., Moore P.S., Palmieri M., Scarpa A. (2006). Trichostatin A Enhances the Response of Chemotherapeutic Agents in Inhibiting Pancreatic Cancer Cell Proliferation. Virchows Arch..

[41] Yoon C.Y., Park M.J., Lee J.S., Lee S.C., Oh J.J., Park H., Chung C.W., Abdullajanov M.M., Jeong S.J., Hong S.K. (2011). The Histone Deacetylase Inhibitor Trichostatin A Synergistically Resensitizes a Cisplatin Resistant Human Bladder Cancer Cell Line. J. Urol..

[42] Donadelli M., Costanzo C., Beghelli S., Scupoli M.T., Dandrea M., Bonora A., Piacentini P., Budillon A., Caraglia M., Scarpa A. (2007). Synergistic Inhibition of Pancreatic Adenocarcinoma Cell Growth by Trichostatin A and Gemcitabine. Biochim. Biophys. Acta Mol. Cell Res..

[43] Murray-Zmijewski F., Lane D.P., Bourdon J.C. (2006). P53/P63/P73 Isoforms: An Orchestra of Isoforms to Harmonise Cell Differentiation and Response to Stress. Cell Death Differ..

[44] Rahman M.M., Kukita A., Kukita T., Shobuike T., Nakamura T., Kohashi O. (2003). Two Histone Deacetylase Inhibitors, Trichostatin A and Sodium Butyrate, Suppress Differentiation into Osteoclasts but Not into Macrophages. Blood.

[45] Touma S.E., Goldberg J.S., Moench P., Guo X., Tickoo S.K., Gudas L.J., Nanus D.M. (2005). Retinoic Acid and the Histone Deacetylase Inhibitor Trichostatin A Inhibit the Proliferation of Human Renal Cell Carcinoma in a Xenograft Tumor Model. Clin. Cancer Res..

[46] Januchowski R., Jagodzinski P.P. (2007). Trichostatin A Down-Regulates ZAP-70, LAT and SLP-76 Content in Jurkat T Cells. Int. Immunopharmacol..

[47] Sanaei F., Amin M.M., Alavijeh Z.P., Esfahani R.A., Sadeghi M., Bandarrig N.S., Fatehizadeh A., Taheri E., Rezakazemi M. (2021). Health Risk Assessment of Potentially Toxic Elements Intake via Food Crops Consumption: Monte Carlo Simulation-Based Probabilistic and Heavy Metal Pollution Index. Environ. Sci. Pollut. Res..

[48] Sanaei M., Kavoosi F. (2019). Effect of 5-Aza-2′-Deoxycytidine in Comparison to Valproic Acid and Trichostatin a on Histone Deacetylase 1, Dna Methyltransferase 1, and Cip/Kip Family (P21, P27, and P57) Genes Expression, Cell Growth Inhibition, and Apoptosis Induction in Colon Cancer Sw480 Cell Line. Adv. Biomed. Res..

[49] Vincent A., Ducourouble M., Van Seuningen I. (2008). Epigenetic Regulation of the Human Mucin Gene *MUC4* in Epithelial Cancer Cell Lines Involves Both DNA Methylation and Histone Modifications Mediated by DNA Methyltransferases and Histone Deacetylases. FASEB j..

[50] Geng Y., Liu J., Xie Y., Jiang H., Zuo K., Li T., Liu Z. (2018). Trichostatin A Promotes GLI1 Degradation and P21 Expression in Multiple Myeloma Cells. Cancer Manag. Res..

[51] Alao J.P., Lam E.W.-F., Ali S., Buluwela L., Bordogna W., Lockey P., Varshochi R., Stavropoulou A.V., Coombes R.C., Vigushin D.M. (2004). Histone Deacetylase Inhibitor Trichostatin A Represses Estrogen Receptor α-Dependent Transcription and Promotes Proteasomal Degradation of Cyclin D1 in Human Breast Carcinoma Cell Lines. Clin. Cancer Res..

[52] Sassi F.d.A., Caesar L., Jaeger M., Nör C., Abujamra A.L., Schwartsmann G., de Farias C.B., Brunetto A.L., Lopez P.L.d.C., Roesler R. (2014). Inhibitory Activities of Trichostatin A in U87 Glioblastoma Cells and Tumorsphere-Derived Cells. J. Mol. Neurosci..

[53] Aziee S., Haiyuni M.Y., Al-Jamal H.A.N., Shafini M.Y., Wahab R.A., Shamsuddin S., Johan M.F. (2018). Apoptotic Induction in CCRF-CEM and HL-60 Human Leukemic Cell Lines by 5-Azacitidine and Trichostatin A. J. Biomed. Clin. Sci..

[54] Balaguer T.M., Gómez-Martínez A., García-Morales P., Lacueva J., Calpena R., Reverte L.R., Riquelme N.L., Martinez-Lacaci I., Ferragut J.A., Saceda M. (2012). Dual Regulation of P-Glycoprotein Expression by Trichostatin A in Cancer Cell Lines. BMC Mol. Biol..

[55] Buishand F.O., Cardin E., Hu Y., Ried T. (2018). Trichostatin A Preferentially Reverses the Upregulation of Gene-Expression Levels Induced by Gain of Chromosome 7 in Colorectal Cancer Cell Lines. Genes Chromosomes Cancer.

[56] Cecconi D., Donadelli M., Rinalducci S., Zolla L., Scupoli M.T., Scarpa A., Palmieri M., Righetti P.G. (2007). Proteomic Analysis of Pancreatic Endocrine Tumor Cell Lines Treated with the Histone Deacetylase Inhibitor Trichostatin A. Proteomics.

[57] Chen C., Chen C., Chen J., Zhou L., Xu H., Jin W., Wu J., Gao S. (2013). Histone Deacetylases Inhibitor Trichostatin A Increases the Expression of Dleu2/MiR-15a/16-1 via HDAC3 in Non-Small Cell Lung Cancer. Mol. Cell Biochem..

[58] Chen Z., Yang Y., Liu B., Wang B., Sun M., Zhang L., Chen B., You H., Zhou M. (2015). Promotion of Metastasis-Associated Gene Expression in Survived PANC-1 Cells Following Trichostatin A Treatment. Anti-Cancer Agents Med. Chem..

[59] Cheng D.-D., Yang Q.-C., Zhang Z.-C., Yang C.-X., Liu Y.-W. (2012). Antitumor Activity of Histone Deacetylase Inhibitor Trichostatin A in Osteosarcoma Cells. Asian Pac. J. Cancer Prev..

[60] Chiba T., Yokosuka O., Fukai K., Kojima H., Tada M., Arai M., Imazeki F., Saisho H. (2004). Cell Growth Inhibition and Gene Expression Induced by the Histone Deacetylase Inhibitor, Trichostatin A, on Human Hepatoma Cells. Oncology.

[61] Chodkowska A., Bieńkowska A., S\lyk Ż., Giebu\ltowicz J., Ma\lecki M. (2020). Anticancer Activity of Topical Ointments with Histone Deacetylase Inhibitor, Trichostatin A. Adv. Clin. Exp. Med..

[62] de Oliveira Santos J., Zuma A.A., de Luna Vitorino F.N., da Cunha J.P.C., de Souza W., Motta M.C.M. (2019). Trichostatin A Induces *Trypanosoma Cruzi* Histone and Tubulin Acetylation: Effects on Cell Division and Microtubule Cytoskeleton Remodelling. Parasitology.

[63] Diao J.-S., Xia W.-S., Yi C.-G., Wang Y.-M., Li B., Xia W., Liu B., Guo S.-Z., Sun X.-D. (2011). Trichostatin A Inhibits Collagen Synthesis and Induces Apoptosis in Keloid Fibroblasts. Arch. Dermatol. Res..

[64] Drzewiecka H., Jagodzinski P.P. (2012). Trichostatin A Reduced Phospholipase C Gamma-1 Transcript and Protein Contents in MCF-7 Breast Cancer Cells. Biomed. Pharmacother..

[65] Han M.H., Park C., Kwon T.K., Kim G.-Y., Kim W.-J., Hong S.H., Yoo Y.H., Choi Y.H. (2015). The Histone Deacetylase Inhibitor Trichostatin A Sensitizes Human Renal Carcinoma Cells to TRAIL-Induced Apoptosis through Down-Regulation of c-FLIPL. Biomol. Ther..

[66] He J., Liu H., Chen Y. (2006). Effects of Trichostatin A on HDAC8 Expression, Proliferation and Cell Cycle of Molt-4 Cells. J. Huazhong Univ. Sci. Technol..

[67] Hong S., Chang S.-Y., Yeom D.-H., Kang J.-H., Hong K.-J. (2007). Differential Regulation of Thrombospondin-1 Expression and Antiangiogenesis of ECV304 Cells by Trichostatin A and Helixor A. Anti-Cancer Drugs.

[68] Hrgovic I., Doll M., Kleemann J., Wang X.-F., Zoeller N., Pinter A., Kippenberger S., Kaufmann R., Meissner M. (2016). The Histone Deacetylase Inhibitor Trichostatin a Decreases Lymphangiogenesis by Inducing Apoptosis and Cell Cycle Arrest via P21-Dependent Pathways. BMC Cancer.

[69] Hsu Y.-F., Sheu J.-R., Hsiao G., Lin C.-H., Chang T.-H., Chiu P.-T., Wang C.-Y., Hsu M.-J. (2011). P53 in Trichostatin A Induced C6 Glioma Cell Death. Biochim. Biophys. Acta Gen. Subj..

[70] Zhang X.F., Yan Q., Shen W., Gurunathan S. (2016). Trichostatin A enhances the apoptotic potential of palladium nanoparticles in human cervical cancer cells. Int. J. Mol. Sci..

[71] Huang K., Liu Y., Gu C., Liu D., Zhao B. (2020). Trichostatin A Augments Esophageal Squamous Cell Carcinoma Cells Migration by Inducing Acetylation of RelA at K310 Leading Epithelia–Mesenchymal Transition. Anti-Cancer Drugs.

[72] Huang X.-Y., Xiao G.-T., Huang T.-X., Chen Z.-X., Gao W.-Y., Zheng B.-Y., Wang X. (2021). Trichostatin A Alleviates HBx-Induced HCC Metastasis in Metabolic Stress through Up-Regulating SIRT3 Expression; In Review. https://assets.researchsquare.com/files/rs-420738/v1/5af26051-9733-43fd-810f-6817798dfef0.pdf?c=1631882363.

[73] Kang J.-H., Kim S.-A., Chang S.-Y., Hong S., Hong K.-J. (2007). Inhibition of Trichostatin A-Induced Antiangiogenesis by Small-Interfering RNA for Thrombospondin-1. Exp. Mol. Med..

[74] Kashiwagi Y., Horie K., Kanno C., Inomata M., Imamura T., Kato M., Yamamoto T., Yamashita H. (2010). Trichostatin A–Induced TGF-β Type II Receptor Expression in Retinoblastoma Cell Lines. Invest. Ophthalmol. Vis. Sci..

[75] Katsura T., Iwai S., Ota Y., Shimizu H., Ikuta K., Yura Y. (2009). The Effects of Trichostatin A on the Oncolytic Ability of Herpes Simplex Virus for Oral Squamous Cell Carcinoma Cells. Cancer Gene Ther..

[76] Kim Y.B., Yoshida M., Horinouchi S. (1999). Selective Induction of Cyclin-Dependent Kinase Inhibitors and Their Roles in Cell Cycle Arrest Caused by Trichostatin A, an Inhibitor of Histone Deacetylase. Ann. N. Y. Acad Sci..

[77] Yoo Y.C., Lee H.Y., Kwak S.T., Lee K.B. Regulatory Effect of Chondroitin Sulfates Derived Form Human Placenta on Mitogen-Induced Activation of Murine Splenocytes. Proceedings of the PSK Conference.

[78] Kim H.-N., Ha H., Lee J.-H., Jung K., Yang D., Woo K.M., Lee Z.H. (2009). Trichostatin A Inhibits Osteoclastogenesis and Bone Resorption by Suppressing the Induction of C-Fos by RANKL. Eur. J. Pharmacol..

[79] Li H., Wu X. (2004). Histone Deacetylase Inhibitor, Trichostatin A, Activates P21WAF1/CIP1 Expression through Downregulation of c-Myc and Release of the Repression of c-Myc from the Promoter in Human Cervical Cancer Cells. Biochem. Biophys. Res. Commun..

[80] Li C., Tao Y., Li C., Liu B., Liu J., Wang G., Liu H. (2016). PU.1-Bim Axis Is Involved in Trichostatin A-Induced Apoptosis in Murine pro-B Lymphoma FL5.12 Cells. Acta Biochim. Biophys. Sin..

[81] Liu Y.-W., Wang S.-A., Hsu T.-Y., Chen T.-A., Chang W.-C., Hung J.-J. (2010). Inhibition of LPS-Induced C/EBPδ by Trichostatin a Has a Positive Effect on LPS-Induced Cyclooxygenase 2 Expression in RAW264.7 Cells. J. Cell. Biochem..

[82] Liu Y., He G., Wang Y., Guan X., Pang X., Zhang B. (2013). MCM-2 Is a Therapeutic Target of Trichostatin A in Colon Cancer Cells. Toxicol. Lett..

[83] Liu J., Li Y., Dong F., Li L., Masuda T., Allen T.D., Lobe C.G. (2015). Trichostatin A Suppresses Lung Adenocarcinoma Development in Grg1 Overexpressing Transgenic Mice. Biochem. Biophys. Res. Commun..

[84] Liu J.-H., Cao Y.-M., Rong Z.-P., Ding J., Pan X. (2021). Trichostatin A Induces Autophagy in Cervical Cancer Cells by Regulating the PRMT5-STC1-TRPV6-JNK Pathway. Pharmacology.

[85] Łuczak M.W., Jagodziński P.P. (2009). Trichostatin A Down-Regulates CYP19 Transcript and Protein Levels in MCF-7 Breast Cancer Cells. Biomed. Pharmacother..

[86] Ma J., Guo X., Zhang S., Liu H., Lu J., Dong Z., Liu K., Ming L. (2015). Trichostatin A, a Histone Deacetylase Inhibitor, Suppresses Proliferation and Promotes Apoptosis of Esophageal Squamous Cell Lines. Mol. Med. Rep..

[87] Mazzio E.A., Soliman K.F.A. (2018). Whole-Transcriptomic Profile of SK-MEL-3 Melanoma Cells Treated with the Histone Deacetylase Inhibitor: Trichostatin A. Cancer Genom. Proteom..

[88] Meng C., Dai D., Guo K. (2009). Comparative Evaluation of the Effects of 5-Aza-2′-Deoxycytidine and Trichostatin A on Reactivation of HMLH1 in COC1/DDP Ovarian Cancer Cell Line. Chin. J. Cancer Res..

[89] Meng F., Sun G., Zhong M., Yu Y., Brewer M.A. (2013). Anticancer Efficacy of Cisplatin and Trichostatin A or 5-Aza-2′-Deoxycytidine on Ovarian Cancer. Br. J. Cancer.

[90] Miyanaga A., Gemma A., Noro R., Kataoka K., Matsuda K., Nara M., Okano T., Seike M., Yoshimura A., Kawakami A. (2008). Antitumor Activity of Histone Deacetylase Inhibitors in Non-Small Cell Lung Cancer Cells: Development of a Molecular Predictive Model. Mol. Cancer Ther..

[91] Moore P.S., Barbi S., Donadelli M., Costanzo C., Bassi C., Palmieri M., Scarpa A. (2004). Gene Expression Profiling after Treatment with the Histone Deacetylase Inhibitor Trichostatin A Reveals Altered Expression of Both Pro- and Anti-Apoptotic Genes in Pancreatic Adenocarcinoma Cells. Biochim. Biophys. Acta Mol. Cell Res..

[92] Moreira J.M.A., Scheipers P., Sørensen P. (2003). The Histone Deacetylase Inhibitor Trichostatin A Modulates CD4+ T Cell Responses. BMC Cancer.

[93] Mukhopadhyay N.K., Weisberg E., Gilchrist D., Bueno R., Sugarbaker D.J., Jaklitsch M.T. (2006). Effectiveness of Trichostatin A as a Potential Candidate for Anticancer Therapy in Non–Small-Cell Lung Cancer. Ann. Thorac. Surg..

[94] Nagamine M.K., Sanches D.S., Pinello K.C., Torres L.N., Mennecier G., Latorre A.O., Fukumasu H., Dagli M.L.Z. (2011). *In Vitro* Inhibitory Effect of Trichostatin A on Canine Grade 3 Mast Cell Tumor. Vet. Res. Commun..

[95] Nagaraja S.S., Krishnamoorthy V., Raviraj R., Paramasivam A., Nagarajan D. (2017). Effect of Trichostatin A on Radiation Induced Epithelial-Mesenchymal Transition in A549 Cells. Biochem. Biophys. Res. Commun..

[96] Noh E.J., Lim D.-S., Jeong G., Lee J.-S. (2009). An HDAC Inhibitor, Trichostatin A, Induces a Delay at G2/M Transition, Slippage of Spindle Checkpoint, and Cell Death in a Transcription-Dependent Manner. Biochem. Biophys. Res. Commun..

[97] Noh H., Park J., Shim M., Lee Y. (2016). Trichostatin A Enhances Estrogen Receptor-Alpha Repression in MCF-7 Breast Cancer Cells under Hypoxia. Biochem. Biophys. Res. Commun..

[98] Olaharski A.J., Ji Z., Woo J.-Y., Lim S., Hubbard A.E., Zhang L., Smith M.T. (2006). The Histone Deacetylase Inhibitor Trichostatin A Has Genotoxic Effects in Human Lymphoblasts *In Vitro*. Toxicol. Sci..

[99] Pang X., He G., Luo C., Wang Y., Zhang B. (2016). Knockdown of Rad9A Enhanced DNA Damage Induced by Trichostatin A in Esophageal Cancer Cells. Tumor. Biol..

[100] Papeleu P., Loyer P., Vanhaecke T., Elaut G., Geerts A., Guguen-Guillouzo C., Rogiers V. (2003). Trichostatin A Induces Differential Cell Cycle Arrests but Does Not Induce Apoptosis in Primary Cultures of Mitogen-Stimulated Rat Hepatocytes. J. Hepatol..

[101] Park S.-J., Kim M.-J., Kim H.-B., Sohn H.-Y., Bae J.-H., Kang C.-D., Kim S.-H. (2009). Trichostatin A Sensitizes Human Ovarian Cancer Cells to TRAIL-Induced Apoptosis by down-Regulation of c-FLIPL via Inhibition of EGFR Pathway. Biochem. Pharmacol..

[102] Peiffer L., Poll-Wolbeck S.J., Flamme H., Gehrke I., Hallek M., Kreuzer K.-A. (2014). Trichostatin A Effectively Induces Apoptosis in Chronic Lymphocytic Leukemia Cells via Inhibition of Wnt Signaling and Histone Deacetylation. J. Cancer Res. Clin. Oncol..

[103] Platta C.S., Greenblatt D.Y., Kunnimalaiyaan M., Chen H. (2007). The HDAC Inhibitor Trichostatin A Inhibits Growth of Small Cell Lung Cancer Cells. J. Surg. Res..

[104] Ruan W.-M., Li Y.-L., Nie G., Zhou W.-X., Zou X.-M. (2014). Differential Expression of Glycoprotein Non-Metastatic Melanoma Protein B (GPNMB) Involved in Trichostatin A-Induced Apoptosis in Gastric Cancer. Int. J. Clin. Exp. Med..

[105] Salvi V., Bosisio D., Mitola S., Andreoli L., Tincani A., Sozzani S. (2010). Trichostatin A Blocks Type I Interferon Production by Activated Plasmacytoid Dendritic Cells. Immunobiology.

[106] Sanaei M., Kavoosi F., Arabloo M. (2020). Effect of Curcumin in Comparison with Trichostatin A on the Reactivation of Estrogen Receptor Alpha Gene Expression, Cell Growth Inhibition and Apoptosis Induction in Hepatocellular Carcinoma Hepa 1-6 Cell LLine. Asian Pac. J. Cancer Prev..

[107] Seo J., Cho N., Kim H., Tsurumi T., Jang Y., Lee W., Lee S. (2008). Cell Cycle Arrest and Lytic Induction of EBV-Transformed B Lymphoblastoid Cells by a Histone Deacetylase Inhibitor, Trichostatin A. Oncol. Rep..

[108] Sharma P., Kumar S., Kundu G.C. (2010). Transcriptional Regulation of Human Osteopontin Promoter by Histone Deacetylase Inhibitor, Trichostatin A in Cervical Cancer Cells. Mol. Cancer.

[109] Sharma A., Bhat A.A., Krishnan M., Singh A.B., Dhawan P. (2013). Trichostatin-A Modulates Claudin-1 MRNA Stability through the Modulation of Hu Antigen R and Tristetraprolin in Colon Cancer Cells. Carcinogenesis.

[110] Shen Z., Liao X., Shao Z., Feng M., Yuan J., Wang S., Gan S., Ha Y., He Z., Jie W. (2019). Short-Term Stimulation with Histone Deacetylase Inhibitor Trichostatin a Induces Epithelial-Mesenchymal Transition in Nasopharyngeal Carcinoma Cells without Increasing Cell Invasion Ability. BMC Cancer.

[111] Shindo Y., Arai W., Konno T., Kohno T., Kodera Y., Chiba H., Miyajima M., Sakuma Y., Watanabe A., Kojima T. (2021). Effects of Histone Deacetylase Inhibitors Tricostatin A and Quisinostat on Tight Junction Proteins of Human Lung Adenocarcinoma A549 Cells and Normal Lung Epithelial Cells. Histochem. Cell Biol..

[112] Song W.-Y., Yang Q.-L., Zhao W.-L., Jin H.-X., Yao G.-D., Peng Z.-F., Shi S.-L., Yang H.-Y., Zhang X.-Y., Sun Y.-P. (2016). The Effects of Anticancer Drugs TSA and GSK on Spermatogenesis in Male Mice. Am. J. Transl. Res..

[113] Strait K.A., Dabbas B., Hammond E.H., Warnick C.T., Ilstrup S.J., Ford C.D. (2002). Cell Cycle Blockade and Differentiation of Ovarian Cancer Cells by the Histone Deacetylase Inhibitor Trichostatin A Are Associated with Changes in P21, Rb, and Id Proteins 1 Supported by Grants from Feature Films for Families Cancer Research Fund (CEO, Forrest S. Baker III) and The Deseret Foundation. 1. Mol. Cancer Ther..

[114] Subramanian C., Jarzembowski J.A., Opipari A.W., Castle V.P., Kwok R.P.S. (2007). CREB-Binding Protein Is a Mediator of Neuroblastoma Cell Death Induced By the Histone Deacetylase Inhibitor Trichostatin A. Neoplasia.

[115] Sun C., Liu X., Chen Y., Liu F. (2006). Anticancer Activities of Trichostatin A on Malignant Lymphoid Cells. J. Huazhong Univ. Sci. Technol..

[116] Sun S., Han Y., Liu J., Fang Y., Tian Y., Zhou J., Ma D., Wu P. (2014). Trichostatin A Targets the Mitochondrial Respiratory Chain, Increasing Mitochondrial Reactive Oxygen Species Production to Trigger Apoptosis in Human Breast Cancer Cells. PLoS ONE.

[117] Tarnowski M., Tkacz M., Kopytko P., Bujak J., Piotrowska K., Pawlik A. (2019). Trichostatin A Inhibits Rhabdomyosarcoma Proliferation and Induces Differentiation through MyomiR Reactivation. Folia Biol..

[118] Tavakoli-Yaraki M., Karami-Tehrani F., Salimi V., Sirati-Sabet M. (2013). Induction of Apoptosis by Trichostatin A in Human Breast Cancer Cell Lines: Involvement of 15-Lox-1. Tumor. Biol..

[119] Toki S., Goleniewska K., Reiss S., Zhou W., Newcomb D.C., Bloodworth M.H., Stier M.T., Boyd K.L., Polosukhin V.V., Subramaniam S. (2016). The Histone Deacetylase Inhibitor Trichostatin A Suppresses Murine Innate Allergic Inflammation by Blocking Group 2 Innate Lymphoid Cell (ILC2) Activation. Thorax.

[120] Urbinati G., Marsaud V., Nicolas V., Vergnaud-Gauduchon J., Renoir J.-M. (2011). Liposomal Trichostatin A: Therapeutic Potential in Hormone-Dependent and -Independent Breast Cancer Xenograft Models. Horm. Mol. Biol. Clin. Investig..

[121] Vigushin D.M., Ali S., Pace P.E., Mirsaidi N., Ito K., Adcock I., Coombes R.C. (2001). Trichostatin A Is a Histone Deacetylase Inhibitor with Potent Antitumor Activity against Breast Cancer *in Vivo*. Clin. Cancer Res..

[122] Kang F.-W., Que L., Wu M., Wang Z.-L., Sun J. (2012). Effects of Trichostatin A on HIF-1α and VEGF Expression in Human Tongue Squamous Cell Carcinoma Cells *in Vitro*. Oncol. Rep..

[123] Wang F., Qi Y., Li X., He W., Fan Q.-X., Zong H. (2013). HDAC Inhibitor Trichostatin A Suppresses Esophageal Squamous Cell Carcinoma Metastasis through HADC2 Reduced MMP-2/9. Clin. Investig. Med..

[124] Wang X., Xu J., Wang H., Wu L., Yuan W., Du J., Cai S. (2015). Trichostatin A, a Histone Deacetylase Inhibitor, Reverses Epithelial–Mesenchymal Transition in Colorectal Cancer SW480 and Prostate Cancer PC3 Cells. Biochem. Biophys. Res. Commun..

[125] Wang S.-C., Wang S.-T., Liu H.-T., Wang X.-Y., Wu S.-C., Chen L.-C., Liu Y.-W. (2017). Trichostatin A Induces Bladder Cancer Cell Death via Intrinsic Apoptosis at the Early Phase and Sp1-Survivin Downregulation at the Late Phase of Treatment. Oncol. Rep..

[126] Woo H.J., Choi Y.H. (2006). G1 Phase Arrest of the Cell Cycle by Histone Deacetylase Inhibitor Trichostatin A in U937 Human Leukemic Cells. J. Cancer Prev..

[127] Wu Y., Guo S.-W. (2006). Inhibition of Proliferation of Endometrial Stromal Cells by Trichostatin A, RU486, CDB-2914, N-Acetylcysteine, and ICI 182780. Gynecol. Obs. Invest..

[128] Xingang L., Weikai C., Junxia G., Guohui C., Yan C. (2004). Regulation of Histone Acetylation and Apoptosis by Trichostatin in HL-60 Cells. J. Huazhong Univ. Sci. Technol. Med. Sci..

[129] Xu Z., Wang Y., Mei Q., Chen J., Du J., Wei Y., Xu Y. (2006). Trichostatin A Inhibits Proliferation, Induces Apoptosis and Cell Cycle Arrest in HeLa Cells. Chin. J. Cancer Res..

[130] Yang D.-H., Lee J.-W., Lee J., Moon E.-Y. (2014). Dynamic Rearrangement of F-Actin Is Required to Maintain the Antitumor Effect of Trichostatin A. PLoS ONE.

[131] Yi T., Baek J.-H., Kim H.-J., Choi M.-H., Seo S.-B., Ryoo H.-M., Kim G.-S., Woo K.M. (2007). Trichostatin A-Mediated Upregulation of P21WAF1 Contributes to Osteoclast Apoptosis. Exp. Mol. Med..

[132] You B.R., Park W.H. (2013). Trichostatin A Induces Apoptotic Cell Death of HeLa Cells in a Bcl-2 and Oxidative Stress-Dependent Manner. Int. J. Oncol..

[133] ZHANG X., YU X., ZHAO M., YI X., DU Z., XU Y. (2007). Trichostatin A Induces Mitotic Catastrophe of Prostate Cancer DU145 Cells. Chin. J. Cancer Biother..

[134] Zhang S., Cai X., Huang F., Zhong W., Yu Z. (2008). Effect of Trichostatin A on Viability and Microrna Expression in Human Pancreatic Cancer Cell Line Bxpc-3. Exp. Oncol..

[135] Zhang C.Z.Y., Zhang H.T., Chen G.G., Lai P.B.S. (2011). Trichostatin A Sensitizes HBx-Expressing Liver Cancer Cells to Etoposide Treatment. Apoptosis.

[136] Zhang Q.-C., Jiang S.-J., Zhang S., Ma X.-B. (2012). Histone Deacetylase Inhibitor Trichostatin A Enhances Antitumor Effects of Docetaxel or Erlotinib in A549 Cell Line. Asian Pac. J. Cancer Prev..

[137] Zhang X., Jiang S.-J., Shang B., Jiang H.-J. (2015). Effects of Histone Deacetylase Inhibitor Trichostatin A Combined with Cisplatin on Apoptosis of A549 Cell Line: TSA Combined with Cisplatin. Thorac. Cancer.

[138] Zhang F., Shao C., Chen Z., Li Y., Jing X., Huang Q. (2021). Low Dose of Trichostatin a Improves Radiation Resistance by Activating Akt/Nrf2-Dependent Antioxidation Pathway in Cancer Cells. Radiat. Res..

[139] ZHU Y., PAN M., WEI Q., CAO X. (2007). A Study on Transcription Regulation Induced by Trichostatin A during Cytotoxicity on MCF-7 Cells. Acta Univ. Med. Nanjing Nat. Sci..

[140] Zohre S., Kazem N.-K., Abolfazl A., Mohammad R.-Y., Aliakbar M., Effat A., Zahra D., Hassan D., Nosratollah Z. (2014). Trichostatin A-Induced Apoptosis Is Mediated by Krüppel-like Factor 4 in Ovarian and Lung Cancer. Asian Pac. J. Cancer Prev..

[141] Bajbouj K., Mawrin C., Hartig R., Schulze-Luehrmann J., Wilisch-Neumann A., Roessner A., Schneider-Stock R. (2012). P53-Dependent Antiproliferative and pro-Apoptotic Effects of Trichostatin A (TSA) in Glioblastoma Cells. J. Neuro. Oncol..

[142] Wetzel M., Premkumar D.R., Arnold B., Pollack I.F. (2005). Effect of Trichostatin A, a Histone Deacetylase Inhibitor, on Glioma Proliferation in Vitro by Inducing Cell Cycle Arrest and Apoptosis. J. Neurosurg. Pediatr..

[143] Foltz G., Yoon J.-G., Lee H., Ma L., Tian Q., Hood L., Madan A. (2010). Epigenetic Regulation of Wnt Pathway Antagonists in Human Glioblastoma Multiforme. Genes Cancer.

[144] Chen J., Kang J. (2005). Quercetin and Trichostatin A Cooperatively Kill Human Leukemia Cells. Die Pharm. Int. J. Pharm. Sci..

[145] Bouyahya A., Mechchate H., Oumeslakht L., Zeouk I., Aboulaghras S., Balahbib A., El Omari N. (2022). The role of epigenetic modifications in human cancers and the use of natural compounds as epidrugs: Mechanistic pathways and pharmacodynamic actions. Biomolecules.

[146] Park I.-H., Kang J.-H., Shin J.-M., Lee H.-M. (2016). Trichostatin A Inhibits Epithelial Mesenchymal Transition Induced by TGF-Β1 in Airway Epithelium. PLoS ONE.

[147] Liu D., Liu Y., Qi B., Gu C., Huo S., Zhao B. (2021). Trichostatin A Promotes Esophageal Squamous Cell Carcinoma Cell Migration and EMT through BRD4/ERK1/2-dependent Pathway. Cancer Med..

[148] Dai L., He G., Zhang K., Guan X., Wang Y., Zhang B. (2018). Trichostatin A Induces P53-dependent Endoplasmic Reticulum Stress in Human Colon Cancer Cells. Oncol. Lett..

[149] Han R.-F., Li K., Yang Z.-S., Chen Z.-G., Yang W.-C. (2014). Trichostatin A Induces Mesenchymal-like Morphological Change and Gene Expression but Inhibits Migration and Colony Formation in Human Cancer Cells. Mol. Med. Rep..

[150] Pei Y., Robertson E.S. (2020). The Crosstalk of Epigenetics and Metabolism in Herpesvirus Infection. Viruses.

[151] Park H., Lee Y.J., Kim T.H., Lee J., Yoon S., Choi W.S., Myung C.-S., Kim H.S. (2008). Effects of Trichostatin A, a Histone Deacetylase Inhibitor, on the Regulation of Apoptosis in H-Ras-Transformed Breast Epithelial Cells. Int. J. Mol. Med..

[152] Saito Y., Jones P.M. (2006). Epigenetic Activation of Tumor Suppressor MicroRNAs in Human Cancer Cells. Cell Cycle.

[153] Wang X., Chen S., Shen T., Lu H., Xiao D., Zhao M., Yao Y., Li X., Zhang G., Zhou X. (2020). Trichostatin A Reverses Epithelial-mesenchymal Transition and Attenuates Invasion and Migration in MCF-7 Breast Cancer Cells. Exp. Ther. Med..

[154] Xiong J., Xu X., Zhou X., Liu J., Gong Z., Wu P., Li W. (2014). USP22 Transcriptional Activity Is Negatively Regulated by the Histone Deacetylase Inhibitor Trichostatin A. Mol. Med. Rep..

[155] Zhang J., Xia Y., Sun J. (2021). Breast and Gut Microbiome in Health and Cancer. Genes Dis..

[156] Kong W.Y., Yee Z.Y., Mai C.W., Fang C.-M., Abdullah S., Ngai S.C. (2019). Zebularine and Trichostatin A Sensitized Human Breast Adenocarcinoma Cells towards Tumor Necrosis Factor-Related Apoptosis Inducing Ligand (TRAIL)-Induced Apoptosis. Heliyon.

[157] Tan L., Kwok R.P., Shukla A., Kshirsagar M., Zhao L., Opipari A.W., Liu J.R. (2011). Trichostatin A Restores Apaf-1 Function in Chemoresistant Ovarian Cancer Cells. Cancer.

[158] Wu J., Hu C., Gu Q., Li Y., Song M. (2010). Trichostatin A Sensitizes Cisplatin-Resistant A549 Cells to Apoptosis by up-Regulating Death-Associated Protein Kinase. Acta Pharm. Sin..

[159] Lin W.-C., Hsu F.-S., Kuo K.-L., Liu S.-H., Shun C.-T., Shi C.-S., Chang H.-C., Tsai Y.-C., Lin M.-C., Wu J.-T. (2018). Trichostatin A, a Histone Deacetylase Inhibitor, Induces Synergistic Cytotoxicity with Chemotherapy via Suppression of Raf/MEK/ERK Pathway in Urothelial Carcinoma. J. Mol. Med..

[160] Hajji N., Wallenborg K., Vlachos P., Nyman U., Hermanson O., Joseph B. (2008). Combinatorial Action of the HDAC Inhibitor Trichostatin A and Etoposide Induces Caspase-Mediated AIF-Dependent Apoptotic Cell Death in Non-Small Cell Lung Carcinoma Cells. Oncogene.

[161] Jasek E., Lis G.J., Jasińska M., Jurkowska H., Litwin J.A. (2012). Effect of Histone Deacetylase Inhibitors Trichostatin A and Valproic Acid on Etoposide-Induced Apoptosis in Leukemia Cells. Anticancer Res..

[162] Fandy T.E., Srivastava R.K. (2006). Trichostatin A Sensitizes TRAIL-Resistant Myeloma Cells by Downregulation of the Antiapoptotic Bcl-2 Proteins. Cancer Chemother. Pharm..

[163] Zhang X., Yashiro M., Ren J., Hirakawa K. (2006). Histone Deacetylase Inhibitor, Trichostatin A, Increases the Chemosensitivity of Anticancer Drugs in Gastric Cancer Cell Lines. Oncol. Rep..

[164] Jang E.R., Lim S.-J., Lee E.S., Jeong G., Kim T.-Y., Bang Y.-J., Lee J.-S. (2004). The Histone Deacetylase Inhibitor Trichostatin A Sensitizes Estrogen Receptor α-Negative Breast Cancer Cells to Tamoxifen. Oncogene.

[165] Sato H., Kashiba T., Uzu M., Fujiwara T., Shibata Y., Suzuki R., Yamaura K., Hisaka A. (2015). Combined Treatment of Trichostatin A Enhances Cytotoxic Effects of Sunitinib on Renal Cell Carcinoma Cells. Am. Assoc. Cancer Res..

[166] Sato H., Uzu M., Kashiba T., Fujiwara T., Hatakeyama H., Ueno K., Hisaka A. (2019). Trichostatin A Modulates Cellular Metabolism in Renal Cell Carcinoma to Enhance Sunitinib Sensitivity. Eur. J. Pharmacol..

[167] Donia T., Khedr S., Salim E.I., Hessien M. (2021). Trichostatin A Sensitizes Hepatoma Cells to Taxol More than 5-Aza-DC and Dexamethasone. Drug Metab. Pers. Ther..

[168] Kim J.H., Shin J.H., Chie E.K., Wu H.-G., Kim J.S., Kim I.H., Ha S.W., Park C.I., Kang W.-S. (2004). Trichostatin A, a Histone Deacetylase Inhibitor, Potentiated Cytotoxic Effect of Ionizing Radiation in Human Head and Neck Cancer Cell Lines. Radiat. Oncol. J..

[169] Kim I.A., Kim J.H., Shin J.H., Kim I.H., Kim J.S., Wu H.-G., Chie E.K., Kim Y.H., Kim B.-K., Hong S. (2005). A Histone Deacetylase Inhibitor, Trichostatin A, Enhances Radiosensitivity by Abrogating G2/M Arrest in Human Carcinoma Cells. Cancer Res. Treat..

[170] Igaz N., Kovács D., Rázga Z., Kónya Z., Boros I.M., Kiricsi M. (2016). Modulating Chromatin Structure and DNA Accessibility by Deacetylase Inhibition Enhances the Anti-Cancer Activity of Silver Nanoparticles. Colloids Surf. B Biointerfaces.

[171] Shin S., Kim M., Lee S.-J., Park K.-S., Lee C.H. (2017). Trichostatin A Sensitizes Hepatocellular Carcinoma Cells to Enhanced NK Cell-Mediated Killing by Regulating Immune-Related Genes. Cancer Genom. Proteom..

[172] Roh M.S., Kim C.W., Park B.S., Kim G.C., Jeong J.H., Kwon H.C., Suh D.J., Cho K.H., Yee S.-B., Yoo Y.H. (2004). Mechanism of Histone Deacetylase Inhibitor Trichostatin A Induced Apoptosis in Human Osteosarcoma Cells. Apoptosis.

[173] Lin L., Wei Y., Zhu W., Wang C., Su R., Feng H., Yang H. (2018). Prevalence, Risk Factors and Associated Adverse Pregnancy Outcomes of Anaemia in Chinese Pregnant Women: A Multicentre Retrospective Study. BMC Pregnancy Childbirth.

[174] Zhang F., Zhang T., Teng Z., Zhang R., Wang J.-B., Mei Q.-B. (2009). Sensitization to γ-Irradiation-Induced Cell Cycle Arrest and Apoptosis by the Histone Deacetylase Inhibitor Trichostatin A in Non-Small Cell Lung Cancer (NSCLC) Cells. Cancer Biol. Ther..

[175] Chan S.-T., Yang N.-C., Huang C.-S., Liao J.-W., Yeh S.-L. (2013). Quercetin Enhances the Antitumor Activity of Trichostatin A through Upregulation of P53 Protein Expression *In Vitro* and In Vivo. PLoS ONE.

[176] Chan S.-T., Chuang C.-H., Lin Y.-C., Liao J.-W., Lii C.-K., Yeh S.-L. (2018). Quercetin Enhances the Antitumor Effect of Trichostatin A and Suppresses Muscle Wasting in Tumor-Bearing Mice. Food Funct..

[177] Lee C.S., Jang E.-R., Kim Y.J., Myung S.C., Kim W. (2010). Casein Kinase 2 Inhibition Differentially Modulates Apoptotic Effect of Trichostatin A against Epithelial Ovarian Carcinoma Cell Lines. Mol. Cell. Biochem..

[178] Lee C.S., Yang J.C., Kim Y.J., Jang E.-R., Kim W., Myung S.C. (2010). 18β-Glycyrrhetinic Acid Potentiates Apoptotic Effect of Trichostatin A on Human Epithelial Ovarian Carcinoma Cell Lines. Eur. J. Pharmacol..

[179] Wu T.-C., Yang Y.-C., Huang P.-R., Wen Y.-D., Yeh S.-L. (2012). Genistein Enhances the Effect of Trichostatin A on Inhibition of A549 Cell Growth by Increasing Expression of TNF Receptor-1. Toxicol. Appl. Pharmacol..

[180] Chen Z., Clark S., Birkeland M., Sung C.-M., Lago A., Liu R., Kirkpatrick R., Johanson K., Winkler J.D., Hu E. (2002). Induction and Superinduction of Growth Arrest and DNA Damage Gene 45 (GADD45) α and β Messenger RNAs by Histone Deacetylase Inhibitors Trichostatin A (TSA) and Butyrate in SW620 Human Colon Carcinoma Cells. Cancer Lett..

[181] Wang B.X., Yin B.L., He B., Chen C., Zhao M., Zhang W.X., Xia Z.K., Pan Y.Z., Tang J.Q., Zhou X.M. (2012). Overexpression of DNA Damage-Induced 45 α Gene Contributes to Esophageal Squamous Cell Cancer by Promoter Hypomethylation. J. Exp. Clin. Cancer Res..

[182] Min K.N., Cho M.J., Kim D.-K., Sheen Y.Y. (2004). Estrogen Receptor Enhances the Antiproliferative Effects of Trichostatin A and HC-Toxin in Human Breast Cancer Cells. Arch. Pharmacal Res..

[183] Kang J., Zhang D., Chen J., Liu Q., Lin C. (2005). Antioxidants and Trichostatin A Synergistically Protect against *in Vitro* Cytotoxicity of Ni2+ in Human Hepatoma Cells. Toxicology.

[184] Kang J., Chen J., Zhang D., Da W., Ou Y. (2004). Synergistic Killing of Human Leukemia Cells by Antioxidants and Trichostatin A. Cancer Chemother. Pharm..

[185] Grigorov B. (2012). Reactive Oxygen Species and Their Relation to Carcinogenesis. Trakia J. Sci..

[186] Maxhimer J.B., Reddy R.M., Zuo J., Cole G.W., Schrump D.S., Nguyen D.M. (2005). Induction of Apoptosis of Lung and Esophageal Cancer Cells Treated with the Combination of Histone Deacetylase Inhibitor (Trichostatin A) and Protein Kinase C Inhibitor (Calphostin C). J. Thorac. Cardiovasc. Surg..

[187] Jeon H.G., Yoon C.Y., Yu J.H., Park M.J., Lee J.E., Jeong S.J., Hong S.K., Byun S.-S., Lee S.E. (2011). Induction of Caspase Mediated Apoptosis and Down-Regulation of Nuclear Factor-ΚB and Akt Signaling Are Involved in the Synergistic Antitumor Effect of Gemcitabine and the Histone Deacetylase Inhibitor Trichostatin A in Human Bladder Cancer Cells. J. Urol..

[188] Hamner J.B., Sims T.L., Cutshaw A., Dickson P.V., Rosati S., McGee M., Ng C.Y., Davidoff A.M. (2008). The Efficacy of Combination Therapy Using Adeno-Associated Virus—Interferon β and Trichostatin A *in Vitro* and in a Murine Model of Neuroblastoma. J. Pediatr. Surg..

[189] Hřebačková J., Poljakova J., Eckschlager T., Hraběta J., Prochazka P., Smutnỳ S., Stiborova M. (2009). Histone Deacetylase Inhibitors Valproate and Trichostatin A Are Toxic to Neuroblastoma Cells and Modulate Cytochrome P450 1A1, 1B1 and 3A4 Expression in These Cells. Interdiscip. Toxicol..

[190] Jin X., Fang Y., Hu Y., Chen J., Liu W., Chen G., Gong M., Wu P., Zhu T., Wang S. (2017). Synergistic Activity of the Histone Deacetylase Inhibitor Trichostatin A and the Proteasome Inhibitor PS-341 against Taxane-Resistant Ovarian Cancer Cell Lines. Oncol. Lett..

[191] Montagut C., Settleman J. (2009). Targeting the RAF–MEK–ERK pathway in cancer therapy. Cancer Lett..

[192] Yan G., Graham K., Lanza-Jacoby S. (2013). Curcumin Enhances the Anticancer Effects of Trichostatin a in Breast Cancer Cells. Mol. Carcinog..

[193] Piao J., Chen L., Quan T., Li L., Quan C., Piao Y., Jin T., Lin Z. (2016). Superior Efficacy of Co-Treatment with the Dual PI3K/MTOR Inhibitor BEZ235 and Histone Deacetylase Inhibitor Trichostatin A against NSCLC. Oncotarget.

[194] Chen L., Jin T., Zhu K., Piao Y., Quan T., Quan C., Lin Z. (2017). PI3K/MTOR Dual Inhibitor BEZ235 and Histone Deacetylase Inhibitor Trichostatin A Synergistically Exert Anti-Tumor Activity in Breast Cancer. Oncotarget.

[195] Bai J., Demirjian A., Sui J., Marasco W., Callery M.P. (2006). Histone Deacetylase Inhibitor Trichostatin A and Proteasome Inhibitor PS-341 Synergistically Induce Apoptosis in Pancreatic Cancer Cells. Biochem. Biophys. Res. Commun..

[196] Baek S.-Y., Kim S.-R., Hwang J.-W., Bae M.-K., Wee H.-J., Choi Y.-H., Oh S.-O., Kim B.-S., Yoon S., Bae S.-K. (2006). Combined Treatment of Trichostatin A and Heat Shock Increases Apoptosis in STAT3 Dependent Astrocytoma Cells. Cancer Prev. Res..

[197] Baek S.-Y., Kim S.-R., Bae M.-K., Hwang J.-W., Kim J.-S., Choi Y.H., Wee H.-J., Kim B.-S., Kim J.-B., Yoon S. (2006). Trichostatin A Increases the Thermosensitivity of Human Glioblastoma A172 Cells. Neurosci. Lett..

[198] Chen J., Bai H., Wang C., Kang J. (2006). Trichostatin A Improves the Anticancer Activity of Low Concentrations of Curcumin in Human Leukemia Cells. Cell Death.

[199] Piotrowska H., Jagodzinski P.P. (2007). Trichostatin A, Sodium Butyrate, and 5-Aza-2′-Deoxycytidine Alter the Expression of Glucocorticoid Receptor α and β Isoforms in Hut-78 T- and Raji B-Lymphoma Cell Lines. Biomed. Pharmacother..

[200] Jiang S.-J., Zhang S., Mu X.-Y., Li W., Wang Y. (2008). Effects of Trichostatin A and Paclitaxel on Apoptosis and Microtubule Stabilization in Endometrial Carcinoma Cells: An *in Vitro* Research. Zhonghua Yi Xue Za Zhi.

[201] Liu T.-C., Castelo-Branco P., Rabkin S.D., Martuza R.L. (2008). Trichostatin A and Oncolytic HSV Combination Therapy Shows Enhanced Antitumoral and Antiangiogenic Effects. Mol. Ther..

[202] Liu Z., Marquez M., Nilsson S., Holmberg A. (2008). Incubation with Somatostatin, 5-Aza Decitabine and Trichostatin up-Regulates Somatostatin Receptor Expression in Prostate Cancer Cells. Oncol. Rep..

[203] Lu M.-C., Du Y.-C., Chuu J.-J., Hwang S.-L., Hsieh P.-C., Hung C.-S., Chang F.-R., Wu Y.-C. (2009). Active Extracts of Wild Fruiting Bodies of Antrodia Camphorata (EEAC) Induce Leukemia HL 60 Cells Apoptosis Partially through Histone Hypoacetylation and Synergistically Promote Anticancer Effect of Trichostatin A. Arch. Toxicol..

[204] Hrabeta J., Poljakova J., Frei E., Stiborova M., Eckschlager T. Inhibitors of Histone Deacetylase, Valproic Acid and Trichostatin A, Increase Cytotoxicity of Anticancer Drug Ellipticine to Neuroblastoma Cells. Proceedings of the Cancer Research.

[205] Cecconi D., Donadelli M., Dalla Pozza E., Rinalducci S., Zolla L., Scupoli M.T., Righetti P.G., Scarpa A., Palmieri M. (2009). Synergistic Effect of Trichostatin A and 5-Aza-2′-Deoxycytidine on Growth Inhibition of Pancreatic Endocrine Tumour Cell Lines: A Proteomic Study. Proteomics.

[206] Pouliot K. The Histone Deacetylase Inhibitors, Trichostatin A and Apicidin, Enhance the Radiosensitivity of Ovarian Carcinoma Cells *in Vitro*. Proceedings of the Cancer Research.

[207] Shiau R.-J., Chen K.-Y., Wen Y.-D., Chuang C.-H., Yeh S.-L. (2010). Genistein and β-Carotene Enhance the Growth-Inhibitory Effect of Trichostatin A in A549 Cells. Eur. J. Nutr..

[208] Poljakova J., Hrebackova J., Dvorakova M., Moserova M., Eckschlager T., Hrabeta J., Göttlicherova M., Kopejtkova B., Frei E., Kizek R. (2011). Anticancer Agent Ellipticine Combined with Histone Deacetylase Inhibitors, Valproic Acid and Trichostatin A, Is an Effective DNA Damage Strategy in Human. Neuroblastoma.

[209] Jiang L., Lian M., Wang H., Fang J., Wang Q. (2012). Inhibitory Effects of 5-Aza-2′-Deoxycytidine and Trichostatin A in Combination with P53-Expressing Adenovirus on Human Laryngocarcinoma Cells. Chin. J. Cancer Res..

[210] Tu Z., Li H., Ma Y., Tang B., Tian J., Akers W., Achilefu S., Gu Y. (2012). The Enhanced Antiproliferative Response to Combined Treatment of Trichostatin A with Raloxifene in MCF-7 Breast Cancer Cells and Its Relevance to Estrogen Receptor β Expression. Mol. Cell. Biochem..

[211] Zhang S., Zhang Q., Jiang S. (2013). Effect of Trichostatin A and Paclitaxel on the Proliferation and Apoptosis of Lung Adenocarcinoma Cells. Chin. Med. J..

[212] Tran H.T.T., Kim H.N., Lee I.-K., Nguyen-Pham T.-N., Ahn J.-S., Kim Y.-K., Lee J.-J., Park K.-S., Kook H., Kim H.-J. (2013). Improved Therapeutic Effect against Leukemia by a Combination of the Histone Methyltransferase Inhibitor Chaetocin and the Histone Deacetylase Inhibitor Trichostatin A. J. Korean Med. Sci..

[213] Duo J., Ma Y., Wang G., Han X., Zhang C. (2013). Metformin Synergistically Enhances Antitumor Activity of Histone Deacetylase Inhibitor Trichostatin A Against Osteosarcoma Cell Line. DNA Cell Biol..

[214] Kiliccioglu I., Konac E., Varol N., Gurocak S., Yucel Bilen C. (2014). Apoptotic Effects of Proteasome and Histone Deacetylase Inhibitors in Prostate Cancer Cell Lines. Genet. Mol. Res..

[215] Asgar M.A., Senawong G., Sripa B., Senawong T. (2016). Synergistic Anticancer Effects of Cisplatin and Histone Deacetylase Inhibitors (SAHA and TSA) on Cholangiocarcinoma Cell Lines. Int. J. Oncol..

[216] Du R., Liu Z., Hou X., Fu G., An N., Wang L. (2016). Trichostatin A Potentiates Genistein-Induced Apoptosis and Reverses EMT in HEp2 Cells. Mol. Med. Rep..

[217] Ghosh S., Jayaram P., Kabekkodu S.P., Satyamoorthy K. (2022). Targeted drug delivery in cervical cancer: Current perspectives. Eur. J. Pharmacol..

[218] Mazzio E.A., Soliman K.F.A. (2017). HTP Nutraceutical Screening for Histone Deacetylase Inhibitors and Effects of HDACis on Tumor-Suppressing MiRNAs by Trichostatin A and Grapeseed (Vitis Vinifera) in HeLa Cells. Cancer Genom. Proteom..

[219] Gilardini Montani M.S., Granato M., Santoni C., Del Porto P., Merendino N., D’Orazi G., Faggioni A., Cirone M. (2017). Histone Deacetylase Inhibitors VPA and TSA Induce Apoptosis and Autophagy in Pancreatic Cancer Cells. Cell. Oncol..

[220] Chen R., Yang Y., Xu J., Pan Y., Zhang W., Xing Y., Ni H., Sun Y., Hou Y., Li N. (2018). Tamarix Hohenackeri Bunge Exerts Anti-Inflammatory Effects on Lipopolysaccharide-Activated Microglia *in Vitro*. Phytomedicine.

[221] Sun P., Zhou X., He Y., Liu H., Wang Y., Chen Y., Li M., He Y., Li G., Li Y. (2018). Effect of Trichostatin A on Burkitt’s Lymphoma Cells: Inhibition of EPS8 Activity through Phospho-Erk1/2 Pathway. Biochem. Biophys. Res. Commun..

[222] Wu N., Zhu Y., Xu X., Zhu Y., Song Y., Pang L., Chen Z. (2018). The Anti-Tumor Effects of Dual PI3K/MTOR Inhibitor BEZ235 and Histone Deacetylase Inhibitor Trichostatin A on Inducing Autophagy in Esophageal Squamous Cell Carcinoma. J. Cancer.

[223] Chuang C.-H., Chan S.-T., Chen C.-H., Yeh S.-L. (2019). Quercetin Enhances the Antitumor Activity of Trichostatin A through Up-Regulation of P300 Protein Expression in P53 Null Cancer Cells. Chem. Biol. Interact..

[224] Hsu F.-S., Wu J.-T., Lin J.-Y., Yang S.-P., Kuo K.-L., Lin W.-C., Shi C.-S., Chow P.-M., Liao S.-M., Pan C.-I. (2019). Histone Deacetylase Inhibitor, Trichostatin A, Synergistically Enhances Paclitaxel-Induced Cytotoxicity in Urothelial Carcinoma Cells by Suppressing the ERK Pathway. Int. J. Mol. Sci..

[225] Ren C., Gao C., Li X., Xiong J., Shen H., Wang L., Zhu D., Wu P., Ding W., Wang H. (2020). The Antitumor Efficiency of Zinc Finger Nuclease Combined with Cisplatin and Trichostatin A in Cervical Cancer Cells. Anti-Cancer Agents Med. Chem..

[226] Wong S.H.M., Fang C.-M., Loh H.-S., Ngai S.C. (2021). Trichostatin A and Zebularine along with E-Cadherin Re-Expression Enhance Tumour Necrosis Factor-Related Apoptosis-Inducing Ligand (TRAIL)-Mediated Cell Cycle Arrest in Human Breast Adenocarcinoma Cells. Asia Pac. J. Mol. Biol. Biotechnol..

[227] Ou J.-N., Torrisani J., Unterberger A., Provençal N., Shikimi K., Karimi M., Ekström T.J., Szyf M. (2007). Histone Deacetylase Inhibitor Trichostatin A Induces Global and Gene-Specific DNA Demethylation in Human Cancer Cell Lines. Biochem. Pharmacol..

[228] Li Y., Meeran S.M., Patel S.N., Chen H., Hardy T.M., Tollefsbol T.O. (2013). Epigenetic Reactivation of Estrogen Receptor-α (ERα) by Genistein Enhances Hormonal Therapy Sensitivity in ERα-Negative Breast Cancer. Mol. Cancer.

[229] Meng F., Sun G., Zhong M., Yu Y., Brewer M.A. (2013). Inhibition of DNA Methyltransferases, Histone Deacetylases and Lysine-Specific Demethylase-1 Suppresses the Tumorigenicity of the Ovarian Cancer Ascites Cell Line SKOV3. Int. J. Oncol..

[230] Koh E., Bandle R., Clair T., Roberts D.D., Stracke M.L. (2007). Trichostatin A and 5-Aza-2′-Deoxycytidine Switch S1P from an Inhibitor to a Stimulator of Motility through Epigenetic Regulation of S1P Receptors. Cancer Lett..

[231] Choi J.-H., Min N.Y., Park J., Kim J.H., Park S.H., Ko Y.J., Kang Y., Moon Y.J., Rhee S., Ham S.W. (2010). TSA-Induced DNMT1 down-Regulation Represses HTERT Expression via Recruiting CTCF into Demethylated Core Promoter Region of HTERT in HCT116. Biochem. Biophys. Res. Commun..

[232] Sanaei M., Kavoosi F. (2018). Effect of Curcumin and Trichostatin A on the Expression of DNA Methyltransfrase 1 in Hepatocellular Carcinoma Cell Line Hepa 1-6. Iran. J. Pediatr. Hematol. Oncol..

[233] Januchowski R., Dąbrowski M., Ofori H., Jagodzinski P.P. (2007). Trichostatin A Down-Regulate DNA Methyltransferase 1 in Jurkat T Cells. Cancer Lett..

[234] Wang H., Li Q., Chen H. (2012). Genistein Affects Histone Modifications on Dickkopf-Related Protein 1 (DKK1) Gene in SW480 Human Colon Cancer Cell Line. PLoS ONE.

[235] Wu D.-S., Shen J.-Z., Yu A.-F., Fu H.-Y., Zhou H.-R., Shen S.-F. (2013). Epigallocatechin-3-Gallate and Trichostatin A Synergistically Inhibit Human Lymphoma Cell Proliferation through Epigenetic Modification of P16INK4a. Oncol. Rep..

